# The effects of improving sleep on mental health (OASIS): a randomised controlled trial with mediation analysis

**DOI:** 10.1016/S2215-0366(17)30328-0

**Published:** 2017-10

**Authors:** Daniel Freeman, Bryony Sheaves, Guy M Goodwin, Ly-Mee Yu, Alecia Nickless, Paul J Harrison, Richard Emsley, Annemarie I Luik, Russell G Foster, Vanashree Wadekar, Christopher Hinds, Andrew Gumley, Ray Jones, Stafford Lightman, Steve Jones, Richard Bentall, Peter Kinderman, Georgina Rowse, Traolach Brugha, Mark Blagrove, Alice M Gregory, Leanne Fleming, Elaine Walklet, Cris Glazebrook, E Bethan Davies, Chris Hollis, Gillian Haddock, Bev John, Mark Coulson, David Fowler, Katherine Pugh, John Cape, Peter Moseley, Gary Brown, Claire Hughes, Marc Obonsawin, Sian Coker, Edward Watkins, Matthias Schwannauer, Kenneth MacMahon, A Niroshan Siriwardena, Colin A Espie

**Affiliations:** a Department of Psychiatry, University of Oxford, Warneford Hospital, Oxford, UK; b Oxford Health National Health Service (NHS) Foundation Trust, Oxford, UK; c Nuffield Department of Primary Care Health Sciences, University of Oxford, Oxford, UK; d Centre for Biostatistics, Institute of Population Health, Manchester University, Manchester Academic Health Centre, Manchester, UK; e Sleep and Circadian Neuroscience Institute, Nuffield Department of Clinical Neurosciences, University of Oxford, Sir William Dunn School of Pathology, Oxford, UK; f Big Health Ltd, London, UK; g Institute of Health and Wellbeing, University of Glasgow, Gartnavel Royal Hospital, Glasgow, UK; h School of Nursing and Midwifery, Faculty of Health and Human Sciences, Plymouth University, Plymouth, UK; i Henry Wellcome Laboratories for Integrative Neuroscience and Endocrinology, University of Bristol, Bristol, UK; j Spectrum Centre for Mental Health Research, Faculty of Health and Medicine, Lancaster University, Lancaster, UK; k Psychological Sciences, Institute of Psychology Health and Society, University of Liverpool, Liverpool, UK; l Clinical Psychology Unit, Department of Psychology, University of Sheffield, Sheffield, UK; m Department of Health Sciences, College of Medicine, Biological Sciences and Psychology, Centre for Medicine, University of Leicester, Leicester, UK; n Department of Psychology, University of Swansea, Swansea, UK; o Department of Psychology, Goldsmiths, University of London, London, UK; p Department of Psychology, University of Strathclyde, Glasgow, UK; q Department of Psychology, Institute of Health and Society, University of Worcester, Worcester, UK; r Division of Psychiatry and Applied Psychology, School of Medicine, University of Nottingham, Nottingham, UK; s National Institute for Healthcare Research MindTech Healthcare Technology Co-operative, Institute of Mental Health, University of Nottingham, Nottingham, UK; t Division of Psychology and Mental Health, School of Health Sciences, University of Manchester, Manchester, UK; u School of Psychology and Therapeutic Studies, University of South Wales, Treforest, UK; v Department of Psychology, School of Science and Technology, University of Middlesex, London, UK; w Department of Psychology, University of Sussex, Brighton, UK; x Sussex Partnership NHS Foundation Trust, Worthing, UK; y Department of Clinical, Educational and Health Psychology, University College London, London, UK; z School of Psychology, University of Central Lancashire, Preston, UK; aa Psychology Department, Royal Holloway, Egham, UK; ab University of Cambridge Centre for Family Research, Cambridge, UK; ac School of Psychological Sciences and Health, University of Strathclyde, Glasgow UK; ad Department of Clinical Psychology, Norwich Medical School, University of East Anglia, Norwich, UK; ae SMART Lab, College of Life and Environmental Sciences, Sir Henry Wellcome Building for Mood Disorders Research, University of Exeter, Exeter, UK; af School of Health in Social Sciences, University of Edinburgh, Medical School, Edinburgh, UK; ag Community and Health Research Unit, University of Lincoln, Lincoln, UK

## Abstract

**Background:**

Sleep difficulties might be a contributory causal factor in the occurrence of mental health problems. If this is true, improving sleep should benefit psychological health. We aimed to determine whether treating insomnia leads to a reduction in paranoia and hallucinations.

**Methods:**

We did this single-blind, randomised controlled trial (OASIS) at 26 UK universities. University students with insomnia were randomly assigned (1:1) with simple randomisation to receive digital cognitive behavioural therapy (CBT) for insomnia or usual care, and the research team were masked to the treatment. Online assessments took place at weeks 0, 3, 10 (end of therapy), and 22. The primary outcome measures were for insomnia, paranoia, and hallucinatory experiences. We did intention-to-treat analyses. The trial is registered with the ISRCTN registry, number ISRCTN61272251.

**Findings:**

Between March 5, 2015, and Feb 17, 2016, we randomly assigned 3755 participants to receive digital CBT for insomnia (n=1891) or usual practice (n=1864). Compared with usual practice, the sleep intervention at 10 weeks reduced insomnia (adjusted difference 4·78, 95% CI 4·29 to 5·26, Cohen's d=1·11; p<0·0001), paranoia (−2·22, −2·98 to −1·45, Cohen's d=0·19; p<0·0001), and hallucinations (−1·58, −1·98 to −1·18, Cohen's d=0·24; p<0·0001). Insomnia was a mediator of change in paranoia and hallucinations. No adverse events were reported.

**Interpretation:**

To our knowledge, this is the largest randomised controlled trial of a psychological intervention for a mental health problem. It provides strong evidence that insomnia is a causal factor in the occurrence of psychotic experiences and other mental health problems. Whether the results generalise beyond a student population requires testing. The treatment of disrupted sleep might require a higher priority in mental health provision.

**Funding:**

Wellcome Trust.

## Introduction

Sleep problems are a common occurrence in patients with mental health disorders. The traditional view is that disrupted sleep is a symptom, consequence, or non-specific epiphenomenon of the disorders; the clinical result is that the treatment of sleep problems is given a low priority. An alternative perspective is that disturbed sleep is a contributory causal factor in the occurrence of many mental health disorders.^1^ An escalating cycle then emerges between the distress of the mental health symptoms, effect on daytime functioning, and struggles in gaining restorative sleep. From this alternative perspective, the treatment of sleep problems attains a higher clinical importance. We are particularly interested in the putative causal association between disturbed sleep and psychotic experiences.^2^, ^3^ The interventionist–causal model approach to establishment of a causal association is to manipulate the hypothesised mechanistic variable and observe the effect on the outcome of interest;^4^ if a causal association exists then the outcome variable should alter. The effects of the manipulation can then be substantiated further by use of mediation analysis.^5^, ^6^ In the present study, we aimed to improve sleep in individuals with insomnia to determine the effect on psychotic experiences. This approach therefore informs both theoretical understanding and clinical practice.

The most common form of sleep disruption is insomnia, comprising sustained difficulties in initiating or staying asleep, or both, which cause problems during the day. The association of insomnia with psychotic experiences in the general population has been established.^3^ There are multiple, independent, psychotic experiences. Each psychotic experience exists on a spectrum of severity in the general population with differing heritability and differing strength of association with insomnia.^7^ Paranoia and hallucinations have the strongest links with insomnia.^2^, ^7^, ^8^ However, the effect of altering the amount of sleep disruption—eg, by targeted sleep treatment—on these psychotic experiences remains to be established. Clinical guidelines recommend the use of cognitive behavioural therapy (CBT) as the first-line treatment for insomnia.^9^ Digital forms of CBT for insomnia that require no therapist to be present have been shown to be efficacious as well.^10^, ^11^, ^12^ In patients with current delusions and hallucinations, results of our pilot randomised controlled trial^13^ have shown that insomnia can be substantially reduced with CBT, but the trial was underpowered to establish with sufficient precision the consequences for psychotic experiences. Therefore, we undertook a clinical trial that was large enough to definitively test the causal association between insomnia and self-reported psychotic experiences.

**Research in context**

**Evidence before this study**

If insomnia is a contributory cause of psychotic experiences, then the key test is whether improving sleep will lead to a reduction in psychotic experiences. We therefore searched for randomised controlled studies that set out to reduce insomnia and examine the effects on psychotic experiences. On June 23, 2017, we searched the entire archive (ie, using no date restrictions) of PubMed for: (Sleep OR Insomnia) AND (Delus* OR Hallucinat* OR Psychosis OR Psychotic OR Schizophren*) AND (CBT OR hypnotic OR medication) AND (Random* OR RCT). 130 papers were identified and only two were randomised controlled trials that tested the effects of sleep treatment on psychotic experiences, with the larger of the trials being our own with 50 patients with schizophrenia or related disorders. These trials were underpowered to determine with any precision the potential link between insomnia and psychotic experiences.

**Added value of this study**

We undertook what might be the largest randomised controlled trial to date of a psychological treatment. It is the first study adequately powered to determine the effects of treating sleep dysfunction on psychotic experiences. It shows very clearly that treatment of insomnia in students leads to a reduction in psychotic experiences. A mediation analysis supports this interpretation. Furthermore, the trial is consistent with a small number of other randomised controlled trials that indicate multiple other benefits for mental health when treating sleep problems.

**Implications of all the available evidence**

Sleep disruption might have a contributory causal role in the occurrence of psychotic experiences and a wide range of other mental health problems. Adequately powered tests in other populations would be helpful, but research indicating that the treatment of disrupted sleep requires a higher priority in mental health provision is accumulating.

To test thousands of individuals, we did an online study using digital CBT for insomnia treatment. A participant pool (university students) was selected that would be easily reachable (since we would have access to large email lists) and at an age when mental health disorders emerge. We have previously shown in a student population that sleep problems are associated with elevated levels of paranoia, hallucinations, anxiety, depression, and manic symptoms.^8^ Our main aim required a comparison between the effects of a reduction in insomnia (in a group receiving recommended treatment) and continued sleep disruption (in a group receiving usual care, which is likely to mean receiving little or no treatment). Clear change in sleep in one group relative to another was required to test the mechanistic hypothesis. We were not investigating the intervention elements that might lead to change. Our primary aim was to find out whether CBT for insomnia, compared with a usual practice control group, reduced insomnia and reduced paranoia and hallucinations by the end of treatment, and whether the changes in insomnia mediated the changes in psychotic experiences. We also aimed to determine the potential effects of sleep improvement on a wider range of mental health outcomes in this general population group. Our secondary aims were to investigate whether digital CBT for insomnia, compared with usual practice, reduced depression, anxiety, nightmares, and mania; improved psychological wellbeing; and led to the occurrence of fewer mental health disorders.

## Methods

### Study design and participants

We did this single-blind, randomised controlled trial (OASIS; Oxford Access for Students Improving Sleep) of digital CBT versus treatment as usual (usual practice). Screening, informed consent, assessments, allocation to condition, and the delivery of the intervention were carried out online using an automated system, a specially configured instance of True Colours, which is a system for the scheduled collection of outcome measures.^14^ Participants in the control group were given access to the sleep intervention after their final assessment. The study received overall ethical approval from the University of Oxford Medical Sciences Inter-Divisional Ethics Committee and then local approvals at the other participating universities. The OASIS trial protocol has been published.^15^

Participants were eligible if they were attending university; had a positive screen for insomnia, as indicated by a score of 16 or lower on the Sleep Condition Indicator (SCI);^16^ and were 18 years or older. We had no exclusion criteria. 26 UK universities took part (appendix 1), ensuring a range in geographical locations and academic ability. The principal method of recruitment was sending a circular email within universities that contained a link to the web-based screening. When a circular email was not possible, recruitment was via advertisment on websites and displaying posters, or both. Recruitment began on March 5, 2015, and ended on Feb 17, 2016. We collected the final data on July 28, 2016.

### Randomisation and masking

As recommended for large clinical trials,^17^ we used simple randomisation (1:1) with an automated online system, ensuring that the research team was unable to affect randomisation. Participants completed all the assessments independently online and therefore their responses could not be affected by the trial team.

### Procedures

Assessments took place at weeks 0 (baseline), 3, 10 (end of therapy), and 22. The week 3 assessment comprised only the primary outcome measures. The week 3 assessment was carried out to assess in the mediation analyses the temporal order of changes. Participants received an email prompt to complete the assessments online. The order of the assessments was consistent across timepoints. If participants did not complete the assessment, then they received up to two email reminders 2 days apart.

The CBT for insomnia intervention is called Sleepio.^11^, ^18^ It is provided in six sessions, lasting an average of 20 min each. Sessions are unlocked weekly, although participants can move at a slower pace. The full programme is accessible via any web browser and all participants start the programme online. Certain tools (eg, sleep diaries and relaxation audios) could also be accessed using the web browser of a smartphone. All of the sessions, sleep diaries, relaxation audios, and the scheduling tool could be accessed with an iPhone. Completion of an initial assessment drives the algorithms that personalise the programme. For example, the assessment leads to a tailored choice of treatment goal, with progress then reviewed at each subsequent session. The treatment includes behavioural, cognitive, and educational components. The behavioural techniques include sleep restriction (ie, reducing the sleep window to enhance sleep consolidation), stimulus control (eg, getting out of bed after 15–20 min of wakefulness), and relaxation (eg, tensing and relaxing muscles when in bed). The cognitive techniques include paradoxical intention (eg, trying to stay awake), belief restructuring (eg, targeting unrealistic expectations about sleep), mindfulness (eg, acknowledging thoughts and feelings without dwelling on them), imagery (eg, generating positive mental images), and putting the day to rest (eg, setting time aside to reflect on the day). The educational component covers information about the processes of sleep and sleep hygiene. The programme is interactive, and content is presented by an animated therapist. Participants make a time for the session and are prompted via email or text message via a short message service if they do not attend. Participants complete daily sleep diaries throughout the intervention, which are used by the programme to tailor the advice. Sleep restriction is introduced in the third session of the course. The animated therapist proposes a new sleep window, which is calculated from the sleep diary data, and engages with the participant to help them select the timing of the window (eg, earlier versus later in the night). A more lenient sleep window is used for those individuals reporting substantial physical problems, other mental health problems, or moderate-to-severe sleepiness. The sleep window is regularly reviewed at each session after it has been introduced. If the sleep diary data indicate a sleep efficiency of 90% or higher, the animated therapist advises that 15 min is added to the sleep window. Throughout the course of therapy, participants had access to a moderated online community and an online library of information about sleep. Participants could also view their online case file, which included four sections: a progress review, a reminder of strategies, an agreed sleep schedule, and a list of further reading. Usual practice (treatment as usual) referred to the current care that the participants were receiving. The amount of treatment input was likely to be minimal, with prescription of medication for a small proportion. We did not attempt to alter the current care that participants received.

### Outcomes

The primary outcomes were insomnia, paranoia, and hallucinatory experiences. The primary measure for insomnia was the SCI-8.^16^ This score is an 8-item measure, validated against DSM-5 criteria, assessing sleep and its impact on daytime functioning over the past week. Scores can range from 0 to 32 with higher scores indicating better sleep. A clinical cutoff of less than 17 correctly identifies 89% of individuals with probable insomnia disorder. We used a version of the SCI that included one additional question, as a secondary outcome, regarding early morning waking. The internal consistency (Cronbach's α at baseline) of the scale in the present study was 0·63.

Paranoia was assessed with the Green et al Paranoid Thought Scales (GPTS), part B.^19^ This scale assesses persecutory ideation, and the timeframe used was the past fortnight. The scale comprises 16 items, each rated on a 1 (not at all) to 5 (totally) scale. High scores indicate higher levels of paranoia. The internal consistency of the scale in the present study was 0·94.

The measure for hallucinations was the Specific Psychotic Experiences Questionnaire—Hallucinations subscale.^20^ The scale comprises nine items rated on a 0 (not at all) to 5 (more than once per day) scale. The timeframe was the past fortnight. Higher scores indicate greater occurrences of hallucinatory experiences. The internal consistency of the scale in the present study was 0·93.

The secondary outcome measures for sleep were the Insomnia Severity Index (ISI;^21^ Cronbach's α in the present study=0·67) and the Disturbing Dreams and Nightmare Severity Index^22^ (Cronbach's α in the present study=0·91). The secondary outcome measure for psychotic experiences was the 16-item version of the Prodromal Questionnaire^23^ (Cronbach's α in the present study=0·79). A score of 6 or more has 87% specificity and 87% sensitivity to correctly classify ultra-high risk for psychosis mental states in a help-seeking sample.

The measures to assess affective symptoms were the Patient Health Questionnaire 9-item version^24^ (PHQ-9; Cronbach's α in the present study=0·85), the Generalised Anxiety Disorder 7-item version^25^ (GAD-7; Cronbach's α in the present study=0·89), and the Altman Mania Scale^26^ (Cronbach's α in the present study=0·64). Psychological wellbeing was assessed with the Warwick–Edinburgh Mental Wellbeing Scale (WEMWBS;^27^ Cronbach's α in the present study=0·89), and the Work and Social Adjustment Scale (WSAS;^28^ Cronbach's α in the present study=0·83).

To assess the development of mental health disorders, we used established cutoffs on the Prodromal Questionnaire,^23^ Altman Mania Scale,^26^ PHQ,^24^ and GAD-7.^25^ Participants were also asked at each assessment timepoint whether they were in contact with mental health services, had received a mental health diagnosis, took medication for a mental health problem, or were currently receiving any other psychological therapy.

If the trial team were informed of the occurrence of a serious adverse event for a trial participant, then this was recorded. Serious adverse events were defined as deaths, suicide attempts, serious violent incidents, admissions to secure units, and formal complaints about the online intervention.

### Statistical analysis

We calculated the sample size on the basis of change in paranoia (one of the two psychotic symptoms studied), since following this intervention change would be expected to be lower in psychotic experiences than in insomnia. Based on the SDs observed from a previous study^29^ for the GPTS (SD 10·4), a total sample size of 2614 participants (ie, 1307 per group) would provide 90% power to detect a small effect size in paranoia, with a standardised mean difference of 0·15, while accounting for a high amount of expected attrition (40%). In a study amendment, the sample size was increased because of a higher than initially expected dropout rate.

An outline of the analysis strategy was provided in the published trial protocol^15^ and a full statistical analysis plan was agreed before the trial analysis (appendix 2). All the analyses were validated by a second statistician. Analyses were by intention to treat and were carried out at the end of the last follow-up assessment (with no interim analyses). We analysed each continuous outcome with a linear mixed effects regression model to account for the repeated measures over time, and we analysed binary outcomes with a logistic mixed effects model. Mixed effects models are the recommended statistical technique for analysing clinical trials when outcomes are collected at repeated timepoints,^30^ and in this trial included outcome data at weeks 3, 10, and 22 available for all participants who had been randomly assigned. The method has the advantage of implicitly accounting for data missing at random. The estimated (adjusted) treatment differences from these analyses are therefore reported. The linear mixed effects models included the outcome as the response variable, timepoint, randomised group, and baseline score as fixed effects and random effects were estimated for students nested within universities. A student is located within one university, and so to estimate the random intercepts, we accounted for random variation between universities and between students within the same university. We modelled an interaction between time and randomised group as a fixed effect to allow estimation of treatment effect at all three timepoints. Sex and course level were included as covariates in the model. We used an unstructured variance–covariance matrix to model the within-subject error correlation structure. Results are presented as mean adjusted differences in scores between the randomised groups, with 95% CIs and associated two-sided p values. We confirmed the normality assumption of the residuals for each outcome. No deviations from normality were apparent and therefore maximum likelihood estimates were reported. We did sensitivity analyses (pattern mixture models, inclusion of baseline covariates predictive of missing data, and imputation) for the three main outcomes, examining the robustness of the results to different assumptions regarding missing data. We calculated standardised effect sizes with Cohen's d, dividing the treatment effect by the shared SD at baseline. We used similar logistic mixed effects models for the secondary binary outcomes.

To test the mediation hypotheses, we determined the extent of mediation of the week 3 and week 10 insomnia scores on the week 10 paranoia and hallucination outcomes. The approach used was similar to the method of Baron and Kenny,^5^, ^31^ but made use of linear mixed effects models at each step. The approach involved four steps and three separate model fits. In two separate linear mixed effects models, the intervention was shown to be correlated with the outcome and then with the mediator. We then fitted the data to a third model with the outcome as the response and both the intervention and mediator as covariates. The parameters were extracted as per Baron and Kenny^31^ to obtain the total, direct, and indirect effects, and finally the percentage mediation was determined. In all models, we included baseline amounts of both the outcome and mediator as covariates. This method is similar to the mediation analysis in the study by Freeman and colleagues,^32^ but made use of linear mixed effects models to account for repeated measurements, rather than through structural equation modelling. We used STATA version 14.1 for the statistical analysis.

The trial is registered with the ISRCTN registry, number ISRCTN61272251.

### Role of the funding source

The funder of the study had no role in study design, data collection, data analysis, data interpretation, or writing of the report. The corresponding author had full access to all the data in the study and had final responsibility for the decision to submit for publication.

## Results

Between March 5, 2015, and Feb 17, 2016, we randomly assigned 3755 participants to receive digital CBT for insomnia (n=1891) or usual practice (n=1864; figure). The sample was predominately female, studying for their first university degree, and two-thirds were of white British ethnicity (table 1). Around a fifth of the participants were in contact with mental health services (table 1). The two randomised groups were well matched at baseline (table 1).

**Figure:**
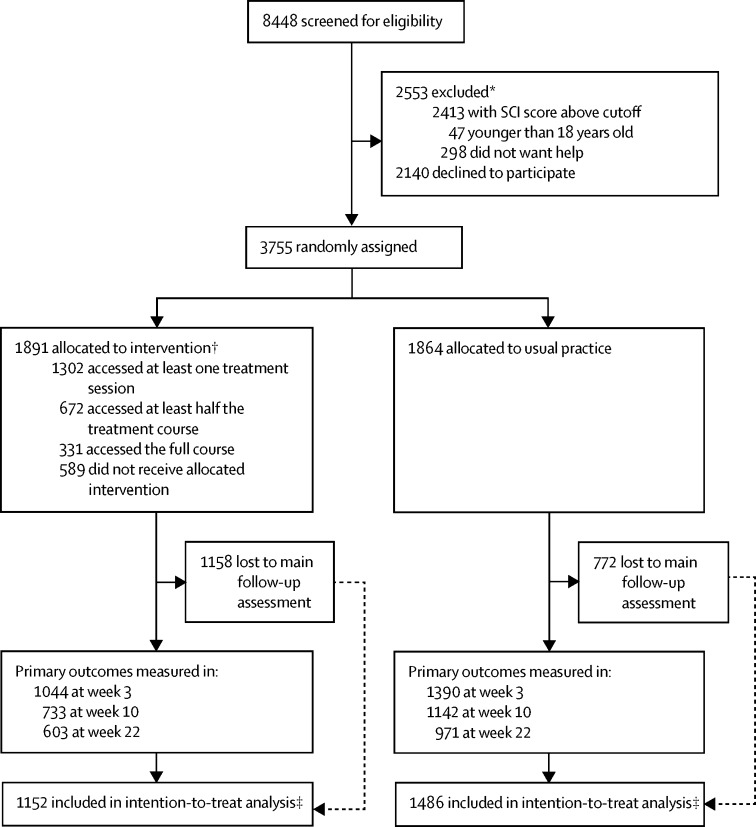
Trial profile SCI=Sleep Condition Indicator. *Some participants excluded for two or more reasons. †Not all participants accessed all treatment sessions. ‡Had at least one measurement at week 3, 10, or 22.

**Table 1:** Baseline characteristics

			**Control group (n=1864)**	**Treatment group (n=1891)**
Mean age (years)			24·6 (7·6)	24·8 (7·7)
Mean total UCAS points			753·0 (517·3)	720·8 (456·3)
Sex				
	Male		530 (28%)	513 (27%)
	Female		1315 (71%)	1361 (72%)
	Other		19 (1%)	17 (1%)
Course level				
	Undergraduate		1352 (73%)	1389 (73%)
	Postgraduate		480 (26%)	473 (25%)
	Other		32 (2%)	29 (2%)
Ethnic origin				
	White			
		British	1212 (65%)	1265 (67%)
		Irish	32 (2%)	27 (1%)
		Other	284 (15%)	261 (14%)
	Mixed			
		White and Caribbean	13 (1%)	11 (1%)
		White and African	9 (<1%)	13 (1%)
		White and Asian	31 (2%)	27 (1%)
		Other	36 (2%)	29 (2%)
	Asian			
		Indian	26 (1%)	43 (2%)
		Pakistani	23 (1%)	22 (1%)
		Bangladeshi	9 (<1%)	7 (<1%)
		Chinese	95 (5%)	73 (4%)
		Other	25 (1%)	32 (2%)
	Black			
		African	26 (1%)	23 (1%)
		Caribbean	10 (1%)	17 (1%)
		Other	2 (<1%)	3 (<1%)
	Arab		12 (1%)	14 (1%)
	Other		19 (1%)	24 (1%)
Mean insomnia (SCI-8) score			10·1 (4·3)	9·9 (4·3)
Mean paranoia (GPTS) score			24·8 (11·6)	25·4 (11·9)
Mean hallucinations (SPEQ) score			5·3 (6·9)	5·3 (6·4)
Mean insomnia (SCI-9) score			12·1 (4·9)	11·9 (4·8)
Mean insomnia (ISI) score			15·3 (4·0)	15·4 (3·9)
Mean nightmares (DDNSI) score			8·1 (8·2)	7·7 (7·8)
Mean prodromal psychosis (PQ-16) score			4·9 (3·4)	4·8 (3·3)
Mean depression (PHQ-9) score			12·7 (5·9)	12·9 (5·8)
Mean anxiety (GAD-7) score			9·0 (5·6)	9·4 (5·6)
Mean mania (Altman) score			3·5 (3·0)	3·5 (3·0)
Mean functioning (WSAS) score			17·7 (7·6)	17·6 (7·6)
Mean wellbeing (WEMWBS) score			37·9 (8·8)	37·8 (8·5)
Ultra-high risk of psychosis (PQ-16) cutoff (≥6)				
	Above		706 (38%)	711 (38%)
	Below		1158 (62%)	1180 (62%)
Depressive disorder (PHQ-9) cutoff (≥10)				
	Above		1238 (66%)	1286 (68%)
	Below		626 (34%)	605 (32%)
Anxiety disorder (GAD-7) cutoff (≥10)				
	Above		781 (42%)	880 (47%)
	Below		1083 (58%)	1011 (53%)
Mania disorder (Altman) score cutoff (≥6)				
	Above		422 (23%)	413 (22%)
	Below		1442 (77%)	1478 (78%)
Contact with mental health services				
	Yes		328 (18%)	346 (18%)
	No		1536 (82%)	1545 (82%)
Any psychiatric diagnosis				
	Yes		590 (32%)	641 (34%)
	No		1274 (68%)	1250 (66%)
Previous diagnosis of a sleep disorder				
	Yes		93 (5%)	96 (5%)
	No		1771 (95%)	1795 (95%)
Any psychiatric medication				
	Yes		433 (23%)	460 (24%)
	No		1431 (77%)	1431 (76%)
Specific medication for a sleep disorder				
	Yes		51 (3%)	55 (3%)
	No		1813 (97%)	1836 (97%)
Psychological therapy				
	Yes		146 (8%)	135 (7%)
	No		1718 (92%)	1756 (93%)

Data are mean (SD) or n (%). UCAS=Universities and Colleges Admissions Service. SCI-8=Sleep Condition Indicator 8-item version. GPTS=Green et al Paranoid Thought Scales. SPEQ=Specific Psychotic Experiences Questionnaire. SCI-9=SCI 9-item version. ISI=Insomnia Severity Index. DDNSI=Disturbing Dream and Nightmare Severity Index. PQ-16=Prodromal Questionnaire 16-item version. PHQ-9=Patient Health Questionnaire 9-item version. GAD-7=Generalised Anxiety Disorder 7-item version. WSAS=Work and Social Adjustment Scale. WEMWBS=Warwick–Edinburgh Mental Wellbeing Scale.

Appendix 1 has the full details of the sample, missing data patterns, sensitivity analyses, and all other analyses. The dropout from the study assessments was high (50%) during the course of the study, and was greater in the treatment group than in the control group, with this figure and pattern almost identical to the most comparable previous study.^12^ The baseline scores for the three primary outcomes (insomnia, paranoia, and hallucinations) were not associated with later missingness (appendix 1). Compared with participants who remained in the study, participants who dropped out from both groups were younger in age and more likely to be male (appendix 1). For the secondary measures, ISI, PHQ-9, Altman Mania Scale, and WSAS scores were slightly higher, and the WEMWBS score was slightly lower, in the missing groups than the non-missing groups (appendix 1).

Treatment uptake was relatively low. In the intervention group, 1302 participants (69%) logged on for at least one treatment session, 953 (50%) accessed at least two sessions, 672 (36%) accessed at least three sessions, 497 (26%) accessed at least four sessions, 390 (21%) accessed at least five sessions, and 331 (18%) accessed six sessions (figure).

Regarding the primary measures, the sleep treatment was associated with significant reductions, at all timepoints, in insomnia, paranoia, and hallucinations compared with the control group (all p<0·0001; table 2). The reduction in insomnia after treatment was large, while the reduction in psychotic experiences was small (table 2). After treatment, 454 (62%) of 733 individuals in the treatment group and 326 (29%) of 1142 individuals in the control group scored outside the clinical cutoff for insomnia used for trial entry. The treatment differences were robust to the three different types of sensitivity analyses for missing data (appendix 1). A conservative imputation (given the general improvement in scores in both groups) of missing data was used, whereby the last available measurement for a participant was imputed for all further missing measurements of that participant. All three primary outcome differences remained significant with last observation carried forward imputations (appendix 1). Treatment differences also remained consistent with the primary analysis when we repeated the main analyses covarying for baseline variables that predicted missingness for each outcome. We also used pattern mixture models. Treatment differences would still be significant assuming the missing individuals in the treatment group had outcome scores 2 points worse for insomnia and paranoia, and 1 point worse for hallucinations (predictably the hallucination scale scores were much lower than for the other two outcome variables; appendix 1).

**Table 2:** Primary outcome results

	**Insomnia (SCI-8)**				**Paranoia (GPTS)**				**Hallucinations (SPEQ)**			
	Unadjusted mean		Adjusted difference* (95% CI), d†	p value*	Unadjusted mean		Adjusted difference* (95% CI), d†	p value*	Unadjusted mean		Adjusted difference* (95% CI), d†	p value*
	Control	Treatment			Control	Treatment			Control	Treatment		
Week 3	12·34 (5·85)	14·96 (5·80)	2·62 (2·19 to 3·06), 0·61	<0·0001	24·63 (11·82)	22·61 (9·89)	−1·81 (−2·49 to −1·13), 0·15	<0·0001	5·06 (6·89)	4·06 (5·84)	−0·79 (−1·15 to −0·42), 0·12	<0·0001
Week 10	13·31 (6·45)	18·08 (6·66)	4·78 (4·29 to 5·26), 1·11	<0·0001	23·84 (12·16)	21·06 (9·08)	−2·22 (−2·98 to −1·45), 0·19	<0·0001	4·89 (7·24)	3·12 (5·12)	−1·58 (−1·98 to −1·18), 0·24	<0·0001
Week 22	14·43 (6·71)	19·27 (7·13)	4·81 (4·29 to 5·33), 1·12	<0·0001	23·84 (12·68)	20·75 (9·19)	−2·78 (−3·60 to −1·96), 0·24	<0·0001	4·71 (7·43)	2·87 (5·45)	−1·56 (−1·99 to −1·14), 0·23	<0·0001

Data are mean (SD). At week 3, 1398 participants were in the control group and 1044 participants were in the treatment group. At week 10, 1142 participants were in the control group and 733 participants were in the treatment group. At week 22, 971 participants were in the control group and 603 participants were in the treatment group. SCI-8=Sleep Condition Indicator 8-item version. GPTS=Green et al Paranoid Thought Scales. SPEQ=Specific Psychotic Experiences Questionnaire.

* Linear mixed effects model adjusted for gender, student status, week, and interaction of week with randomisation, and including a random effect for student within university. Covariance matrix of within subject measurements was unstructured.

† d is standardised effect size (Cohen's d).

For the mediation analyses, change in sleep over 3 weeks explained 30% of the intervention effect on paranoia at 10 weeks, with change in sleep over 10 weeks accounting for 58% of the treatment effect on paranoia (table 3). Change in sleep over 3 weeks explained 21% of the intervention effect on hallucinations at 10 weeks, with change in sleep over 10 weeks accounting for 39% of the intervention effect on hallucinations. Hence early changes in sleep explain approximately half of the total sleep-mediated changes in psychotic experiences by the end of treatment. In comparison, parallel analyses in the opposite direction indicated that changes in psychotic experiences explained a much smaller percentage of variation in improvements in sleep. Specifically, when paranoia and hallucination outcomes at 3 weeks were set as the mediators and the sleep outcome at 10 weeks as the main outcome, paranoia symptoms mediated just 3·8% of change in sleep and hallucinations mediated 3·4% of change in sleep. These outcomes lend further support to the causal pathway hypothesis proposed in this study.

**Table 3:** Mediation analysis* results

	**Total effect**		**Direct effect**		**Indirect effect**		**Percentage mediated**
	Effect size (95% CI)	p value	Effect size (95% CI)	p value	Effect size (95% CI)	p value	
**Paranoia (GPTS) outcome at week 10**							
Insomnia at week 3 (SCI-8)	−2·27 (−3·03 to −1·51)	<0·0001	−1·85 (−2·66 to 1·04)	<0·0001	−0·67 (−0·86 to −0·48)	<0·0001	29·5%
Insomnia at week 10 (SCI-8)	−2·27 (−3·03 to −1·51)	<0·0001	−0·97 (−1·80 to −0·14)	<0·0001	−1·31 (−1·60 to −1·02)	<0·0001	57·8%
**Hallucinations (SPEQ) outcome at week 10**							
Insomnia at week 3 (SCI-8)	−1·60 (−2·00 to −1·20)	<0·0001	−1·36 (−1·79 to −0·94)	<0·0001	−0·33 (−0·43 to −0·23)	<0·0001	20·7%
Insomnia at week 10 (SCI-8)	−1·60 (−2·00 to −1·20)	<0·0001	−0·90 (−1·34 to −0·46)	<0·0001	−0·62 (−0·78 to −0·46)	<0·0001	38·6%

Total n=1718. GPTS=Green et al Paranoid Thought Scales. SCI-8=Sleep Condition Indicator 8-item version. SPEQ=Specific Psychotic Experiences Questionnaire.

* Outcome and mediators modelled by means of linear mixed effects models and the total, direct, and indirect effects determined using the Baron and Kenny^31^ approach. The effect size is the adjusted treatment difference (ie, non-standardised treatment difference).

The large improvement in insomnia is confirmed with the ISI assessment (table 4). The sleep treatment also led to improvements in depression, and improvements in anxiety, prodromal symptoms, nightmares, psychological wellbeing, and functioning, and all these improvements were maintained over time (Table 4, Table 5). Those participants randomised to the sleep treatment were also less likely to meet criteria over the course of the trial for a depressive episode, anxiety disorder, or ultra-high risk of psychosis (table 5). However, contact with mental health services did not differ between groups (table 5). Furthermore, the sleep treatment led to a small, sustained increase in symptoms of mania (table 5). With the sleep treatment, a greater risk also existed of meeting criteria for a manic episode (table 5). No adverse events were reported to the trial team.

**Table 4:** Secondary outcome results

	**Unadjusted mean**		Adjusted difference*(95% CI), d†	**p value**
	Control‡	Treatment§		
**Insomnia (ISI)**				
Week 10	12·95 (5·27)	9·23 (5·18)	−3·72 (−4·16 to −3·29), 0·94	<0·0001
Week 22‡	12·17 (5·29)	8·62 (5·48)	−3·40 (−3·87 to −2·93), 0·86	<0·0001
**Nightmares (DDNSI)**				
Week 10	7·35 (7·85)	5·47 (6·91)	−1·63 (−2·16 to −1·10), 0·20	<0·0001
Week 22‡§	7·32 (7·93)	5·09 (6·66)	−1·84 (−2·41 to −1·26), 0·23	<0·0001
**Prodromal psychosis (PQ-16)**				
Week 10	4·35 (3·71)	3·37 (3·29)	−0·81 (−1·03 to −0·60), 0·24	<0·0001
Week 22	4·05 (3·83)	3·14 (3·24)	−0·74 (−0·98 to −0·51), 0·22	<0·0001
**Depression (PHQ-9)**				
Week 10	11·27 (6·72)	8·44 (6·16)	−2·83 (−3·30 to −2·35), 0·48	<0·0001
Week 22	10·34 (6·79)	8·00 (6·54)	−2·44 (−2·95 to −1·94), 0·42	<0·0001
**Anxiety (GAD-7)**				
Week 10	8·35 (6·06)	6·53 (5·40)	−1·86 (−2·29 to −1·43), 0·33	<0·0001
Week 22	7·67 (6·10)	6·14 (5·41)	−1·56 (−2·01 to −1·10), 0·28	<0·0001
**Mania (Altman)**				
Week 10	2·97 (3·03)	3·77 (3·33)	0·93 (0·67 to 1·19), −0·31	<0·0001
Week 22	2·92 (3·06)	3·57 (3·41)	0·75 (0·46 to 1·03), −0·25	<0·0001
**Functioning (WSAS)**				
Week 10	15·92 (8·89)	11·43 (8·37)	−4·36 (−5·03 to −3·69), 0·58	<0·0001
Week 22	14·92 (9·17)	10·25 (8·30)	−4·33 (−5·05 to −3·62), 0·57	<0·0001
**Wellbeing (WEMWBS)**				
Week 10	38·73 (9·78)	40·92 (9·63)	2·47 (1·72 to 3·22), 0·29	<0·0001
Week 22	39·63 (10·19)	42·12 (10·36)	2·78 (1·97 to 3·60), 0·32	<0·0001

Data are mean (SD). ISI=Insomnia Severity Index. DDNSI=Disturbing Dream and Nightmare Severity Index. PQ-16=Prodromal Questionnaire 16-item version. PHQ-9=Patient Health Questionnaire 9-item version. GAD-7=Generalised Anxiety Disorder 7-item version. WSAS=Work and Social Adjustment Scale. WEMWBS=Warwick–Edinburgh Mental Wellbeing Scale.

* Linear mixed effects model adjusted for gender, student status, week, and interaction of week with randomisation, and including a random effect for student within university. Covariance matrix of within subject measurements was unstructured.

† d is standardised effect size (Cohen's d).

‡ 1142 participants in the control group at week 10 and 971 participants at week 22, except for 970 participants for insomnia (ISI) and 963 participants for nightmares (DDNSI) at week 22.

§ 733 participants in the treatment group at week 10 and 603 participants at week 22, except for 599 participants for nightmares (DDNSI) at week 22.

**Table 5:** Secondary dichotomous outcome results

	Adjusted odds ratio*(95% CI)	**p value**
**Ultra-high risk of psychosis (PQ-16)**		
Week 10	0·26 (0·15 to 0·46)	<0·0001
Week 22	0·33 (0·18 to 0·59)	0·00026
**Mania (Altman)**		
Week 10	2·01 (1·48 to 2·73)	<0·0001
Week 22	1·89 (1·34 to 2·66)	0·00027
**Depressive disorder (PHQ-9)**		
Week 10	0·21 (0·14 to 0·32)	<0·0001
Week 22	0·32 (0·21 to 0·48)	<0·0001
**Anxiety disorder (GAD-7)**		
Week 10	0·32 (0·21 to 0·48)	<0·0001
Week 22	0·42 (0·27 to 0·64)	<0·0001
**Contacted mental health services**		
Week 10	1·19 (0·70 to 2·04)	0·52
Week 22	0·98 (0·55 to 1·75)	0·94
**Mental health diagnosis**		
Week 10	1·33 (0·75 to 2·37)	0·33
Week 22	1·43 (0·78 to 2·63)	0·25
**Psychiatric medication**		
Week 10	0·77 (0·47 to 1·26)	0·30
Week 22	0·96 (0·58 to 1·59)	0·86
**Psychological therapy**		
Week 10	1·27 (0·48 to 3·35)	0·63
Week 22	0·41 (0·11 to 1·58)	0·20

1142 participants in the control group at week 10 and 971 participants at week 22. 733 participants in the treatment group at week 10 and 603 participants at week 22. PQ-16=Prodromal Questionnaire 16-item version. PHQ-9=Patient Health Questionnaire 9-item version. GAD-7=Generalised Anxiety Disorder 7-item version.

* Logistic mixed effects model adjusted for gender, student status, week, and interaction of week with randomisation, and including a random effect for student within university. Covariance matrix of within subject measurements was unstructured.

## Discussion

We aimed to investigate the effects on mental health of the reduction of sleep difficulties. The first necessary stage was for the intervention to reduce insomnia, which was achieved. A large effect size reduction was found with the digital CBT intervention in a large student population. But we designed the trial to establish the consequent effects on psychotic experiences. To our knowledge, the OASIS trial is the largest randomised controlled trial of a psychological intervention for a mental health problem. Students randomly assigned to the sleep intervention showed small, sustained reductions in paranoia and hallucinations, suggesting that disrupted sleep has a contributory causal role in the occurrence of these psychotic experiences in a specific population of young adults. The mediation analyses supported this interpretation—eg, improvements in sleep accounted for almost 60% of the change in paranoia after treatment. Insomnia might not be the largest cause of psychotic experiences but it is not an epiphenomenon. Hence, this study adds to our understanding of the causes of psychotic experiences and might indicate a promising route into the early treatment of some psychotic problems.

The focus on a sleep intervention in a young adult population is important. Young people with incipient disorders might be very reluctant to seek help for psychiatric problems. Trouble sleeping is a common complaint with little stigma. Hence, it provides a much more acceptable focus for a first step in a care pathway. The digital sleep treatment gave added benefits. Depression in particular, but also anxiety, psychological wellbeing, nightmares, and perceived functioning all improved. The effects on anxiety and depression are consistent with the results of a meta-analysis.^33^ Participants who received the sleep treatment in the trial were less likely to report symptoms at a level that met criteria for ultra-high risk of psychosis, depression, or anxiety disorder. At baseline, the frequency of positive screens for psychosis risk with the Prodromal Questionnaire was high, similar to the risk found with this questionnaire for adolescents referred to treatment services;^34^ this high rate will reflect the well established associations of sleep difficulties with psychotic experiences^2^, ^3^, ^7^, ^8^ (ie, that participants have been selected for insomnia and therefore will score higher on psychosis measures), and also the limitations of brief self-report questionnaires for assessing psychosis risk. However, in the trial, sleep treatment did not affect contact with mental health services. Most participants were not in contact with these services so a longer follow-up period might be needed to truly test such effects. Furthermore, manic symptoms associated with the sleep treatment increased. This outcome might be due to an actual increase in problematic manic symptoms or it might simply reflect the overall increase in psychological wellbeing in the sample since the questionnaire domains concern cheerfulness, self-confidence, reduced need for sleep, increase in amount of activity, and talkativeness. The Altman scale has been found to correlate poorly with self-ratings of elation.^35^

Are the study results generalisable beyond a student population? We consider that the results are likely to apply to the wider adult population. We used a treatment developed for adults, which was not modified for students. The large treatment reduction in insomnia for the students is very similar to that found in trials with general adult populations,^10^, ^11^, ^36^ while previous studies^36^, ^37^ with community samples have shown self-help sleep treatment to reduce anxiety and depression. Nonetheless, only a direct comparison in a trial can definitively determine the generalisablilty of our findings. Although not the primary objective of the study, the trial does indicate that the provision of internet-delivered CBT for insomnia to university students is likely to lead to reductions overall in insomnia, and smaller reductions in a number of other mental health symptoms, with benefits for positive psychological wellbeing too. Tailoring of the intervention specifically for this population could enhance engagement and outcome effects. Support to complete the intervention might well be helpful too.

Several limitations exist in the study. First, the study relied on self-report questionnaires, albeit validated in their development against clinical interviews. Similar change captured in rater-assessed measures would have strengthened confidence in the study results. Second, the samples tested were predominately in the non-clinical range of psychotic experiences, restricting the conclusions to the less severe end of the psychosis spectrum. Third, the participants were self-selecting in responding to the invitation, which will have affected the representativeness of the sample. However, access to the study was via an Internet webpage, which is a simpler process than obtaining treatment from clinical services. The whole study could be completed in the privacy of the home, which means that far fewer barriers existed to participation than conventional patient trials. Fourth, the extent to which the results will generalise to the rest of the population is not known. Even within the student population, we do not know the representativeness of the participants. Fifth, bias in the outcome results will have been introduced because of the high dropout rate, especially in the treatment group, which is similar to comparable online studies.^12^ The results did remain robust against conservative assumptions in the sensitivity analyses about those participants who dropped out, but it is notable that treatment effects were greater for those participants who completed the sleep treatment. Finally, the causal argument rests on the plausible assumption that the sleep treatment first changes the occurrence of insomnia, since that was the topic of the intervention, but the mediation analyses in this trial based on week 10 outcomes cannot fully capture the temporal order of changes or rule out reverse causation. We were able to show a significant amount of mediation based on the week 3 insomnia score as a mediator, while evidence for reverse causation was weak, which does follow the predicted temporal causal pathway. In reality, it is difficult in a clinical trial to capture potential temporal changes between mediator and outcomes, since improvements in paranoia and hallucinations are likely to closely parallel the improvement in sleep.

This work can be taken forward in several possible ways. Determination of the mechanisms linking insomnia to psychotic experiences will shed further light on the causes of psychosis and potentially enable treatment improvement.^3^, ^13^ Of great clinical interest will be the evaluation of the effects of improving sleep for patients attending clinical services with ultra-high risk of psychosis, or established clinical psychotic experiences, or at the early stages of relapse. Our experience is that patients with psychosis value their sleep difficulties being appropriately addressed, that this enhances engagement with other treatments, and that better sleep can contribute to a reduction in psychotic experiences. Furthermore, a gap exists in mental health services regarding intervention for early, relatively non-specific presentations, and proper sleep treatment might provide a sensible first response. Overall, this trial indicates the importance of sleep difficulties for mental health in the general population and the need for a reconsideration in clinical services of the priority given to improving sleep.
