# Supplementary material for: The effects of improving sleep on mental health (OASIS): a randomised controlled trial with mediation analysis
Source: Lancet Psychiatry. 2017 Oct;4(10):749–58. doi: 10.1016/S2215-0366(17)30328-0 (PMC5614772; doi:10.1016/S2215-0366(17)30328-0)
Supplement: Supplementary appendix 1 [file mmc1.pdf]

THE LANCET
Psychiatry

Supplementary appendix 1

This appendix formed part of the original submission and has been peer reviewed. We post it as supplied by the authors.

Supplement to: Freeman D, Sheaves B, Goodwin GM, et al. The effects of improving sleep on mental health (OASIS): a randomised controlled trial with mediation analysis. *Lancet Psychiatry* 2017; published online Sept 6. http://dx.doi.org/10.1016/S2215-0366(17)30328-0.

Primary Care
Clinical Trials Unit

STATISTICAL ANALYSIS REPORT

OASIS

A parallel group, randomised controlled trial of digital cognitive behavioural therapy for insomnia versus treatment as usual: the impact of improved sleep on mental health in a student sample.

Version History

Version:	Version Date:	Changes:
v 0.1	20 September 2016	Original report by A Nickless
v 0.2	22 September 2016	Included ICC's for universities and counts for number of participants completing each number of sessions. Included mediation analysis results.
v 0.3	17 November 2016	Corrected typos, included references and corrected adjustment for baseline GPTS in GPTS primary analysis.
v 1.0	21 November 2016	Corrected typos, included description for WSAS outcome under impairment in functioning.

TABLE OF CONTENTS

TABLE OF CONTENTS **2**

LIST OF TABLES **3**

LIST OF FIGURES **4**

1 INTRODUCTION **5**

1.1 VALIDATION 5
1.2 SOFTWARE EMPLOYED 5

2 METHODS **6**

2.1 BACKGROUND INFORMATION 6
2.2 TRIAL/STUDY DESIGN 6
2.3 OBJECTIVES 6
2.4 TARGET POPULATION 7
2.5 INTERVENTIONS 7
2.6 OUTCOMES MEASURES 8
2.6.1 Primary outcome 8
2.6.2 Secondary outcomes 8
2.7 SAMPLE SIZE 10
2.8 RANDOMISATION AND BLINDING IN THE ANALYSIS STAGE 10
2.9 DATA CLEANING 11
2.10 ANALYSIS FOR DATA MONITORING COMMITTEE MEETINGS 11
2.11 DEFINITION OF POPULATION FOR ANALYSIS 11
2.12 DEVIATION FROM SAP 11

3 RESULTS **14**

3.1 REPRESENTATIVENESS OF STUDY SAMPLE AND PATIENT THROUGHPUT 14
3.2 RECRUITMENT 14
3.3 BASELINE CHARACTERISTICS OF PARTICIPANTS 14
3.4 NUMBER ANALYSED 16
3.5 PRIMARY ANALYSES 17
3.5.1 Primary outcome 17
3.6 SECONDARY ANALYSES 23
3.6.1 Primary outcome 23
3.6.2 Secondary outcomes 27
3.7 SENSITIVITY ANALYSES 34
3.7.1 Missing data mechanism 34
3.8 SAFETY ANALYSES 39

4 REFERENCES **40**

5 APPENDICES **41**

FINAL

LIST OF TABLES

Table 1 Table of summary statistics of Baseline Covariates of those participants who completed and those who were lost to follow up for the primary outcome from the SCI Questionnaire, together with the probability of the study arm and each of the covariates predicting missingness from the logistic regression model 12

Table 2 Table of baseline characteristics 14

Table 3 Adjusted and unadjusted results for the primary outcome SCI8 (Sleep Condition Indicator) at 10 weeks 18

Table 4 Adjusted and unadjusted results for the primary outcome GPTS (Green Paranoid Thoughts Scale) at 10 weeks 19

Table 5 Adjusted and unadjusted results for the primary outcome SPEQ (Specific Psychotic Experiences Questionnaire) at 10 weeks 21

Table 6 Mediation analysis results 22

Table 7 Summary statistics of SCI-8 measured at 3 weeks, 10 weeks and 22 weeks and the results of the linear mixed effects model for the change in SCI score 23

Table 8 Summary statistics by sessions attended (mean (standard deviation) N) 24

Table 9 Between-group difference in mean change in SCI-8 from baseline... 24

Table 10 Summary statistics of GPTS measured at 3 weeks, 10 weeks and 22 weeks and the results of the linear mixed effects model for the change in GPTS score 24

Table 11 Summary statistics by sessions attended (mean (standard deviation) N) 25

Table 12 Between-group difference in mean change in GPTS from baseline 25

Table 13 Summary statistics of SPEQ measured at 3 weeks, 10 weeks and 22 weeks and the results of the linear mixed effects model for the change in SPEQ score 26

Table 14 Summary statistics by sessions attended (mean (standard deviation) N) 26

Table 15 Between-group difference in mean change in SPEQ from baseline 26

Table 16 Summary statistics of SCI-9 measured at 3 weeks, 10 weeks and 22 weeks and the results of the linear mixed effects model for the change in SCI9 score 27

Table 17 Summary statistics of ISI measured at 10 weeks and 22 weeks, and the results of the linear mixed effects model for the change in ISI score 28

Table 18 Summary statistics of DDNSI measured at 10 weeks and 22 weeks, and the results of the linear mixed effects model for the change in DDNSI score 28

Table 19 Summary statistics of PQ-16 measured at 10 weeks and 22 weeks, and the results of the linear mixed effects model for the change in PQ-16 score 29

Table 20 Summary statistics of PHQ-9 measured at 10 weeks and 22 weeks, and the results of the linear mixed effects model for the change in PHQ-9 score 29

Table 21 Summary statistics of PHQ-4 measured at 3 weeks, and the results of the linear mixed effects model for the change in PHQ-4 score 30

FINAL

Table 22 Summary statistics of GAD-7 measured at 10 weeks and 22 weeks, and the results of the linear mixed effets model for the change in GAD-7 score 30

Table 23 Summary statistics of Altman Mania Score measured at 3, 10 and 22 weeks, and the results of the linear mixed effects model for the change in Altman Mania Score 31

Table 24 Summary statistics of WSAS measured at 10 weeks and 22 weeks, and the results of the linear mixed effects model for the change in WSAS Score 31

Table 25 Summary statistics of WEMWBS measured at 10 weeks and 22 weeks, and the results of the linear mixed effects model for the change in WEMWBS score 32

Table 26 Odds ratio and adjusted odds ratio for Sleepio versus TAU of exceeding clinical threshold at 3 weeks, 10 weeks and 22 weeks 33

Table 27 Odds ratio and adjusted odds ration for sleepio versus TAU of exceeding clinical threshold at 10 and 22 weeks 33

Table 28 Linear mixed effects model results for the difference in the change in SCI-8 score between the Sleepio group and TAU group when age and ethnicity are include as covariates 35

Table 29 Linear mixed effects model results for the difference in the change in SCI-8 score between the sleepio group and TAU group when the missing data are imputed using last observation carried foreward 36

Table 30 Linear mixed effects model results for the difference in the change in GPTS score between the Sleepio group and TAU group when age and ethnicity are included as covariates 37

Table 31 Linear mixed effects model results for the difference in the change in GPTS score betwen the Sleepio group and TAU group when the missing data are imputed using last observation carried foreward 37

Table 32 Linear mixed effects model results for the difference in the change in SPEQ score between the Sleepio group and TAU group when age and ethnicity are included as covariates 39

Table 33 Linear mixed effects model results for the difference in the change in SPEQ score between the Sleepio group and TAU group when the missing data are impute using last observation carried foreward 39

LIST OF FIGURES

Figure 1 Histograms of the SCI8 outcome at 10 weeks and residuals from model fit 17

Figure 2 Histograms of the GPTS outcome at 10 weeks and residuals from model fit 19

Figure 3 Histograms of the SPEQ outcome at 10 weeks and residuals from model fit 20

Figure 4 Pattern mixture model results for the SCI-8 outcome at 10 weeks 35

Figure 5 Pattern mixture model results for the GPTS outcome at 10 weeks 36

Figure 6 Pattern mixture model results for the SPEQ outcome at 10 weeks 38

FINAL

1 Introduction

This document details the analysis set out in the statistical analysis plan for the Wellcome Trust funded randomised controlled trial to evaluate the use of digital cognitive behavioural therapy for insomnia (CBTi) versus treatment as usual (TAU). Subsequent analyses of a more exploratory nature will not be bound by the strategy set out in the statistical analysis plan, though they are expected to follow the broad principles laid down in the statistical analysis plan.

The analysis strategy will be available on request when the principal papers are submitted for publication in a journal. Suggestions for subsequent analyses by journal editors or referees, will be considered carefully, and carried out as far as possible in line with the principles of this analysis strategy; if reported, the source of the suggestion will be acknowledged.

This report is based on the statistical analysis **Statistical Analysis Plan - OASIS v1.0.pdf** dated **18 August 2016**. Any deviations from the statistical analysis plan will be described and justified in this report of the trial.

Trial/Study statistician(s):

Alecia Nickless, Department of Primary Care Health Sciences, University of Oxford
alecia.nickless@phc.ox.ac.uk, 01865 617875

Validation statistician(s):

Jill Mollison, Department of Primary Care Health Sciences, University of Oxford
jill.mollison@phc.ox.ac.uk

Chief Investigator:

Professor Daniel Freeman, Department of Psychiatry, University of Oxford
daniel.freeman@psych.ox.ac.uk, 01865 226490

Trial/Study Manager:

Bryony Sheaves, Department of Psychiatry, University of Oxford
Bryony.sheaves@psych.ox.ac.uk, 01865 226486

1.1 Validation

Validation of results presented in this report was conducted by Paramdeep Kaur, and validation of mediation analysis by Prof. Richard Emsley. All results/major endpoints/primary endpoint were validated by independent programming using Stata. Results from Stata output were checked for transcription errors. Further details of validation including validation programs are saved in P:\OCHNCTU\OASIS\5. Analysis \6.Validation – Paramdeep.

1.2 Software employed

Stata version 14.1 Revision 20 July 2016

2 METHODS

2.1 BACKGROUND INFORMATION

Insomnia is a common psychological disorder which may lead to other psychological disorders such as depression, anxiety and psychosis. A digital cognitive behavioural therapy may improve sleep and in turn lead to improved mental health.

2.2 TRIAL/STUDY DESIGN

OASIS is a single blinded individual patient randomised controlled trial. A sample of 3754 university students presenting with symptoms of insomnia will be recruited and randomised to receive either cognitive behavioural therapy for insomnia plus treatment as usual, or treatment as usual (1:1).

Date of start of recruitment:	5 March 2015
Number recruited:	3755
Date of end of recruitment:	17 February 2016
Target number of subjects:	Originally 2614, before amendment to increase size

Timing of trial procedures is provided in Appendix 1.

2.3 OBJECTIVES

Primary objectives

1. To assess whether delivering a digital cognitive behavioural therapy for the treatment of insomnia (CBTi) improves insomnia symptoms in a sample of university students by the end of treatment (10 weeks post-randomisation).
2. To assess whether web delivered CBTi results in a reduction in psychotic-like experiences (paranoia and hallucinations) by the end of treatment (10 weeks post-randomisation).
3. To assess whether changes in insomnia symptoms will mediate the changes in psychotic-like experiences by the end of treatment (10 weeks post-randomisation).

Secondary objectives

1. To determine whether web delivered CBTi improves levels of depression, anxiety, nightmares, and mania by the end of treatment (10 weeks post-randomisation).
2. To determine whether web-based CBTi improves psychological wellbeing by the end of the treatment (10 weeks post-randomisation).
3. To determine if the effects of CBTi on the primary and secondary outcomes will be maintained at the scheduled follow-up assessment (22 weeks post-randomisation).
4. To determine if CBTi will lead to the occurrence of fewer mental health disorders during the period of the trial, as assessed by screening tools at 22 weeks post-randomisation for ultra-high risk of psychosis, bipolar affective disorder, depression, and anxiety, and by treatment by mental health services.

2.4 TARGET POPULATION

Students from a UK university with indications of insomnia as indicated by a score of 16 or lower on the Sleep Condition Indicator (SCI)

Inclusion criteria

- Screening positive for probable insomnia, indicated by the sleep condition indicator
- Age ≥18
- Student from a UK university

Exclusion Criteria

- None

2.5 INTERVENTIONS

Control: Treatment as Usual

Active Treatment: The CBT for insomnia intervention is delivered predominately via the internet. The delivery is structured into six sessions, lasting an average of 20 minutes each. The course takes a minimum of six weeks to complete, with sessions unlocked weekly. Participants can move at a slower pace, for up to a maximum of 12 weeks. All participants have to at least start the programme online. Certain tools (such as sleep diaries and relaxation audios) can also be accessed using the web browser of any smartphone. All of the six core sessions, sleep diaries, relaxation audios, and the scheduling tool can also be accessed using an iOS app, but this is only an option for participants who have an iPhone®. The treatment content is based on CBT for insomnia manuals [36–38] and includes a behavioural component (sleep restriction, stimulus control, and relaxation), a cognitive component (paradoxical intention, cognitive restructuring, mindfulness, positive imagery, and putting the day to rest) and an educational component (psycho-education and sleep hygiene).

The programme is highly interactive, and content is presented by an animated virtual therapist. Participants make a time for the session and are prompted via email and/or SMS if they do not 'attend'. Participants complete daily sleep diary information throughout the intervention, which are used by the programme to provide tailored, personalised help. Participants receive an email and/or SMS reminder each morning to prompt them to fill in their sleep diary. In addition, participants complete a short questionnaire at the beginning of therapy to set treatment goals. Throughout the course of therapy, participants have access to a moderated online community and an online library of information about sleep. Participants can view their online case file, which includes four sections: a progress review, a reminder of strategies to try out between sessions, an agreed sleep schedule, and a list of further reading. The system provides online analytics, which can be used to monitor adherence by assessing how many sessions were completed and the number of weeks to complete the course. Data from the CBT programme show that 90 % of participants complete the course within 10 weeks; participants will have access to the intervention for up to 12 weeks. We note that treatment as usual will actually comprise of no intervention at all for the majority of participants, since they are not being recruited from clinical services.

2.6 Outcomes Measures

Outcome measures are assessed at baseline and follow up (3, 10 and 22 weeks post-randomisation) and all data is collected via the web based platform. Weeks 0-22 are the main trial, to which this statistical analysis plan refers. At week 23 post-randomisation all participants in the treatment as usual group will be offered digital CBTi for help with their sleep problems. Following completion of the CBTi programme, all participants will again be asked to complete an assessment. This will be at week 33 post-randomisation.

See Appendix III for a table of outcomes assessment schedule.

In addition to the formal assessments in Appendix I the CBTi (Sleepio.com) system provides online analytics. These can be used for example to monitor adherence by assessing how many sessions were completed and the number of weeks to complete the full CBTi course. These will be used in exploratory analyses.

2.6.1 Primary Outcome

The primary outcome to assess for improvements in insomnia is the Sleep Condition Indicator (SCI) at 10 weeks (weeks 3 and 22 are secondary). The SCI total score is calculated by adding together the scores for the eight items. Each item ranges between 0 and 4, and the total score can range between 0 and 32. Higher scores indicate better sleep (Espie et al. 2014).

The primary outcomes to assess for a reduction in psychotic-like experiences are the Green Paranoid Thoughts Scale (GPTS) to assess paranoia and the Specific Psychotic Experiences Questionnaire (SPEQ) – Hallucinations to assess hallucinations at 10 weeks (weeks 3 and 22 are secondary). The GPTS assessment measures two dimensions of paranoid thinking: ideas about social reference and ideas about social persecution. Each dimension consists of 16 statements which are then rated according how true the subject believes the statement to be on a Linkert scale from 1 (don't believe at all) to 5 (totally believe). The total score for each dimension is obtained by summing all 16 responses, ranging from 16 to 80, with higher scores reflecting higher levels of paranoia (Green et al. 2008). Only Part B is completed by participants. We will use part B of this assessment on social persecution as the primary outcome measuring levels of paranoia.

The SPEQ assessment considers six different types of psychotic experiences: paranoia, hallucinations, cognitive disorganisation, grandiosity, anhedonia (all by means of self-report), and negative symptoms (via parent report). Only the SPEQ hallucination subscale is given to participants. The subscale for hallucination will be used as a primary outcome for hallucinatory experiences (which is one of two psychotic experiences to be tested in the primary analysis). This subscale consists of nine items. These items are measured on a 6-point scale (not at all (0), once per fortnight (1), once per week (2), several times per week (3), daily (4), more than once per day (5)) and the overall score, calculated by summing the nine responses, ranges from 0 to 45 (Ronald et al. 2014).

2.6.2 Secondary Outcomes

Listed below are the secondary outcomes, along with the objective to which they relate. Assessment points are at week 0, 3, 10 and 22 post-randomisation, but the 10 week outcome is of primary importance in all cases. Only the primary outcomes and the Altman mania scale are assessed at week 3. Further details of the questionnaires can be found in Appendix I.

- To determine whether web delivered CBTi improves sleep: reducing insomnia:

  - Insomnia Severity Index (ISI) - 7 questions, scored from 0 to 4. Scores are summed to obtain overall score which can range from 0 to 28, with higher values indicating increasing levels of insomnia. (0, 10, and 22 weeks post-randomisation)

  - The nine-item SCI (SCI-9)- includes one additional question regarding early morning waking. (0, 3, 10, and 22 weeks post-randomisation)

- To determine whether web delivered CBTi improves levels of depression:

  - Patient Heath Questionnaire (PHQ-9) - 9 questions, scored from 0 to 3. Scores are summed to obtain overall score which can range from 0 to 27, with higher values indicating increasing levels of depression. (0, 10, and 22 weeks post-randomisation)

- To determine whether web delivered CBTi improves levels of anxiety:

  - Generalised Anxiety Disorder questionnaire (GAD-7) - 7 questions, scored from 0 to 3. Scores are summed to obtain overall score which can range from 0 to 21, with higher values indicating increasing levels of anxiety. (0, 10, and 22 weeks post-randomisation)

- To determine whether web delivered CBTi reduces the severity of nightmares:

  - Disturbing dream and nightmare severity index (DDNSI) – 5 questions. It measures the number of nights with nightmares per week (0-7 nights) and number of total nightmares per week. The DDNSI also measures the severity and intensity of the nightmares on a Likert-type scale ranging from no problem (0) to extremely severe problem (6), as well as how often nightmares result in awakenings ranging from never/rarely (0) to always (4). The index score is calculated by adding the number of nightmares per week (up to 14), number of nights with nightmares per week, and ratings of the severity of the nightmares, the intensity of the nightmares, and the frequency of nightmare-related awakenings. The score can range from 0 to 37, with higher values indicating a higher risk of a clinically salient nightmare complaint.

- To determine whether web-based CBTi results in reduction of mania-like symptoms:

  - Altman mania scale - 5 questions, scored from 0 to 4. Scores are summed to obtain overall score which can range from 0 to 20, with higher values indicating increasing probability of a manic or hypomanic condition.

- To determine whether web-based CBTi improves psychological wellbeing by the end of the treatment:

  - Warwick-Edinburgh mental wellbeing scale (WEMWBS) – 14 items. Each item is rated from 1 (None of the time) to 5 (All the time). The 14 items are summed to give an overall score, which can range from 14 to 70, with higher scores indicating better wellbeing.

  - Work and Social Adjustment Scale (WSAS) – 5 items. The WSAS assess participants' perceived impairment in functioning, with higher scores indicating greater perceived impairment. Each questions is rated on a 0 to 8 scale: 0 indicates no impairment at all and 8 indicates very severe impairment. The maximum score of the WSAS is 40 (lower scores are better).

FINAL

- The outcomes of the following assessments will be dichotomised and used to assess whether improved sleep will decrease the likelihood of developing later psychiatric problems (assessed at 22 weeks): psychosis, bipolar affective disorder, depression and anxiety:
    - Prodromal questionnaire (PQ-16) - 16 questions, rated as true (present, 1) or false (absent, 0). Scores are summed to obtain overall score which can range from 0 to 16, with higher values indicating increasing risk of psychosis. (Dichotomisation limit = ≥ 6)
    - GAD-7 (Dichotomisation limit = ≥ 10)
    - PHQ-9 (Dichotomisation limit = ≥ 10)
    - Altman mania scale (Dichotomisation limit = ≥ 6)
- To determine if CBTi will lead to the less utilisation of mental health services, from the self-report of treatment by mental health services:
    - Current contact with mental health services
    - Current diagnosis
    - Current prescribed medications
    - Current receipt of psychological therapy

2.7 Sample Size

There are two primary outcomes: sleep (as measured by the SCI questionnaire) and psychotic like experiences (paranoia and hallucinations) (as measured by the GPTS and SPEQ). The sleep primary outcome would expect to find a larger standardised mean difference than psychotic-like experiences; hence psychotic symptoms have been used to determine the sample size in order to provide a conservative power calculation.

According to the original protocol, a sample size of 2614 would be collected. This would provide 90% power to detect a standardised mean difference of 0.15 in psychotic-like experiences (primary outcome), whilst accounting for a high level of expected attrition (40%). In a study amendment the sample size was increased, as drop-out rates were proving higher. Therefore 3754 participants were recruited (1877 per treatment arm).

2.8 Randomisation and Blinding in the Analysis Stage

Once participants have completed the baseline assessment (week 0), they will be randomised to the sleep improvement programme (delivered by sleepio.com) in addition to TAU, or to TAU alone. The size of the two groups will be even. Randomisation will be completed via an automated system. The study will use simple randomisation with an allocation ratio of 1:1, as recommended for large trials (Hewitt and Torgerson 2006). Participants will be informed of the outcome of randomisation by receipt of an email.

The study is single blinded, as the participants are aware of which arm of the trial they are allocated to, but the researcher assessors are blinded to the study arm of the participant.

FINAL

2.9 Data cleaning

All questionnaire items were first checked to ensure that each score was valid. Composite scores were calculated as described for the different primary and secondary outcomes, once the individual items had been confirmed as valid inputs.

All complete case data was included in the analysis with treatment set as randomised. Missing data was considered in a sensitivity analysis.

2.10 Analysis for Data Monitoring Committee meetings

No analyses were conducted for DMC meetings.

2.11 Definition of population for analysis

The intention to treat (ITT) population consists participants who were randomised to a study arm. All the completed outcomes were analysed according to the study arm assigned, assuming missing data was missing at random (MAR). A pattern mixture model was applied to the data allowing informative missing parameters to express the magnitude of departure from Missing Completely at Random assumption.

Table 1 provides a breakdown of the patients who were lost to follow up in terms of their treatment and baseline covariates, as well as the p-value for the association of treatment and the baseline characteristics for predicting missingness from a logistic regression model. There is an association between missingness and the treatment to which the participant was allocated. There is also an association between missingness and most of the baseline covariates, including age, gender, ethnicity, student status, and the university the participant attended. Only the total UCAS (Universities and Colleges Admissions Service) points score was not associated with missingness.

2.12 Deviation from SAP

There were no deviations from the SAP.

FINAL

TABLE 1 TABLE OF SUMMARY STATISTICS OF BASELINE COVARIATES OF THOSE PARTICIPANTS WHO COMPLETED AND THOSE WHO WERE LOST TO FOLLOW UP FOR THE PRIMARY OUTCOME FROM THE SCI QUESTIONNAIRE, TOGETHER WITH THE PROBABILITY OF THE STUDY ARM AND EACH OF THE COVARIATES PREDICTING MISSINGNESS FROM THE LOGISTIC REGRESSION MODEL

Baseline Characteristics	*Predicting missingness (p-value)*	*TAU (N= 1864)*		*SLEEPIO (N= 1891)*	
	Study arm p < 0.0001	*Missing (N=722)*	*Not Missing (N=1142)*	*Missing (N=1158)*	*Not Missing (N=733)*
Age (Years)	p < 0.0001	23.8 (6.6)	25.1 (8.2)	24.0 (6.9)	26.1 (8.8)
Total UCAS points	0.7687	771.6 (551.3)	741.1 (494.1)	718.9 (481.0)	723.8 (414.3)
Gender	0.0080				
**Male**		230 (31.9%)	300 (26.3%)	326 (28.2%)	187 (25.5%)
**Female**		487 (67.4%)	828 (72.5%)	825 (71.2%)	536(73.1%)
**Other**		5 (0.7%)	14 (1.2%)	7 (0.6%)	10 (1.4%)
Student Status	p < 0.0001				
**Undergraduate student**		564 (78.1%)	788 (69.0%)	901 (77.8%)	488 (66.6%)
**Postgraduate student**		145 (20.1%)	335 (29.3%)	235 (20.3%)	238 (32.5%)
**Other**		13 (1.8%)	19 (1.7%)	22 (1.9%)	7 (1.0%)
Ethnicity	0.0626				
**White British**		468 (64.8%)	744 (65.1%)	789 (68.1%)	476 (64.9%)
**White Irish**		18 (2.5%)	14 (1.2%)	16 (1.4%)	11 (1.5%)
**White Traveller**		1 (0.1%)	0 (0%)	3 (0.3%)	0 (0%)
**White Other**		98 (13.6%)	185 (16.2%)	141 (12.2%)	117 (16%)
**Mixed – White/Caribbean**		2 (0.3%)	11 (1%)	8 (0.7%)	3 (0.4%)
**Mixed – White/African**		3 (0.4%)	6 (0.5%)	10 (0.9%)	3 (0.4%)
**Mixed – White/Asian**		10 (1.4%)	21 (1.8%)	16 (1.4%)	11 (1.5%)
**Mixed- Other**		16 (2.2%)	20 (1.8%)	15 (1.3%)	14 (1.9%)
**Asian Indian**		10 (1.4%)	16 (1.4%)	29 (2.5%)	14 (1.9%)
**Asian Pakistani**		10 (1.4%)	13 (1.1%)	13 (1.1%)	9 (1.2%)
**Asian Bangladeshi**		7 (1%)	2 (0.2%)	5 (0.4%)	2 (0.3%)
**Asian – Chinese**		36 (5%)	59 (5.2%)	48 (4.1%)	25 (3.4%)
**Asian – Other**		13 (1.8%)	12 (1.1%)	18 (1.6%)	14 (1.9%)
**Black – African**		6 (0.8%)	20 (1.8%)	14 (1.2%)	9 (1.2%)
**Black – Caribbean**		5 (0.7%)	5 (0.4%)	7 (0.6%)	10 (1.4%)
**Black – Other**		2 (0.3%)	0 (0%)	1 (0.1%)	2 (0.3%)
**Arab**		7 (1%)	5 (0.4%)	12 (1%)	2 (0.3%)
**Other**		10 (1.4%)	9 (0.8%)	13 (1.1%)	11 (1.5%)
University	p < 0.0001				
**Goldsmiths University**		44 (6.1%)	43 (3.8%)	68 (5.9%)	9 (1.2%)
**Lancaster University**		42 (5.8%)	75 (6.6%)	57 (4.9%)	45 (6.1%)
**Middlesex University**		25 (3.5%)	29 (2.5%)	37 (3.2%)	18 (2.5%)
**Plymouth University**		96 (13.3%)	88 (7.7%)	125 (10.8%)	33 (4.5%)
**Royal Holloway**		7 (1.0%)	17 (1.5%)	19 (1.6%)	10 (1.4%)
**University College London**		19 (2.6%)	27 (2.4%)	21 (1.8%)	20 (2.7%)
**University of Bristol**		44 (6.1%)	98 (8.6%)	91 (7.9%)	80 (10.9%)
**University of Cambridge**		4 (0.6%)	20 (1.8%)	11 (0.9%)	13 (1.8%)
**University of Central Lancashire**		14 (1.9%)	15 (1.3%)	25 (2.2%)	16 (2.2%)
**University of East Anglia**		6 (0.8%)	13 (1.1%)	11 (0.9%)	14 (1.9%)
**University of Exeter**		9 (1.2%)	14 (1.2%)	17 (1.5%)	6 (0.8%)
**University of Glasgow**		57 (7.9%)	103 (9.0%)	108 (9.3%)	80 (10.9%)
**University of Leicester**		27 (3.7%)	67 (5.9%)	61 (5.3%)	32 (4.4%)
**University of Liverpool**		63 (8.7%)	39 (3.4%)	88 (7.6%)	19 (2.6%)
**University of Manchester**		17 (2.4%)	33 (2.9%)	29 (2.5%)	33 (4.5%)
**University of Nottingham**		16 (2.2%)	43 (3.8%)	34 (2.9%)	18 (2.5%)
**University of Oxford**		44 (6.1%)	92 (8.1%)	67 (5.8%)	59 (8.0%)

Baseline Characteristics	*Predicting missingness (p-value)*	*TAU (N= 1864)*		*SLEEPIO (N= 1891)*	
	Study arm p < 0.0001	*Missing (N=722)*	*Not Missing (N=1142)*	*Missing (N=1158)*	*Not Missing (N=733)*
**University of Sheffield**		32 (4.4%)	68 (6.0%)	52 (4.5%)	52 (7.1%)
**University of South Wales**		19 (2.6%)	30 (2.6%)	36 (3.1%)	26 (3.5%)
**University of Strathclyde**		7 (1.0%)	16 (1.4%)	16 (1.4%)	9 (1.2%)
**University of Sussex**		12 (1.7%)	41 (3.6%)	22 (1.9%)	26 (3.5%)
**University of Swansea**		48 (6.6%)	36 (3.2%)	54 (4.7%)	40 (5.5%)
**University of West of Scotland**		22 (3.0%)	50 (4.4%)	36 (3.1%)	29 (4.0%)
**University of Worcester**		21 (2.9%)	39 (3.4%)	23 (2.0%)	20 (2.7%)
**Other**		27 (3.7%)	46 (4.0%)	50 (4.3%)	26 (3.5%)
**<u>Outcomes at Baseline</u>**					
**SCI-8**	0.1344	9.9 (4.4)	10.2 (4.3)	9.9 (4.3)	10.0 (4.2)
**GPTS**	0.0597	24.3 (10.9)	25.0 (12.1)	26.1 (12.4)	24.2 (10.9)
**SPEQ**	0.0934	5.4 (7.2)	5.3 (6.7)	5.5 (6.7)	4.9 (5.8)
**SCI-9**	0.1816	11.9 (5.0)	12.2 (4.8)	11.9 (4.8)	12.0 (4.8)
**ISI**	0.0009	15.7 (4.0)	15.1 (4.0)	15.5 (3.9)	15.2 (3.9)
**DDNSI**	0.1444	8.4 (8.4)	7.9 (8.1)	7.9 (7.9)	7.5 (7.7)
**PQ-16**	0.0530	4.9 (3.4)	4.8 (3.5)	4.9 (3.4)	4.6 (3.3)
**PHQ-9**	0.0001	13.1 (5.8)	12.5 (6.0)	13.2 (5.8)	12.4 (5.7)
**GAD7**	0.1270	8.9 (5.6)	9.0 (5.6)	9.6 (5.7)	9.1 (5.4)
**Altman**	0.0172	3.6 (3.0)	3.5 (3.0)	3.6 (3.1)	3.2 (2.8)
**WSAS**	0.0127	18.0 (7.6)	17.5 (7.6)	17.9 (7.6)	17.2 (7.5)
**WEMWBS**	0.0140	37.3 (8.8)	38.3 (8.7)	37.6 (8.8)	38.0 (8.0)
**SCI-8 Cut-off**	0.4395				
**Above**		44 (6.1%)	81 (7.1%)	84 (7.3%)	35 (4.8%)
**Below**		678 (93.9%)	1061 (92.9%))	1074 (92.7%)	698 (95.2%)
**PQ-16 Cut-off**	0.3613				
**Above**		272 (37.7%)	434 (38.0%)	451 (38.9%)	260 (35.5%)
**Below**		450 (62.3%)	708 (61.7%)	707 (61.1%)	473 (64.5%)
**PHQ-9 Cut-off**	0.0002				
**Above**		503 (69.7%)	735 (64.4%)	814 (70.3%)	472 (64.4%)
**Below**		219 (30.3%)	407 (35.6%)	344 (29.7%)	261 (35.6%)
**GAD-7 Cut-off**	0.0333				
**Above**		423 (58.6%)	660 (57.8%)	593 (51.2%)	418 (57.0%)
**Below**		299 (41.4%)	482 (42.2%)	565 (48.8%)	315 (43.0%)
**Altman Mania Score Cut-off**	0.0187				
**Above**		169 (23.4%)	253 (22.2%)	279 (24.1%)	134 (18.3%)
**Below**		553 (76.6%)	889 (77.8%)	879 (75.9%)	599 (81.7%)
**Contact Mental Health Services**	0.2897				
**Yes**		117 (16.2%)	211 (18.5%)	208 (18.0%)	138 (18.8%)
**No**		605 (83.8%)	931 (81.5%)	950 (82.0%)	595 (81.2%)
**Diagnosis**	0.3917				
**Yes**		216 (29.9%)	374 (32.7%)	388 (33.5%)	253 (34.5%)
**No**		506 (70.1%)	768 (67.3%)	770 (66.5%)	480 (65.5%)
**Medication**	0.5865				
**Yes**		159 (22.0%)	274 (24.0%)	281 (24.3%)	179 (24.4%)
**No**		563 (78.0%)	868 (76.0%)	877 (75.7%)	554 (75.6%)
**Psychological Therapy**	0.2294				
**Yes**		671 (92.9%))	1047 (91.7%)	1078 (93.1%)	678 (92.5%)
**No**		51 (7.1%)	95 (8.3%)	80 (6.9%)	55 (7.5%)

***Data are either frequency (%) or mean (standard deviation (sd)) as stated**

3 RESULTS

3.1 REPRESENTATIVENESS OF STUDY SAMPLE AND PATIENT THROUGHPUT

Appendix I provides the Consort Flow Diagram of the participants through the study. The missing values for different events (i.e. questionnaires) do not always correspond to exactly the same missing participants. But there is a core of participants who were not assessed throughout. There was a significant relationship between missingness and the study arm. The balance of the participants in terms of their baseline covariates is discussed in section 3.3.

3.2 RECRUITMENT

Recruitment started on the 5th of March 2015 and ended on the 17th of February 2016, after recruiting 3755 participants. A protocol amendment was submitted to increase the sample size from 2614 to 3754, after it was determined that the rate of loss to follow-up was higher than initially expected.

3.3 BASELINE CHARACTERISTICS OF PARTICIPANTS

Table 2 provides the baseline characteristics of the participants recruited into each of the therapy arms. Simple randomisation was used with an allocation ratio of 1:1, as recommended for large trials. The covariates age, gender, total UCAS points, student status, ethnicity and university are well balanced between the two study arms.

TABLE 2 TABLE OF BASELINE CHARACTERISTICS

Baseline Characteristics	***TAU (N= 1864)***	***SLEEPIO (N= 1891)***
Age (Years)	24.6 (7.6)	24.8 (7.7)
Total UCAS points	753.0 (517.3)	720.8 (456.3)
Gender		
**Male**	530 (28.4%)	513 (27.1%)
**Female**	1315 (70.6%)	1361 (72.0%)
**Other**	19 (1.0%)	17 (0.9%)
Student Status		
**Undergraduate student**	1352 (72.5%)	1389 (73.5%)
**Postgraduate student**	480 (25.8%)	473 (25.0%)
**Other**	32 (1.7%)	29 (1.5%)
Ethnicity		
**White British**	1212 (65.0%)	1265 (66.9%)
**White Irish**	32 (1.7%)	27 (1.4%)
**White Traveller**	1 (0.1%)	3 (0.2%)
**White Other**	283 (15.2%)	258 (13.6%)
**Mixed – White/Caribbean**	13 (0.7%)	11 (0.6%)
**Mixed – White/African**	9 (0.5%)	13 (0.7%)
**Mixed – White/Asian**	31 (1.7%)	27 (1.4%)
**Mixed- Other**	36 (1.9%)	29 (1.5%)
**Asian Indian**	26 (1.4%)	43 (2.3%)
**Asian Pakistani**	23 (1.2%)	22 (1.2%)
**Asian Bangladeshi**	9 (0.5%)	7 (0.4%)
**Asian – Chinese**	95 (5.1%)	73 (3.9%)
**Asian – Other**	25 (1.3%)	32 (1.7%)

Baseline Characteristics	*TAU (N= 1864)*	*SLEEPIO (N= 1891)*
**Black – African**	26 (1.4%)	23 (1.2%)
**Black – Caribbean**	10 (0.5%)	17 (0.9%)
**Black – Other**	2 (0.1%)	3 (0.2%)
**Arab**	12 (0.6%)	14 (0.7%)
**Other**	19 (1%)	24 (1.3%)
University		
**Goldsmiths University**	87 (4.7%)	77 (4.1%)
**Lancaster University**	117 (6.3%)	102 (5.4%)
**Middlesex University**	54 (2.9%)	55 (2.9%)
**Plymouth University**	184 (9.9%)	158 (8.4%)
**Royal Holloway**	24 (1.3%)	29 (1.5%)
**University College London**	46 (2.5%)	41 (2.2%)
**University of Bristol**	142 (7.6%)	171 (9.0%)
**University of Cambridge**	24 (1.3%)	24 (1.3%)
**University of Central Lancashire**	29 (1.6%)	41 (2.2%)
**University of East Anglia**	19 (1.0%)	25 (1.3%)
**University of Exeter**	23 (1.2%)	23 (1.2%)
**University of Glasgow**	160 (8.6%)	188 (9.9%)
**University of Leicester**	94 (5.0%)	93 (4.9%)
**University of Liverpool**	102 (5.5%)	107 (5.7%)
**University of Manchester**	50 (2.7%)	62 (3.3%)
**University of Nottingham**	59 (3.2%)	52 (2.7%)
**University of Oxford**	136 (7.3%)	126 (6.7%)
**University of Sheffield**	100 (5.4%)	104 (5.5%)
**University of South Wales**	49 (2.6%)	62 (3.3%)
**University of Strathclyde**	23 (1.2%)	25 (1.3%)
**University of Sussex**	53 (2.8%)	48 (2.5%)
**University of Swansea**	84 (4.5%)	94 (5.0%)
**University of West of Scotland**	72 (3.9%)	65 (3.4%)
**University of Worcester**	60 (3.2%)	43 (2.3%)
**Other**	73 (3.9%)	76 (4.0%)
**Outcomes at Baseline**		
**SCI-8**	10.1 (4.3)	9.9 (4.3)
**GPTS**	24.8 (11.6)	25.4 (11.9)
**SPEQ**	5.3 (6.9)	5.3 (6.4)
**SCI-9**	12.1 (4.9)	11.9 (4.8)
**ISI**	15.3 (4.0)	15.4 (3.9)
**DDNSI**	8.1 (8.2)	7.7 (7.8)
**PQ-16**	4.9 (3.4)	4.8 (3.3)
**PHQ-9**	12.7 (5.9)	12.9 (5.8)
**GAD-7**	9.0 (5.6)	9.4 (5.6)
**Altman**	3.5 (3.0)	3.5 (3.0)
**WSAS**	17.7 (7.6)	17.6 (7.6)
**WEMWBS**	37.9 (8.8)	37.8 (8.5)
**SCI-8 Cut-off**		
**Above**	125 (6.7%)	119 (6.3%)
**Below**	1739 (93.3%)	1772 (93.7%)
**PQ-16 Cut-off**		
**Above**	706 (37.9%)	711 (37.6%)
**Below**	1158 (62.1%)	1180 (62.4%)
**PHQ-9 Cut-off**		
**Above**	1238 (66.4%)	1286 (68.0%)
**Below**	626 (33.6%)	605 (32.0%)
**GAD-7 Cut-off**		

FINAL

Baseline Characteristics	*TAU (N= 1864)*	*SLEEPIO (N= 1891)*
**Above**	781 (41.9%)	880 (46.5%)
**Below**	1083 (58.1%)	1011 (53.5%)
**Altman Mania Score Cut-off**		
**Above**	422 (22.6%)	413 (21.8%)
**Below**	1442 (77.4%)	1478 (78.2%)
**Contact Mental Health Services**		
**Yes**	328 (17.6%)	346 (18.3%)
**No**	1536 (82.4%)	1545 (81.7%)
**Diagnosis**		
**Yes**	590 (31.7%)	641 (33.9%)
**No**	1274 (68.3%)	1250 (66.1%)
**Medication**		
**Yes**	433 (23.2%)	460 (24.3%)
**No**	1431 (76.8%)	1431 (75.7%)
**Psychological Therapy**		
**Yes**	146 (7.8%)	135 (7.1%)
**No**	1718 (92.2%)	1756 (92.9%)
***Data are either frequency (%) or mean (standard deviation) as indicated**		

3.4 NUMBER ANALYSED

Appendix I and Appendix II provides the total number of participants who completed each of the study events which were analysed in the primary and secondary analyses. The dominant reasons for participants being lost to follow up were participants not wishing to continue within the therapy arm (n = 722 (38.7%) for TAU and n = 1158 (61.2%) for Sleepio with regards to the primary outcome). As this is an online intervention, if participants wish to no longer participate, then they simply do not proceed with any further assessments.

Missing outcomes are associated with baseline measures of covariates: age, gender, university, and student status. In both randomised groups, the average age was lower in the missing group compared to the non-missing group. In both randomised groups, the proportion of males in the missing group was higher compared to the non-missing group. In both randomised groups, the proportion of undergraduates in the missing group was higher compared to the non-missing group. In the case of ethnicity and university, there are a large number of groups, and therefore due to the large sample size, and small imbalance in these groups would result in a significant association. In the case of ethnicity, the proportion of white participants is approximately 80% in each of the missing and non-missing groups, across both randomised groups.

Baseline levels of the measured primary and secondary outcomes were also tested for associations with missingness. None of the primary outcomes (SCI, GPTS, or SPEQ) were associated with missingness. The secondary outcome ISI was associate with missingness, where ISI was slightly higher across both randomised groups in the missing group. The missing group across both randomised groups also had significantly higher levels of PHQ9, Altman Mania Scores, and WSAS scores. The missing group across both randomised groups had significantly lower levels of WEMWBS scores.

FINAL

3.5 Primary Analyses

3.5.1 Primary outcome

3.5.1.1 SCI

The SCI primary outcome is a composite score which can range from 0 to 32, with higher values indicating better sleep, and is assumed to be normally distributed. Table 3 provides the summary statistics of SCI at 10 weeks. For the control group, the unadjusted mean score was 13.3 which is indicative of a probable insomnia disorder (Espie et al. 2014). The Sleepio group had an unadjusted mean score of 18.1 which is indicative of the no insomnia disorder. The estimated ICC for universities was 0.013.

Assumptions of Normality of the outcome and of the residuals were checked using graphical methods. The outcome was found to be sufficiently normally distributed to fit the linear mixed effects model. Residuals were normally distributed (Figure 1).

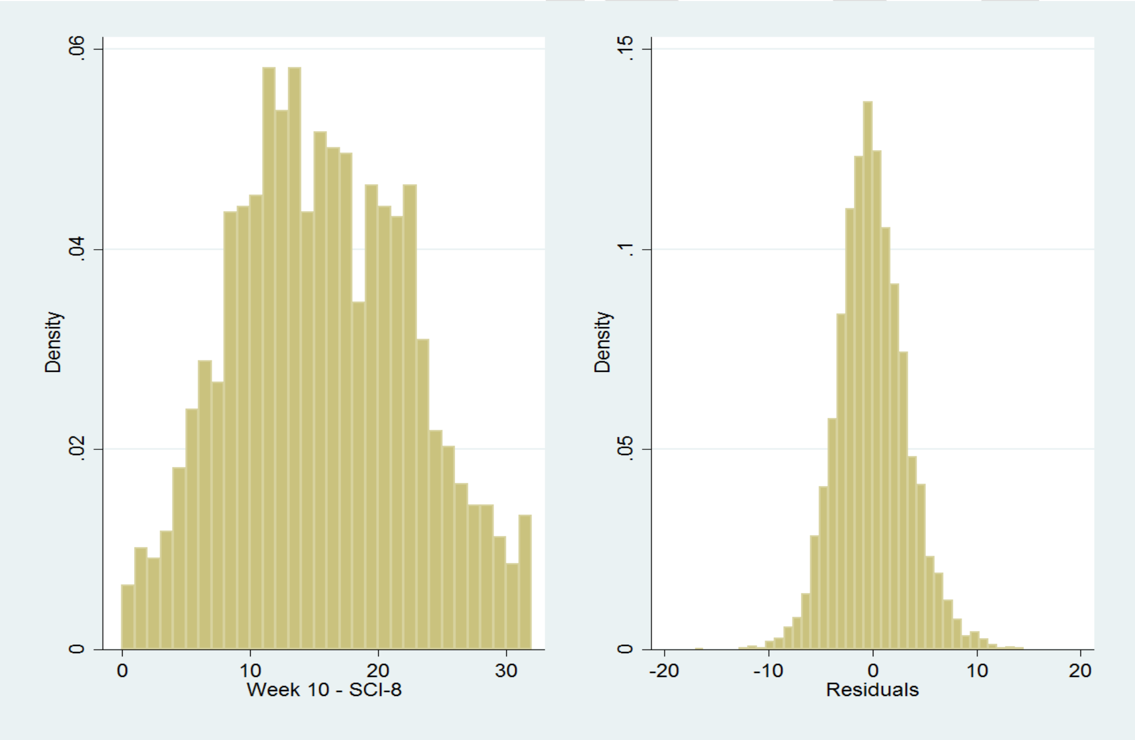

FIGURE 1 HISTOGRAMS OF THE SCI8 OUTCOME AT 10 WEEKS AND RESIDUALS FROM MODEL FIT

Appendix II provides the number of participants who completed the SCI outcome at different follow up periods. The risk difference for missingness between the two therapy arms is 0.23 with 95% confidence (0.19; 0.26), where the Sleepio group had a much higher risk of missingness.

The hypothesis that delivering a digital cognitive behavioural therapy for the treatment of insomnia (CBTi) improves insomnia symptoms in a sample of university students by the end of treatment was tested by means of a linear mixed effects model. There was a significant difference between treatment arms, with participants

in the Sleepio treatment showing significantly more improvement in their insomnia symptoms at the 10 week assessment time point (adjusted difference (95% C.I.): 4.78 (4.29; 5.26); p-value < 0.0001) (Table 3).

TABLE 3 ADJUSTED AND UNADJUSTED RESULTS FOR THE PRIMARY OUTCOME SCI8 (SLEEP CONDITION INDICATOR) AT 10 WEEKS

	Treatment	
	**TAU** N=1142	**Sleepio** N=733
**Unadjusted Mean (Standard Deviation)**	13.31 (6.45)	18.08 (6.66)
**Adjusted Difference in Treatment Effect (C.I.)***	4.78 (4.29; 5.26)	
**p-value**	<0.0001	
* Linear mixed effects model adjusted for gender, student status, week and interaction of week with randomisation, and including a random effect for student within university. Covariance matrix of within subject measurements unstructured.		

3.5.1.2 GPTS

The GPTS primary outcome is a composite score which can range from 16 to 80, with higher values indicating increasing levels of paranoia, and is assumed to be normally distributed. Table 3 provides the summary statistics of GPTS at 10 weeks. For the control group, the unadjusted mean score was 23.84 which is indicative of non-clinical sample. The Sleepio group had an unadjusted mean score of 21.06 which is also indicative of non-clinical sample. The estimated ICC for universities was <0.001.

The skewness of the outcome and assumption of normality of the residuals were checked using graphical methods. The outcome was found to be strongly skewed to the right, but the residuals were sufficiently normally distributed in order to use the linear mixed effects model for parameter estimates and confidence intervals (Figure 2).

FINAL

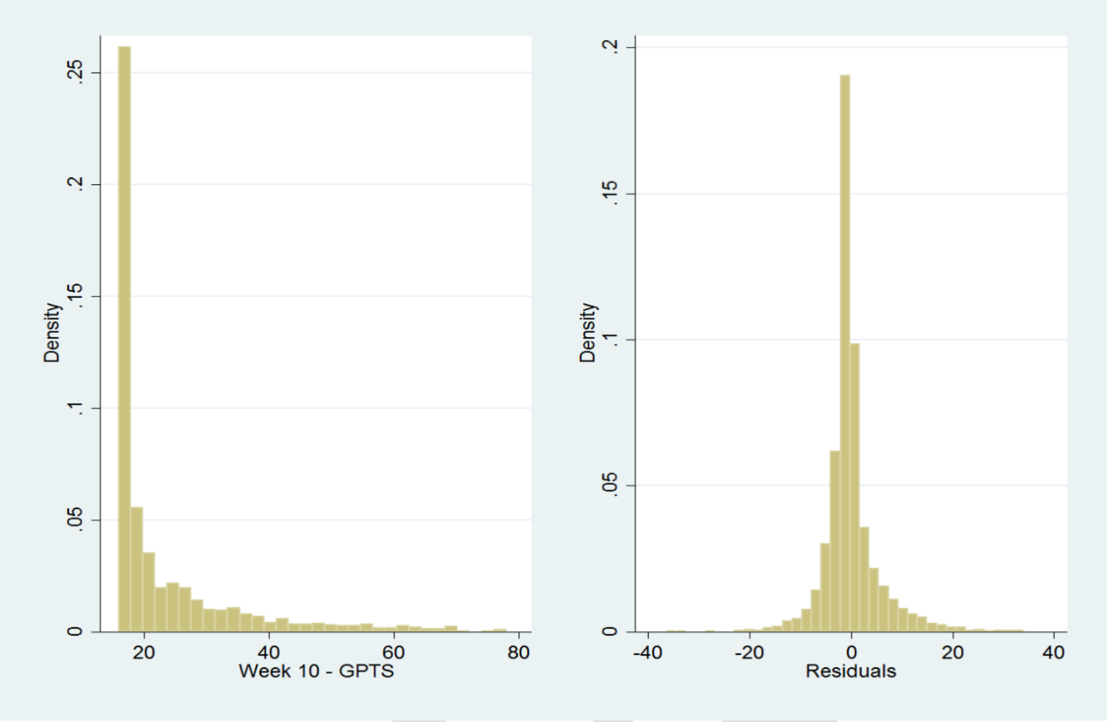

FIGURE 2 HISTOGRAMS OF THE GPTS OUTCOME AT 10 WEEKS AND RESIDUALS FROM MODEL FIT

Appendix II provides the number of participants who completed the GPTS outcome at different follow up periods. The risk difference for missingness between the two therapy arms is 0.23 with 95% confidence (0.19; 0.26), where the Sleepio group had a much higher risk of missingness.

The hypothesis that delivering a digital cognitive behavioural therapy for the treatment of insomnia (CBTi) in a sample of university students reduces psychotic-like experiences (paranoia and hallucinations) by the end of treatment was tested by means of a linear mixed effects model. There was a significant difference between treatment arms, with participants in the Sleepio treatment showing significantly reduced levels of paranoia at the 10 week assessment time point (adjusted difference (95% C.I.): -2.22 (-2.98; -1.45); p-value < 0.0001) (Table 4).

TABLE 4 ADJUSTED AND UNADJUSTED RESULTS FOR THE PRIMARY OUTCOME GPTS (GREEN PARANOID THOUGHTS SCALE) AT 10 WEEKS

	Treatment	
	**TAU**<br>N=1142	**Sleepio**<br>N=733
**Unadjusted Mean (Standard Deviation)**	23.84 (12.16)	21.06 (9.08)
**Adjusted Difference in Treatment Effect (C.I.)***	-2.22 (-2.98; -1.45)	
**p-value**	<0.0001	

*Linear mixed effects model adjusted for gender, student status, week and interaction of week with randomisation, and including a random effect for student within university. Covariance matrix of within subject measurements unstructured.

FINAL

3.5.1.3 SPEQ

The SPEQ hallucination subscale consists of nine items. These items are measured on a 6-point scale (not at all (0), once per fortnight (1), once per week (2), several times per week (3), daily (4), more than once per day (5)) and the overall score, calculated by summing the nine responses, ranges from 0 to 45. Higher scores indicate more hallucinations. The estimated ICC for universities was 0.005.

The skewness of the outcome and assumption of normality of the residuals were checked using graphical methods. The outcome was found to be strongly skewed to the right, but the residuals were sufficiently normally distributed in order to use the linear mixed effects model for parameter estimates and confidence intervals (Figure 3).

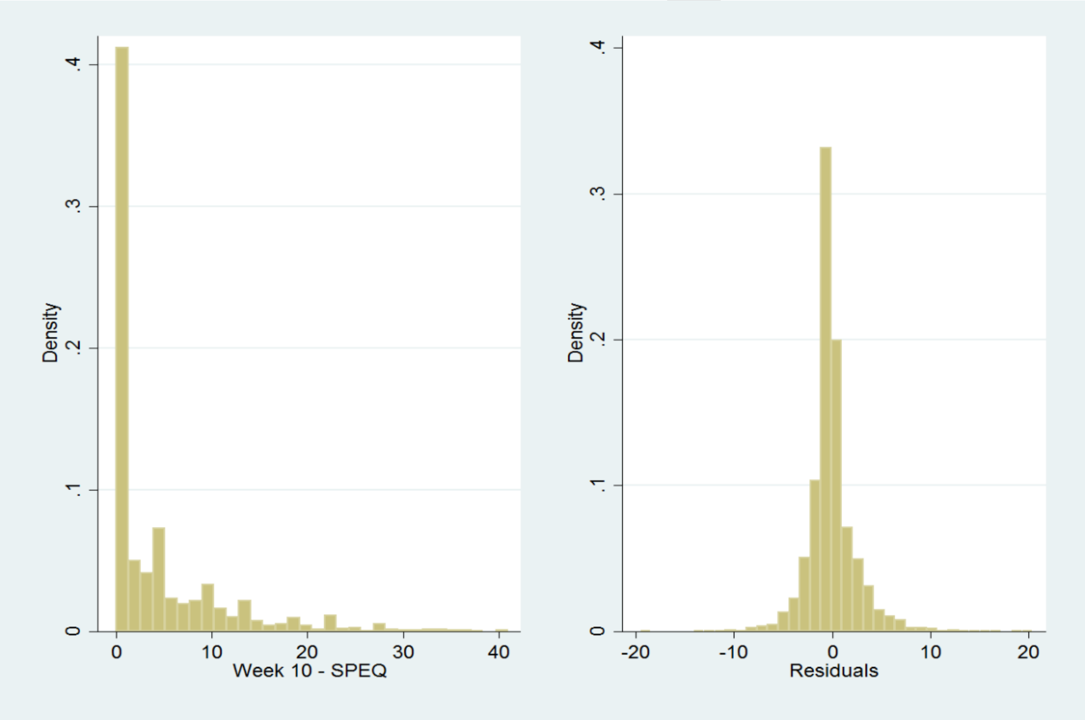

FIGURE 3 HISTOGRAMS OF THE SPEQ OUTCOME AT 10 WEEKS AND RESIDUALS FROM MODEL FIT

Appendix II provides the number of participants who completed the SPEQ outcome at different follow up periods. The risk difference for missingness between the two therapy arms is 0.23 with 95% confidence (0.19; 0.26), where the Sleepio group had a much higher risk of missingness.

The hypothesis that delivering a digital cognitive behavioural therapy for the treatment of insomnia (CBTi) in a sample of university students reduces psychotic-like experiences (paranoia and hallucinations) by the end of treatment was tested by means of a linear mixed effects model. There was a significant difference between treatment arms, with participants in the Sleepio treatment suffering significantly less from hallucinations at the 10 week assessment time point (adjusted difference (95% C.I.): -1.58 (-1.98; -1.18); p-value < 0.0001) (Table 4).

TABLE 5 ADJUSTED AND UNADJUSTED RESULTS FOR THE PRIMARY OUTCOME SPEQ (SPECIFIC PSYCHOTIC EXPERIENCES QUESTIONNAIRE) AT 10 WEEKS

	**Treatment**	
	**TAU** N=1142	**Sleepio** N=733
**Unadjusted Mean (Standard Deviation)**	4.89 (7.24)	3.12 (5.12)
**Adjusted Difference in Treatment Effect (C.I.)**	-1.58 (-1.98; -1.18)	
**p-value**	<0.0001	

* Linear mixed effects model adjusted for gender, student status, week and interaction of week with randomisation, and including a random effect for student within university. Covariance matrix of within subject measurements unstructured.

3.5.1.4 MEDIATION ANALYSIS

The third primary objective was to assess whether changes in insomnia symptoms mediates the changes in psychotic-like experiences by the end of treatment (10 weeks post-randomisation). To test this hypothesis we determined the extent of mediation of the 3 week SCI-8 outcome on the 10 week GPTS outcome and the extent of mediation of the 3 week SCI-8 outcome on the 10 week SPEQ outcome. We also considered 10 week SCI-8 outcome as the mediator, which requires the assumption that the effect of randomisation to the intervention is reflected in the SCI-8 outcome first before improvement is observed in the reduction of psychotic-like experiences. The approach used was similar to the approach of Baron and Kenny (1986), making use of linear mixed effects models at each step, similar to the linear mixed effects models used in the first two primary analyses. In all models baseline levels of the outcome and mediator were included as covariates. This is similar to the mediation analysis in the WIT study (Freeman et al 2015), but making using of linear mixed effects models to account for repeated measurements, rather than by means of structural equation modelling. The results of the mediation analysis are presented in Table 6.

When considering psychotic experiences (paranoia, hallucinations) as the outcome, the Sleepio intervention improved sleep at 3 weeks by a mean of 2.62 (SE (95% CI): 0.22 (2.19; 3.05); p-value < 0.0001) and reduced the paranoia score (GPTS) at 10 weeks by a mean of 2.27 (SE (95% CI): 0.39 (-3.03; -1.51); p-value < 0.0001).The intervention directly reduced the paranoia score at 10 weeks by 1.85 (SE (95% CI): 0.42 (-2.66; -1.04); p-value < 0.0001). Each unit improvement in the sleep score produced a -0.26 change in the paranoia score (SE (95% CI): 0.03 (-0.31; -0.20); p-value < 0.0001). The estimated indirect (mediated) effect of the intervention on the paranoia factor was a reduction of 0.67 (SE (95% CI): 0.10 (-0.86; -0.48); p-value < 0.0001). The proportion of the effect of the intervention on outcome (paranoia) that is mediated by changes in sleep is therefore 29.5%.

The Sleepio intervention improved sleep at 10 weeks by a mean of 4.77 (SE (95% CI): 0.25 (4.28; 5.25); p-value < 0.0001) and reduced the paranoia score (GPTS) at 10 weeks by a mean of 2.27 (SE (95% CI): 0.39 (-3.03; -1.51); p-value < 0.0001).The intervention directly reduced the paranoia score at 10 weeks by 0.97 (SE (95% CI): 0.42 (-1.80; -0.14); p-value = 0.0220). Each unit improvement in the sleep score produced a -0.28 change in the paranoia score (SE (95% CI): 0.03 (-0.33; -0.22); p-value < 0.0001). The estimated indirect (mediated) effect of the intervention on the paranoia factor was a reduction of 1.31 (SE (95% CI): 0.15 (-1.60; -1.02); p-value < 0.0001). The proportion of the effect of the intervention on outcome (paranoia) that is mediated by changes in sleep is therefore 57.8%.

When considering hallucinations as the outcome, the Sleepio intervention improved sleep at 3 weeks by a mean of 2.61 (SE (95% CI): 0.22 (2.18; 3.05); p-value < 0.0001) and reduced the hallucinations score (SPEQ) at 10 weeks by a mean of 1.60 (SE (95% CI): 0.20 (-2.00; -1.20); p-value < 0.0001). The intervention directly reduced the hallucinations score at 10 weeks by 1.36 (SE (95% CI): 0.22 (-1.79; -0.94); p-value < 0.0001). Each unit improvement in the sleep score produced a -0.13 change in the hallucinations score (SE (95% CI): 0.02 (-0.16; -0.09); p-value < 0.0001). The estimated indirect (mediated) effect of the intervention on the hallucination factor was a reduction of 0.33 (SE (95% CI): 0.05 (-0.43; -0.23); p-value < 0.0001). The proportion of the effect of the intervention on outcome (hallucinations) that is mediated by changes in sleep is therefore 20.7%.

The Sleepio intervention improved sleep at 10 weeks by a mean of 4.76 (SE (95% CI): 0.25 (4.27; 5.25); p-value < 0.0001) and reduced the hallucinations score (SPEQ) at 10 weeks by a mean of 1.60 (SE (95% CI): 0.20 (-2.00; -1.20); p-value < 0.0001). The intervention directly reduced the hallucinations score at 10 weeks by 0.90 (SE (95% CI): 0.23 (-1.34; -0.46); p-value < 0.0001). Each unit improvement in the sleep score at 10 weeks produced a -0.13 change in the hallucinations score (SE (95% CI): 0.02 (-0.16; -0.10); p-value < 0.0001). The estimated indirect (mediated) effect of the intervention on the hallucinations score was a reduction of 0.62 (SE (95% CI): 0.08 (-0.78; -0.46); p-value < 0.0001). The proportion of the effect of the intervention on outcome (hallucinations) that is mediated by changes in sleep is therefore 38.6%.

TABLE 6 MEDIATION ANALYSIS RESULTS

		**Total Effect**			**Direct Effect**			**Indirect Effect**			**Percent mediated**
**Outcome (week)**	**Mediator (week)**	**Effect size**	**SE**	**p**	**Effect size**	**SE**	**p**	**Effect size**	**SE**	**p**	
**Paranoia GPTS (10)**	**Insomnia SCI-8 (3)**	-2.27	0.39	<0.0001	-1.85	0.42	<0.0001	-0.67	0.10	<0.0001	29.5%
	**Insomnia SCI-8 (10)**	-2.27	0.39	<0.0001	-0.97	0.42	<0.0001	-1.31	0.15	<0.0001	57.8%
**Hallucinations SPEQ (10)**	**Insomnia SCI-8 (3)**	-1.60	0.20	<0.0001	-1.36	0.22	<0.0001	-0.33	0.05	<0.0001	20.7%
	**Insomnia SCI-8 (10)**	-1.60	0.20	<0.0001	-0.90	0.23	<0.0001	-0.62	0.08	<0.0001	38.6%

3.6 Secondary Analyses

3.6.1 Primary Outcome

3.6.1.1 Insomnia, SCI-8

Table 7 provides the results at 3 weeks, 10 weeks and 22 weeks for the SCI-8 score. In both arms the mean SCI-8 score increases, indicating improved sleep over time. The rate of increase in the SCI score is higher in the Sleepio treatment group. This is supported by significant positive differences between the treatment and control at 3 (adjusted difference (95% C.I.): 2.62 (2.19; 3.06); p-value < 0.0001), 10 (adjusted difference (95% C.I.): 4.78 (4.29; 5.26); p-value < 0.0001) and 22 weeks (adjusted difference (95% C.I.): 4.81 (4.29; 5.33); p-value < 0.0001). Sleep improvement is maintained through to 22 weeks, 12 weeks after the completion of treatment.

TABLE 7 SUMMARY STATISTICS OF SCI-8 MEASURED AT 3 WEEKS, 10 WEEKS AND 22 WEEKS AND THE RESULTS OF THE LINEAR MIXED EFFECTS MODEL FOR THE CHANGE IN SCI SCORE

	**SCI-8 3 Weeks**		**SCI-8 10 Weeks**		**SCI-8 22 Weeks**	
	TAU N=1398	Sleepio N=1044	TAU N=1142	Sleepio N=733	TAU N=971	Sleepio N=603
**Unadjusted Mean (Standard Deviation)**	12.34 (5.85)	14.96 (5.80)	13.31 (6.45)	18.08 (6.66)	14.43 (6.71)	19.27 (7.13)
**Adjusted Difference (C.I.)***	2.62 (2.19; 3.06)		4.78 (4.29; 5.26)		4.81 (4.29; 5.33)	
**p-value**	<0.0001		<0.0001		<0.0001	

* Linear mixed effects model adjusted for gender, student status, week and interaction of week with randomisation, and including a random effect for student within university. Covariance matrix of within subject measurements unstructured.

3.6.1.1.1 Compliance

Partial compliance to the intervention was assessed by the number of Sleepio sessions completed. The means and standard deviations for the SCI-8 score at each assessment are presented by the number of sessions completed (Table 8). The mean score increased in all groups from 3 to 10 to 22 weeks, and in the treatment group the score tended to increase as the number of sessions completed increased.

The complier-average causal effect was larger than the ITT, per protocol and as treated treatment effects, indicating that the improvement in SCI score was dependent on complying with the intervention (Table 9). Partial compliance was defined as attending at least one session.

TABLE 8 SUMMARY STATISTICS BY SESSIONS ATTENDED (MEAN (STANDARD DEVIATION) N)

	SCI-8 3 Weeks	SCI-8 10 Weeks	SCI-8 22 Weeks
**TAU (No sessions)**	12.34 (5.85) 1398	13.31 (6.45) 1142	14.43 (6.71) 971
**Sleepio Sessions**			
**0**	13.45 (6.22) 205	15.26 (6.57) 135	16.52 (7.06) 103
**1**	14.24 (5.55) 88	16.18 (6.65) 55	17.06 (7.81) 50
**2**	14.68 (5.68) 145	16.04 (6.57) 74	18.49 (6.76) 61
**3**	16.01 (5.80) 137	18.46 (5.67) 81	20.34 (6.69) 59
**4**	15.21 (6.35) 95	18.32 (7.48) 63	17.96 (7.43) 52
**5**	14.88 (5.46) 51	19.26 (6.79) 34	19.21 (6.43) 29
**6**	16.60 (10.14) 5	20.33 (11.50) 3	23.00 (12.73) 2
**7**	15.73 (5.26) 318	19.96 (6.08) 288	21.05 (6.71) 247

TABLE 9 BETWEEN-GROUP DIFFERENCE IN MEAN CHANGE IN SCI-8 FROM BASELINE

	SCI-8 3 Weeks	SCI-8 10 Weeks	SCI-8 22 Weeks
**ITT (C.I.)**	2.73 (2.34; 3.11)	4.92 (4.39; 5.45)	5.05 (4.42; 5.69)
**Per protocol**	3.12 (2.71; 3.53)	5.53 (4.97; 6.09)	5.62 (4.96; 6.29)
**As Treated**	2.98 (2.58; 3.38)	5.30 (4.74; 5.85)	5.40 (4.74; 6.07)
**CACE**	3.40 (2.92; 3.87)	6.03 (5.38; 6.68)	6.10 (5.34; 6.86)
Linear regression model adjusted for gender, and student status.			

3.6.1.2 PARANOIA, GPTS

Table 10 provides the results at 3 weeks, 10 weeks and 22 weeks for the GPTS score. In both arms the mean GPTS score decreases, indicating lower levels of paranoia. The rate of decrease in the GPTS score is faster in the Sleepio treatment group. This is supported by significant negative differences between the treatment and control at 3 (adjusted difference (95% C.I.): -1.81 (-2.49; -1.13); p-value < 0.0001), 10 (adjusted difference (95% C.I.): -2.22 (-2.98; -1.45); p-value < 0.0001) and 22 weeks (adjusted difference (95% C.I.): -2.78 (-3.60; -1.96); p-value < 0.0001). Improvement in levels of paranoia is maintained through to 22 weeks, 12 weeks after the completion of treatment.

TABLE 10 SUMMARY STATISTICS OF GPTS MEASURED AT 3 WEEKS, 10 WEEKS AND 22 WEEKS AND THE RESULTS OF THE LINEAR MIXED EFFECTS MODEL FOR THE CHANGE IN GPTS SCORE

	GPTS 3 Weeks		GPTS 10 Weeks		GPTS 22 Weeks	
	TAU N=1398	Sleepio N=1044	TAU N=1142	Sleepio N=733	TAU N=971	Sleepio N=603
**Unadjusted Mean (Standard Deviation)**	24.63 (11.82)	22.61 (9.89)	23.84 (12.16)	21.06 (9.08)	23.84 (12.68)	20.75 (9.19)
**Adjusted Difference (C.I.)***	-1.81 (-2.49; -1.13)		-2.22 (-2.98; -1.45)		-2.78 (-3.60; -1.96)	
**p-value**	<0.0001		<0.0001		<0.0001	
* Linear regression model adjusted for gender, student status, week and interaction of week with randomisation, and including a random effect for student within university. Covariance matrix of within subject measurements unstructured.						

FINAL

3.6.1.2.1 Compliance

Partial compliance to the intervention was assessed by the number of Sleepio sessions completed. The means and standard deviations for the GPTS score at each assessment are presented by the number of sessions completed (Table 11). The mean score decreased in all groups from 3 to 10 weeks, and decreased or stayed the same from 10 to 22 weeks, and in the treatment group the score tended to decrease as the number of sessions completed increased.

The complier-average causal effect was larger in magnitude than the ITT, per protocol and as treated treatment effects, indicating that the improvement in GPTS score was dependent on complying with the intervention (Table 12).

TABLE 11 SUMMARY STATISTICS BY SESSIONS ATTENDED (MEAN (STANDARD DEVIATION) N)

	**GPTS 3 Weeks**	**GPTS 10 Weeks**	**GPTS 22 Weeks**
**TAU (No sessions)**	24.63 (11.82) 1398	23.84 (12.16) 1142	23.84 (12.68) 971
**Sessions**			
**0**	23.14 (11.47) 205	22.93 (11.63) 135	22.12 (10.71) 103
**1**	23.48 (10.37) 88	22.44 (9.42) 55	21.28 (6.89) 50
**2**	23.34 (10.93) 145	21.42 (9.60) 74	21.39 (10.45) 61
**3**	22.30 (9.79) 137	20.36 (7.40) 81	20.14 (11.19) 59
**4**	23.07 (8.89) 95	22.71 (11.45) 63	22.29 (8.90) 52
**5**	22.25 (9.00) 51	20.68 (6.00) 34	19.83 (6.68) 29
**6**	17.80 (1.30) 5	18.33 (4.04) 3	19.00 (4.24) 2
**7**	21.83 (8.60) 318	19.75 (7.39) 288	19.87 (8.33) 247

TABLE 12 BETWEEN-GROUP DIFFERENCE IN MEAN CHANGE IN GPTS FROM BASELINE

	**GPTS 3 Weeks**	**GPTS 10 Weeks**	**GPTS 22 Weeks**
**ITT (C.I.)**	-1.89 (-2.53; -1.25)	-2.26 (-3.05; -1.46)	-2.82 (-3.78; -1.86)
**Per protocol**	-2.04 (-2.73; -1.35)	-2.61 (-3.46; -1.75)	-3.21 (-4.24; -2.19)
**As Treated**	-1.88 (-2.55; -1.21)	-2.53 (-3.36; -1.69)	-3.12 (-4.12; -2.12)
**CACE**	-2.35 (-3.15; -1.56)	-2.77 (-3.74; -1.79)	-3.41 (-4.56; -2.25)
Linear regression model adjusted for gender, and student status.			

3.6.1.3 Hallucinations, SPEQ

Table 13 provides the results at 3 weeks, 10 weeks and 22 weeks for the SPEQ score. In both arms the mean SPEQ score decreases, indicating lower levels of hallucinations. The rate of decrease in the SPEQ score is faster in the Sleepio treatment group. This is supported by significant negative differences between the treatment and control at 3 (adjusted difference (95% C.I.): -0.79 (-1.15; -0.42); p-value < 0.0001), 10 (adjusted difference (95% C.I.): -1.58 (-1.98; -1.18); p-value < 0.0001) and 22 weeks (adjusted difference (95% C.I.): -1.56 (-1.99; -1.14); p-value < 0.0001). Improvement in levels of paranoia is maintained through to 22 weeks, 12 weeks after the completion of treatment.

TABLE 13 SUMMARY STATISTICS OF SPEQ MEASURED AT 3 WEEKS, 10 WEEKS AND 22 WEEKS AND THE RESULTS OF THE LINEAR MIXED EFFECTS MODEL FOR THE CHANGE IN SPEQ SCORE

	SPEQ 3 Weeks		SPEQ 10 Weeks		SPEQ 22 Weeks	
	TAU N=1398	Sleepio N=1044	TAU N=1142	Sleepio N=733	TAU N=971	Sleepio N=603
**Unadjusted Mean (Standard Deviation)**	5.06 (6.89)	4.06 (5.84)	4.89 (7.24)	3.12 (5.12)	4.71 (7.43)	2.87 (5.45)
**Adjusted Difference (C.I.)***	-0.79 (-1.15; -0.42)		-1.58 (-1.98; -1.18)		-1.56 (-1.99; -1.14)	
**p-value**	<0.0001		<0.0001		<0.0001	

*Linear mixed effects model adjusting for infant age and gender, infant temperament, PND severity, and socioeconomic status, with fixed effects for month and therapy arm, and random effects for participants.

3.6.1.3.1 Compliance

Partial compliance to the intervention was assessed by the number of Sleepio sessions completed. The means and standard deviations for the SPEQ score at each assessment are presented by the number of sessions completed (Table 14). The mean score decreased in all groups from 3 to 10 weeks, and decreased or stayed the same from 10 to 22 weeks, and in the treatment group the score tended to decrease as the number of sessions completed increased.

The complier-average causal effect was larger in magnitude than the ITT, per protocol and as treated treatment effects, indicating that the improvement in SPEQ score was dependent on complying with the intervention (Table 15).

TABLE 14 SUMMARY STATISTICS BY SESSIONS ATTENDED (MEAN (STANDARD DEVIATION) N)

	SPEQ 3 Weeks	SPEQ 10 Weeks	SPEQ 22 Weeks
**TAU (No sessions)**	5.06 (6.89) 1398	4.89 (7.24) 1142	4.71 (7.43) 971
**Sessions**			
**0**	4.64 (6.76) 205	3.17 (5.07) 135	2.98 (4.68) 103
**1**	3.80 (5.32) 88	3.40 (6.07) 55	2.56 (4.44) 50
**2**	3.91 (4.96) 145	3.76 (5.99) 74	3.30 (7.12) 61
**3**	4.83 (7.33) 137	3.28 (4.92) 81	3.98 (7.00) 59
**4**	4.35 (4.83) 95	3.97 (5.52) 63	2.79 (3.88) 52
**5**	4.33 (7.11) 51	2.94 (4.63) 34	4.55 (7.30) 29
**6**	2.00 (3.08) 5	0.67 (1.15) 3	2.50 (3.54) 2
**7**	3.39 (4.96) 318	2.69 (4.75) 288	2.33 (5.04) 247

TABLE 15 BETWEEN-GROUP DIFFERENCE IN MEAN CHANGE IN SPEQ FROM BASELINE

	SPEQ 3 Weeks	SPEQ 10 Weeks	SPEQ 22 Weeks
**ITT (C.I.)**	-0.87 (-1.20; -0.53)	-1.50 (-1.94; -1.06)	-1.60 (-2.10; -1.09)
**Per protocol**	-0.92 (-1.28; -0.57)	-1.43 (-1.89; -0.96)	-1.53 (-2.07; -0.98)
**As Treated**	-0.84 (-1.19; -0.49)	-1.26 (-1.72; -0.80)	-1.36 (-1.89; -0.83)
**CACE**	-1.08 (-1.50; -0.66)	-1.84 (-2.38; -1.30)	-1.93 (-2.54; -1.31)

Linear regression model adjusted for gender, and student status.

3.6.2 Secondary Outcomes

3.6.2.1 SCI-9

The SCI-9 score includes all the same questions as SCI-8, but includes one additional question regarding early morning waking, therefore it can range between 0 and 36, with higher scores indicating improved sleep. Table 16 provides the summary statistics of the SCI9 score at 3, 10 and 22 weeks. At 3 weeks both treatment arms showed improvement over the baseline measurements. The Sleepio participants had significantly more improved sleep condition compared to the control group (3.08 (2.61; 3.55); p-value < 0.0001). At 10 weeks both arms of the study show further improved SCI-9 mean scores compared to baseline levels, which indicates improvement in sleep at 10 week. The improvement is significantly higher in the Sleepio treatment group compared to the control group (adjusted difference (95% C.I.): 5.43 (4.90; 5.95); p-value < 0.0001). The improvement in sleep in maintained through to 22 weeks in both groups. At 22 weeks the Sleepio treatment group had significantly higher improved sleep condition compared to the control group (5.35 (4.79; 5.92); p-value < 0.0001).

TABLE 16 SUMMARY STATISTICS OF SCI-9 MEASURED AT 3 WEEKS, 10 WEEKS AND 22 WEEKS AND THE RESULTS OF THE LINEAR MIXED EFFECTS MODEL FOR THE CHANGE IN SCI9 SCORE

	**SCI9 3 Weeks**		**SCI9 10 Weeks**		**SCI9 22 Weeks**	
	TAU N=1398	Sleepio N=1044	TAU N=1142	Sleepio N=733	TAU N=971	Sleepio N=603
**Unadjusted Mean (Standard Deviation)**	14.40 (6.34)	17.54 (6.35)	15.41 (7.02)	20.85 (7.24)	16.39 (7.28)	21.85 (7.74)
**Adjusted Difference (C.I.)***	3.08 (2.61; 3.55)		5.43 (4.90; 5.95)		5.35 (4.79; 5.92)	
**p-value**	<0.0001		<0.0001		<0.0001	

* Linear mixed effects model adjusted for gender, student status, week and interaction of week with randomisation, and including a random effect for student within university. Covariance matrix of within subject measurements unstructured.

3.6.2.2 ISI

Insomnia Severity Index (ISI) is made up of 7 questions, scored from 0 to 4. Scores are summed to obtain overall score which can range from 0 to 28, with higher values indicating increasing levels of insomnia. Table 17 provides the summary statistics of the ISI score at 10 and 22 weeks. At 10 weeks both arms of the study show reduced mean scores for ISI compared to baseline levels, which indicates improvement in sleep at 10 week. The reduction is significantly higher in the Sleepio treatment group compared to the control group (adjusted difference (95% C.I.): -3.72 (-4.16; -3.29); p-value < 0.0001). The improvement in sleep is maintained through to 22 weeks, in both groups. At 22 weeks the Sleepio treatment group had significantly more improved sleep compared to the control group (-3.40 (-3.87; -2.93); p-value < 0.0001).

FINAL

TABLE 17 SUMMARY STATISTICS OF ISI MEASURED AT 10 WEEKS AND 22 WEEKS, AND THE RESULTS OF THE LINEAR MIXED EFFECTS MODEL FOR THE CHANGE IN ISI SCORE

	**ISI 10 Weeks**		**ISI 22 Weeks**	
	TAU N=1142	Sleepio N=733	TAU N=970	Sleepio N=603
**Unadjusted Mean (Standard Deviation)**	12.95 (5.27)	9.23 (5.18)	12.17 (5.29)	8.62 (5.48)
**Adjusted Difference (C.I.)***	-3.72 (-4.16; -3.29)		-3.40 (-3.87; -2.93)	
**p-value**	<0.0001		<0.0001	

* Linear mixed effects model adjusted for gender, student status, week and interaction of week with randomisation, and including a random effect for student within university. Covariance matrix of within subject measurements unstructured.

3.6.2.3 Nightmares, DDNSI

Disturbing dream and nightmare severity index (DDNSI) consists of 5 questions. The score can range from 0 to 37, with higher values indicating a higher risk of a clinically salient nightmare complaint. At 10 weeks the control group showed only slightly improvement over the baseline levels in the DDNSI score (Table 18). The Sleepio had a much larger reduction in the DDNSI score, which was significantly different from the reduction achieved by the control group (adjusted difference (95% C.I.): -1.63 (-2.16; -1.10); p-value < 0.0001). At 22 weeks the control group showed no further improvement in the DDNSI score. The Sleepio group showed slightly further improved DDNSI scores, indicating less severe disturbing dreams and nightmares than the control group, and the change in DDNSI score was significantly different from that of the control group (adjusted difference (95% C.I.): -1.84 (-2.41; -1.26); p-value < 0.0001).

TABLE 18 SUMMARY STATISTICS OF DDNSI MEASURED AT 10 WEEKS AND 22 WEEKS, AND THE RESULTS OF THE LINEAR MIXED EFFECTS MODEL FOR THE CHANGE IN DDNSI SCORE

	**DDNSI 10 Weeks**		**DDNSI 22 Weeks**	
	TAU N=1142	Sleepio N=733	TAU N=963	Sleepio N=599
**Unadjusted Mean (Standard Deviation)**	7.35 (7.85)	5.47 (6.91)	7.32 (7.93)	5.09 (6.66)
**Adjusted Difference (C.I.)***	-1.63 (-2.16; -1.10)		-1.84 (-2.41; -1.26)	
**p-value**	<0.0001		<0.0001	

* Linear mixed effects model adjusted for gender, student status, week and interaction of week with randomisation, and including a random effect for student within university. Covariance matrix of within subject measurements unstructured.

3.6.2.4 Risk of psychosis, PQ-16

Prodromal questionnaire (PQ-16) consists of 16 questions, rated as true (present, 1) or false (absent, 0). Presence and absence scores are summed to obtain overall score which can range from 0 to 16, with higher values indicating increasing risk of psychosis. Table 19 provides the summary statistics and results from the linear mixed effects model. The improvement in levels of psychosis were small in the control group relative to the baseline levels. The improvement was larger in the Sleepio treatment group. At 10 weeks the Sleepio had significantly larger mean reduction in PQ-16 values compared to the control group (adjusted difference (95% C.I.): -0.81 (-1.03; -0.60); p-value < 0.0001). At 22 weeks, the Sleepio group still had significantly larger

reduction in mean PQ-16 score compared to the control group (adjusted difference (95% C.I.): -0.74 (-0.98; -0.51); p-value < 0.0001).

TABLE 19 SUMMARY STATISTICS OF PQ-16 MEASURED AT 10 WEEKS AND 22 WEEKS, AND THE RESULTS OF THE LINEAR MIXED EFFECTS MODEL FOR THE CHANGE IN PQ-16 SCORE

	**PQ-16 10 Weeks**		**PQ-16 22 Weeks**	
	TAU N=1142	Sleepio N=733	TAU N=971	Sleepio N=603
**Unadjusted Mean (Standard Deviation)**	4.35 (3.71)	3.37 (3.29)	4.05 (3.83)	3.14 (3.24)
**Adjusted Difference (C.I.)***	-0.81 (-1.03; -0.60)		-0.74 (-0.98; -0.51)	
**p-value**	<0.0001		<0.0001	

* Linear mixed effects model adjusted for gender, student status, week and interaction of week with randomisation, and including a random effect for student within university. Covariance matrix of within subject measurements unstructured.

3.6.2.5 Depression, PHQ-9

The Patient Health Questionnaire (PHQ-9) questionnaire includes 9 questions, scored from 0 to 3. Scores are summed to obtain an overall score which can range from 0 to 27, with higher values indicating increasing levels of depression. Table 20 provides the summary statistics and results from the linear mixed effects model. The improvement in feelings of depression are small in the control group relative to the baseline levels. The improvement was larger in the Sleepio treatment group. At 10 weeks the Sleepio had significantly larger mean reduction in PHQ-9 values compared to the control group (adjusted difference (95% C.I.): -2.83 (-3.30; -2.35); p-value < 0.0001). At 22 weeks, the Sleepio group still had significantly larger reduction in mean PHQ-9 score compared to the control group (adjusted difference (95% C.I.): -2.44 (-2.95; -1.94); p-value < 0.0001).

TABLE 20 SUMMARY STATISTICS OF PHQ-9 MEASURED AT 10 WEEKS AND 22 WEEKS, AND THE RESULTS OF THE LINEAR MIXED EFFECTS MODEL FOR THE CHANGE IN PHQ-9 SCORE

	**PHQ-9 10 Weeks**		**PHQ-9 22 Weeks**	
	TAU N=1142	Sleepio N=733	TAU N=971	Sleepio N=602
**Unadjusted Mean (Standard Deviation)**	11.27 (6.72)	8.44 (6.16)	10.34 (6.79)	8.00 (6.54)
**Adjusted Difference (C.I.)***	-2.83 (-3.30; -2.35)		-2.44 (-2.95; -1.94)	
**p-value**	<0.0001		<0.0001	

* Linear mixed effects model adjusted for gender, student status, week and interaction of week with randomisation, and including a random effect for student within university. Covariance matrix of within subject measurements unstructured.

3.6.2.6 PHQ-4

The Patient Health Questionnaire (PHQ-4) questionnaire includes 4 questions, scored from 0 to 3. Scores are summed to obtain an overall score which can range from 0 to 12, with higher values indicating increasing levels of depression. Table 21 provides the summary statistics and results from the linear mixed effects model. The mean PHQ-4 score for depression is significantly larger in the control group than in the Sleepio group at week 3 (adjusted difference (95% C.I.): -0.62 (-0.88; -0.36); p-value < 0.0001).

FINAL

TABLE 21 SUMMARY STATISTICS OF PHQ-4 MEASURED AT 3 WEEKS, AND THE RESULTS OF THE LINEAR MIXED EFFECTS MODEL FOR THE CHANGE IN PHQ-4 SCORE

	Treatment	
	**TAU** N=1398	**Sleepio** N=1044
**Unadjusted Mean (Standard Deviation)**	5.25 (3.35)	4.62 (3.16)
**Adjusted Difference in Treatment Effect (C.I.)**	-0.62 (-0.88; -0.36)	
**p-value**	<0.0001	
* Linear mixed effects model adjusted for gender, and student status, and including a random effect for university. Covariance matrix of within cluster measurements unstructured.		

3.6.2.7 ANXIETY, GAD-7

The Generalised Anxiety Disorder (GAD-7) questionnaire is made up of 7 questions, scored from 0 to 3. Scores are summed to obtain overall score which can range from 0 to 21, with higher values indicating increasing levels of anxiety. In both treatment groups the mean GAD-7 score is lower compared to baseline levels, but the improvement in the Sleepio group is more substantial, indicating lower levels of anxiety in the Sleepio group (Table 22). The mean change in GAD-7 score is significantly larger in the Sleepio group compared to the control group at week 10 (adjusted difference (95% C.I.): -1.86 (-2.29; -1.43); p-value < 0.0001) and at week 22 (adjusted difference (95% C.I.): -1.56 (-2.01; -1.10); p-value < 0.0001).

TABLE 22 SUMMARY STATISTICS OF GAD-7 MEASURED AT 10 WEEKS AND 22 WEEKS, AND THE RESULTS OF THE LINEAR MIXED EFFETS MODEL FOR THE CHANGE IN GAD-7 SCORE

	GAD-7 10 Weeks		GAD-7 22 Weeks	
	TAU N=1142	Sleepio N=733	TAU N=971	Sleepio N=603
**Unadjusted Mean (Standard Deviation)**	8.35 (6.06)	6.53 (5.40)	7.67 (6.10)	6.14 (5.41)
**Adjusted Difference (C.I.)***	-1.86 (-2.29; -1.43)		-1.56 (-2.01; -1.10)	
**p-value**	<0.0001		<0.0001	
* Linear mixed effects model adjusted for gender, student status, week and interaction of week with randomisation, and including a random effect for student within university. Covariance matrix of within subject measurements unstructured.				

3.6.2.8 ALTMAN MANIA SCORE

The Altman mania scale consists of five questions, scored from 0 to 4. Scores are summed to obtain overall score which can range from 0 to 20, with higher values indicating increasing probability of a manic or hypomanic condition. At 3 weeks the mean scores were similar to the baseline levels of the Altman mania scale, but the mean of the Sleepio group was significantly higher compared to the control group (adjusted difference (95% C.I.): 0.44 (0.21; 0.67); p-value = 0.0002) (Table 23). By 10 weeks the control group was lower than baseline levels, but the Sleepio group had higher levels, and was significantly higher compared to the control group (adjusted difference (95% C.I.): 0.93 (0.67; 1.19); p-value < 0.0001). At 22 weeks the mean Altman mania scores were similar compared to 10 weeks, with the Sleepio having significantly higher scores

compared to the control group (adjusted difference (95% C.I.): 0.75 (0.46; 1.03; p-value < 0.0001). The mean scores at baseline and at 3, 10 and 22 weeks are all below the level considered as having a high probability of manic symptoms.

TABLE 23 SUMMARY STATISTICS OF ALTMAN MANIA SCORE MEASURED AT 3, 10 AND 22 WEEKS, AND THE RESULTS OF THE LINEAR MIXED EFFECTS MODEL FOR THE CHANGE IN ALTMAN MANIA SCORE

	Altman 3 Weeks		Altman 10 Weeks		Altman 22 Weeks	
	TAU N=1398	Sleepio N=1044	TAU N=1142	Sleepio N=733	TAU N=971	Sleepio N=603
**Unadjusted Mean (Standard Deviation)**	3.44 (3.04)	3.83 (3.16)	2.97 (3.03)	3.77 (3.33)	2.92 (3.06)	3.57 (3.41)
**Adjusted Difference (C.I.)***	0.44 (0.21; 0.67)		0.93 (0.67; 1.19)		0.75 (0.46; 1.03)	
**p-value**	0.0002		<0.0001		<0.0001	

* Linear mixed effects model adjusted for gender, student status, week and interaction of week with randomisation, and including a random effect for student within university. Covariance matrix of within subject measurements unstructured.

3.6.2.9 Impairment in functioning, WSAS

The Work and Social Adjustment Scale (WSAS) assess participants' perceived impairment in functioning, with higher scores indicating greater perceived impairment. The summary statistics and results from the linear mixed effects model for change in WSAS score at 10 and 22 weeks appears in Table 24. The mean WSAS scores were lower at 10 and 22 weeks compared to the baseline levels for both treatment arms, but with the Sleepio group having significantly lower WSAS scores at 10 weeks (adjusted difference (95% C.I.): -4.36 (-5.03; -3.69); p-value < 0.0001) and at 22 weeks (adjusted difference (95% C.I.): -4.33 (-5.05; -3.62) ; p-value < 0.0001).

TABLE 24 SUMMARY STATISTICS OF WSAS MEASURED AT 10 WEEKS AND 22 WEEKS, AND THE RESULTS OF THE LINEAR MIXED EFFECTS MODEL FOR THE CHANGE IN WSAS SCORE

	WSAS 10 Weeks		WSAS 22 Weeks	
	TAU N=1142	Sleepio N=733	TAU N=971	Sleepio N=603
**Unadjusted Mean (Standard Deviation)**	15.92 (8.89)	11.43 (8.37)	14.92 (9.17)	10.25 (8.30)
**Adjusted Difference (C.I.)***	-4.36 (-5.03; -3.69)		-4.33 (-5.05; -3.62)	
**p-value**	<0.0001		<0.0001	

* Linear mixed effects model adjusted for gender, student status, week and interaction of week with randomisation, and including a random effect for student within university. Covariance matrix of within subject measurements unstructured.

3.6.2.10 Mental wellbeing, WEMWBS

The Warwick-Edinburgh Mental Wellbeing Scale (WEMWBS) consists of 14 items. Each item is rated from 1 (None of the time) to 5 (All the time). The 14 items are summed to give an overall score, which can range from 14 to 70, with higher scores indicating better wellbeing. Table 25 provides the summary statistics and results from the linear mixed effects model for change in the WEMWBS at 10 and 22 weeks. Both treatment arms have scores which are higher compared to baseline levels, indicating higher perceived wellbeing compared to

baseline. The Sleepio treatment arm had significantly higher improvement in the WEMWBS compared to the control arm at bother 10 weeks (adjusted difference (95% C.I.): 2.47 (1.72; 3.22); p-value < 0.0001) and at 22 weeks (adjusted difference (95% C.I.): 2.78 (1.97; 3.60); p-value < 0.0001).

TABLE 25 SUMMARY STATISTICS OF WEMWBS MEASURED AT 10 WEEKS AND 22 WEEKS, AND THE RESULTS OF THE LINEAR MIXED EFFECTS MODEL FOR THE CHANGE IN WEMWBS SCORE

	**WEMWBS 10 Weeks**		**WEMWBS 22 Weeks**	
	TAU N=1142	Sleepio N=733	TAU N=971	Sleepio N=603
**Unadjusted Mean (Standard Deviation)**	38.73 (9.78)	40.92 (9.63)	39.63 (10.19)	42.12 (10.36)
**Adjusted Difference (C.I.)***	2.47 (1.72; 3.22)		2.78 (1.97; 3.60)	
**p-value**	<0.0001		<0.0001	

* Linear mixed effects model adjusted for gender, student status, week and interaction of week with randomisation, and including a random effect for student within university. Covariance matrix of within subject measurements unstructured.

3.6.2.11 Development of later psychiatric problems

The analysis used to assess whether improved sleep will decrease the likelihood of developing later psychiatric problems: ultra-high risk of psychosis, bipolar affective disorder, depression and anxiety, was based on dichotomised outcomes. A binary variably on the presence of psychiatric problems was obtained from the self-report of treatment by mental health services, and further binary variables will be derived from the overall scores of the following questionnaires: PQ-16, PHQ-9, GAD-7, and Altman Mania Scale. The limit used to dichotomise the PQ-16 score was six, the PHQ-9 score was 10, the GAD-7 was 10, and the Altman Mania Scale was 6.

In the case of the SCI-8, Prodomal, PHQ-9, and GAD-7 scales, but not including the Altman mania scale, the odds ratio was significantly less than 1, indicating that it was less likely for participants in the Sleepio group to be classified as having a clinical condition (Table 26 and Table 27). In the case of the Altman mania scale, participants in the Sleepio group were more likely to be classified as manic compared to the TAU group (Table 26).

With regards to the odds of needing to make use of mental health services, having a current clinical diagnosis, using medication for mental health related issues, or having psychiatric therapy, there was no significant difference between the Sleepio group and TAU group (Table 27) at any of the assessment time points.

FINAL

TABLE 26 ODDS RATIO AND ADJUSTED ODDS RATIO FOR SLEEPIO VERSUS TAU OF EXCEEDING CLINICAL THRESHOLD AT 3 WEEKS, 10 WEEKS AND 22 WEEKS

	SCI-8 3 Weeks		SCI-8 10 Weeks		SCI-8 22 Weeks	
	TAU N=1398	Sleepio N=1044	TAU N=1142	Sleepio N=733	TAU N=971	Sleepio N=603
**Unadjusted odds ratio**	0.46 (0.39; 0.55)		0.25 (0.20; 0.30)		0.31 (0.25; 0.38)	
**Adjusted odds ratio (C.I)***	0.27 (0.20; 0.37)		0.08 (0.06; 0.12)		0.13 (0.09; 0.19)	
**p-value**	<0.0001		<0.0001		<0.0001	
	**Altman 3 Weeks**		**Altman 10 Weeks**		**Altman 22 Weeks**	
	TAU N=1398	Sleepio N=1044	TAU N=1142	Sleepio N=733	TAU N=971	Sleepio N=603
**Unadjusted odds ratio**	1.23 (1.02; 1.49)		1.63 (1.31; 2.04)		1.61 (1.25; 2.07)	
**Adjusted odds ratio (C.I)***	1.33 (1.02; 1.73)		2.01 (1.48; 2.73)		1.89 (1.34; 2.66)	
**p-value**	0.0340		<0.0001		0.0003	

* Logistic mixed effects model adjusted for gender, student status, week and interaction of week with randomisation, and including a random effect for student within university. Covariance matrix of within subject measurements unstructured.

TABLE 27 ODDS RATIO AND ADJUSTED ODDS RATION FOR SLEEPIO VERSUS TAU OF EXCEEDING CLINICAL THRESHOLD AT 10 AND 22 WEEKS

	PQ16 10 Weeks		PQ16 22 Weeks	
	TAU N=1142	Sleepio N=733	TAU N=971	Sleepio N=603
**Unadjusted odds ratio**	0.59 (0.47; 0.73)		0.63 (0.50; 0.80)	
**Adjusted odds ratio (C.I)***	0.26 (0.15; 0.46)		0.33 (0.18; 0.59)	
**p-value**	<0.0001		0.0003	
	**PHQ9 10 Weeks**		**PHQ-9 22 Weeks**	
	TAU N=1142	Sleepio N=733	TAU N=971	Sleepio N=603
**Unadjusted odds ratio**	0.46 (0.38; 0.56)		0.56 (0.45; 0.69)	
**Adjusted odds ratio (C.I)***	0.21 (0.14; 0.32)		0.32 (0.21; 0.48)	
**p-value**	<0.0001		<0.0001	
	**GAD-7 10 Weeks**		**GAD-7 22 Weeks**	
	TAU N=1142	Sleepio N=733	TAU N=971	Sleepio N=603
**Unadjusted odds ratio**	0.56 (0.45; 0.68)		0.63 (0.50; 0.79)	
**Adjusted odds ratio (C.I)***	0.32 (0.21; 0.48)		0.42 (0.27; 0.64)	
**p-value**	<0.0001		<0.0001	

	**Contacted Mental Health Services 10 Weeks**		**Contacted Mental Health Services 22 Weeks**	
	TAU N=1142	Sleepio N=733	TAU N=971	Sleepio N=603
**Unadjusted odds ratio**	1.04 (0.82; 1.32)		0.94 (0.72; 1.22)	
**Adjusted odds ratio (C.I)***	1.19 (0.70; 2.04)		0.98 (0.55; 1.75)	
**p-value**	0.5157		0.9362	
	**Diagnosis 10 Weeks**		**Diagnosis 22 Weeks**	
	TAU N=1142	Sleepio N=733	TAU N=971	Sleepio N=603
**Unadjusted odds ratio**	1.07 (0.88; 1.30)		1.14 (0.92; 1.41)	
**Adjusted odds ratio (C.I)***	1.33 (0.75; 2.37)		1.43 (0.78; 2.63)	
**p-value**	0.3260		0.2469	
	**Medication 10 Weeks**		**Medication 22 Weeks**	
	TAU N=1142	Sleepio N=733	TAU N=971	Sleepio N=603
**Unadjusted odds ratio**	0.89 (0.72; 1.11)		1.01 (0.80; 1.28)	
**Adjusted odds ratio (C.I)***	0.77 (0.47; 1.26)		0.96 (0.58; 1.59)	
**p-value**	0.3001		0.8655	
	**Psychiatric Therapy 10 Weeks**		**Psychiatric Therapy 22 Weeks**	
	TAU N=1142	Sleepio N=733	TAU N=971	Sleepio N=603
**Unadjusted odds ratio**	0.95 (0.69; 1.31)		0.72 (0.49; 1.06)	
**Adjusted odds ratio (C.I)***	1.27 (0.48; 3.35)		0.41 (0.11; 1.58)	
**p-value**	0.6269		0.1964	

* Logistic mixed effects model adjusted for gender, student status, week and interaction of week with randomisation, and including a random effect for student within university. Covariance matrix of within subject measurements unstructured.

3.7 Sensitivity Analyses

3.7.1 Missing Data Mechanism

3.7.1.1 SCI-8

The missing data mechanism was explored by means of a pattern mixture model. The results are displayed in Figure 4. If the participants missing from the Sleepio arm at 10 weeks had an average SCI-8 score less than 2 units lower compared to the non-missing (i.e. missing had worse sleep outcomes), then the treatment difference would still be significant between the Sleepio and TAU groups if the missing outcomes were available.

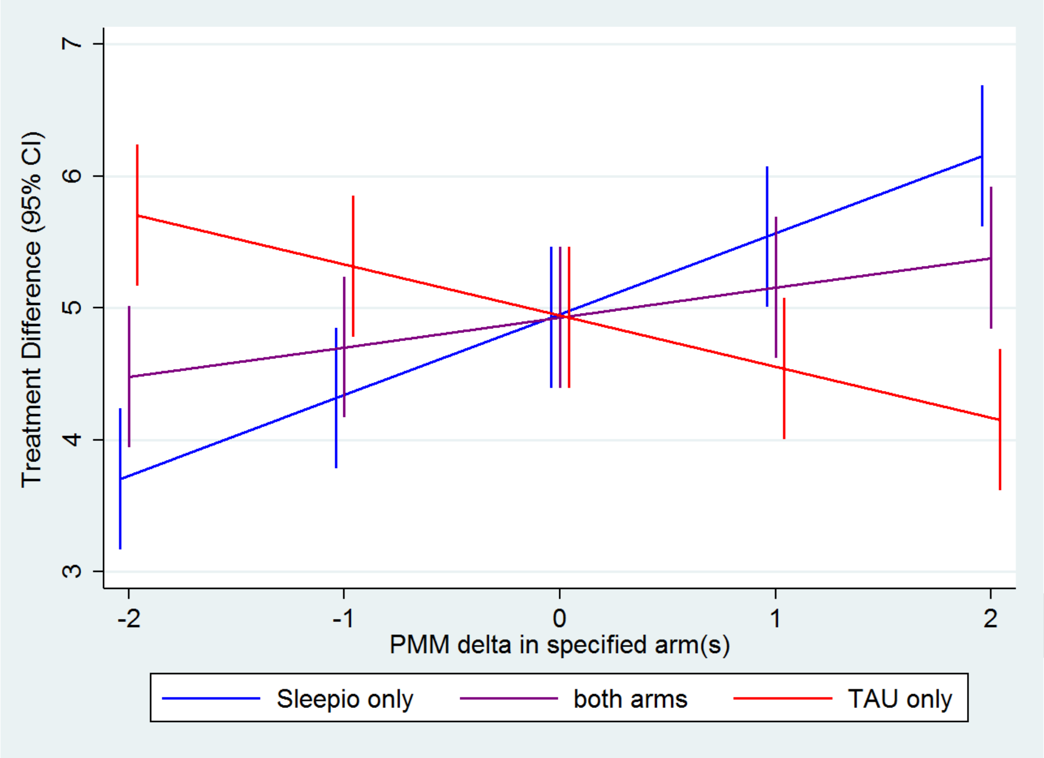

FIGURE 4 PATTERN MIXTURE MODEL RESULTS FOR THE SCI-8 OUTCOME AT 10 WEEKS

A sensitivity analysis was performed including baseline covariates which were found to be predictive of missingness of the SCI-8 outcome. Age was found to be predictive of missingness. Including this variable in the model increased the treatment difference very slightly (Table 28).

TABLE 28 LINEAR MIXED EFFECTS MODEL RESULTS FOR THE DIFFERENCE IN THE CHANGE IN SCI-8 SCORE BETWEEN THE SLEEPIO GROUP AND TAU GROUP WHEN AGE AND ETHNICITY ARE INCLUDE AS COVARIATES

	**SCI-8 3 Weeks**		**SCI-8 10 Weeks**		**SCI-8 22 Weeks**	
	TAU N=1398	Sleepio N=1044	TAU N=1142	Sleepio N=733	TAU N=971	Sleepio N=603
**Unadjusted Mean (Standard Deviation)**	12.34 (5.85)	14.96 (5.80)	13.31 (6.45)	18.08 (6.66)	14.43 (6.71)	19.27 (7.13)
**Adjusted Difference (C.I.)***	2.65 (2.21; 3.08)		4.84 (4.35; 5.32)		4.81 (4.28; 5.33)	
**p-value**	<0.0001		<0.0001		<0.0001	
* Linear mixed effects model adjusted for gender, age, student status, week and interaction of week with randomisation, and including a random effect for student within university. Covariance matrix of within subject measurements unstructured.						

A further sensitivity analysis was performed whereby missingness was assumed to be related to the outcome. The last observation carried forward method of imputation was used, where the last available measurement for a participant was imputed for all further missing measurements of that participants. In this pessimistic imputation of missing data, the treatment difference would still be significant but would more than half in magnitude (Table 29).

TABLE 29 LINEAR MIXED EFFECTS MODEL RESULTS FOR THE DIFFERENCE IN THE CHANGE IN SCI-8 SCORE BETWEEN THE SLEEPIO GROUP AND TAU GROUP WHEN THE MISSING DATA ARE IMPUTED USING LAST OBSERVATION CARRIED FOREWARD

	SCI-8 3 Weeks		SCI-8 10 Weeks		SCI-8 22 Weeks	
	TAU N=1864	Sleepio N=1891	TAU N=1864	Sleepio N=1891	TAU N=1864	Sleepio N=1891
**Unadjusted Mean (Standard Deviation)**	11.73 (5.63)	12.67 (5.77)	12.42 (6.18)	14.02 (6.70)	13.10 (6.47)	14.55 (7.14)
**Adjusted Difference (C.I.)***	1.04 (0.71; 1.38)		1.71 (1.38; 2.04)		1.55 (1.22; 1.88)	
**p-value**	<0.0001		<0.0001		<0.0001	

* Linear mixed effects model adjusted for gender, student status, week and interaction of week with randomisation, and including a random effect for student within university. Covariance matrix of within subject measurements unstructured.

3.7.1.2 Paranoia, GPTS

The missing data mechanism was explored by means of a pattern mixture model. The results are displayed in Figure 5. If the participants missing from the Sleepio arm at 10 weeks had an average GPTS score less than 2 units more than compared to the non-missing (i.e. the missing participants had higher levels of paranoia), then the treatment difference would still be significant between the Sleepio and TAU groups if the missing outcomes were available.

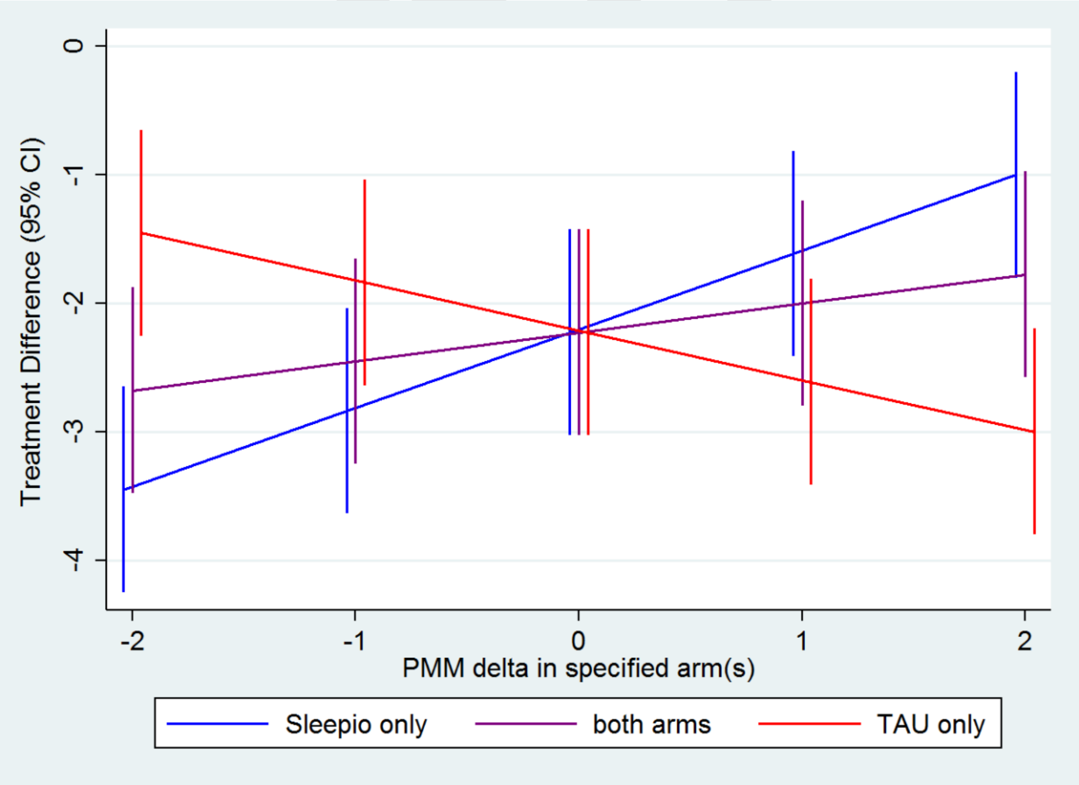

FIGURE 5 PATTERN MIXTURE MODEL RESULTS FOR THE GPTS OUTCOME AT 10 WEEKS

A sensitivity analysis was performed including baseline covariates which were found to be predictive of missingness of the GPTS outcome. Age was predictive of missingness. Including this variable in the model increased the magnitude of the treatment difference very slightly (Table 30).

TABLE 30 LINEAR MIXED EFFECTS MODEL RESULTS FOR THE DIFFERENCE IN THE CHANGE IN GPTS SCORE BETWEEN THE SLEEPIO GROUP AND TAU GROUP WHEN AGE AND ETHNICITY ARE INCLUDED AS COVARIATES

	**GPTS 3 Weeks**		**GPTS 10 Weeks**		**GPTS 22 Weeks**	
	TAU N=1398	Sleepio N=1044	TAU N=1142	Sleepio N=733	TAU N=971	Sleepio N=603
**Unadjusted Mean (Standard Deviation)**	24.63 (11.82)	22.61 (9.89)	23.84 (12.16)	21.06 (9.08)	23.84 (12.68)	20.75 (9.19)
**Adjusted Difference (C.I.)***	-1.84 (-2.52; -1.15)		-2.24 (-3.01; -1.47)		-2.78 (-3.61; -1.96)	
**p-value**	<0.0001		<0.0001		<0.0001	
* Linear mixed effects model adjusted for gender, age, student status, week and interaction of week with randomisation, and including a random effect for student within university. Covariance matrix of within subject measurements unstructured.						

A further sensitivity analysis was performed whereby missingness was assumed to be related to the outcome. The last observation carried forward method of imputation was used, where the last available measurement for a participant was imputed for all further missing measurements of that participants. In this pessimistic imputation of missing data, the treatment difference would still be significant but would more than half in magnitude (Table 31).

TABLE 31 LINEAR MIXED EFFECTS MODEL RESULTS FOR THE DIFFERENCE IN THE CHANGE IN GPTS SCORE BETWEN THE SLEEPIO GROUP AND TAU GROUP WHEN THE MISSING DATA ARE IMPUTED USING LAST OBSERVATION CARRIED FOREWARD

	**GPTS 3 Weeks**		**GPTS 10 Weeks**		**GPTS 22 Weeks**	
	TAU N=1864	Sleepio N=1891	TAU N=1864	Sleepio N=1891	TAU N=1864	Sleepio N=1891
**Unadjusted Mean (Standard Deviation)**	24.69 (11.72)	24.29 (11.39)	24.17 (11.94)	23.74 (11.36)	24.35 (12.38)	23.61 (11.44)
**Adjusted Difference (C.I.)***	-0.87 (-1.35; -0.39)		-0.90 (-1.37; -0.42)		-1.20 (-1.67; -0.72)	
**p-value**	0.0004		0.0002		<0.0001	
* Linear regression model adjusted for gender, student status, week and interaction of week with randomisation, and including a random effect for student within university. Covariance matrix of within subject measurements unstructured.						

3.7.1.3 HALLUCINATIONS, SPEQ

The missing data mechanism was explored by means of a pattern mixture model. The results are displayed in Figure 6. If the participants missing from the Sleepio arm at 10 weeks had an average SPEQ score less than 1 units more than compared to the non-missing (i.e. the missing participants had higher levels of paranoia), then the treatment difference would still be significant between the Sleepio and TAU groups if the missing outcomes were available. If the participants missing from the Sleepio arm at 10 weeks had an average SPEQ

FINAL

score less than 2 units more than compared to the non-missing (i.e. the missing participants had higher levels of paranoia), then the treatment difference would not be significant between the Sleepio and TAU groups if the missing outcomes were available.

A sensitivity analysis was performed including baseline covariates which were found to be predictive of missingness of the SPEQ outcome. Age were predictive of missingness. Including this variable in the model resulted in treatment differences that were identical or only slightly lower (in the case of week 22) to the primary analysis (Table 32).

A further sensitivity analysis was performed whereby missingness was assumed to be related to the outcome. The last observation carried forward method of imputation was used, where the last available measurement for a participant was imputed for all further missing measurements of that participants. In this pessimistic imputation of missing data, the treatment difference would still be significant but would more than half in magnitude (Table 33).

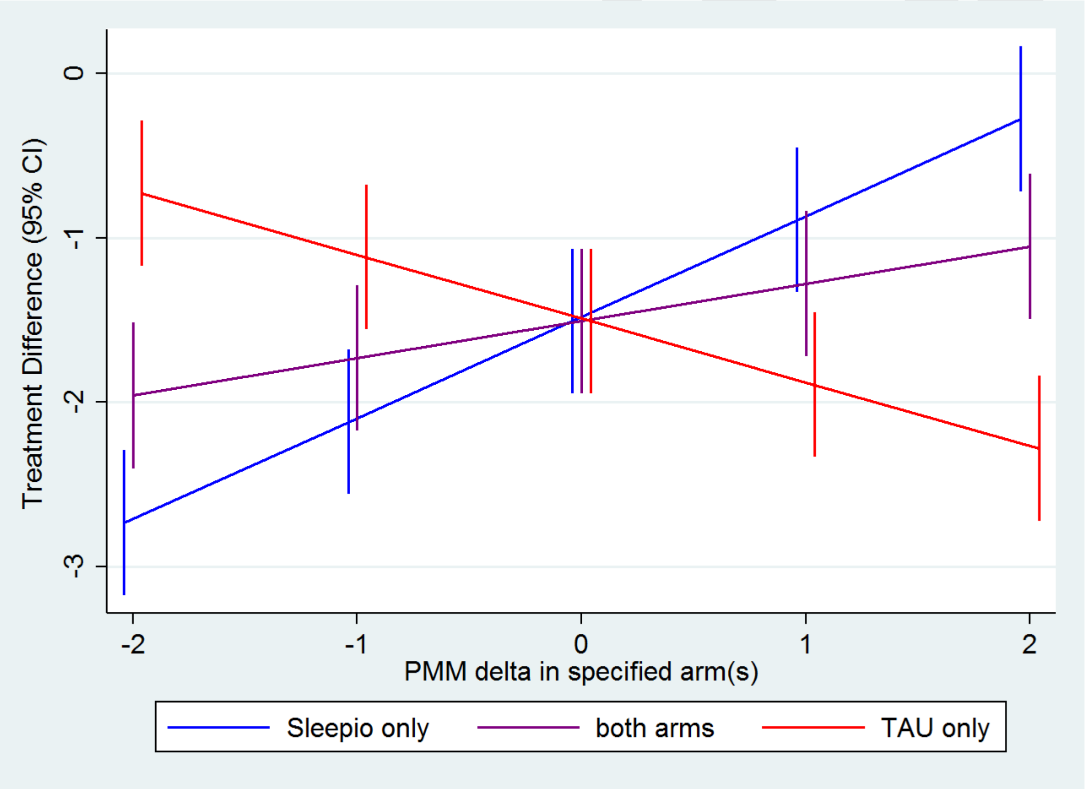

FIGURE 6 PATTERN MIXTURE MODEL RESULTS FOR THE SPEQ OUTCOME AT 10 WEEKS

TABLE 32 LINEAR MIXED EFFECTS MODEL RESULTS FOR THE DIFFERENCE IN THE CHANGE IN SPEQ SCORE BETWEEN THE SLEEPIO GROUP AND TAU GROUP WHEN AGE AND ETHNICITY ARE INCLUDED AS COVARIATES

	**SPEQ 3 Weeks**		**SPEQ 10 Weeks**		**SPEQ 22 Weeks**	
	TAU N=1398	Sleepio N=1044	TAU N=1142	Sleepio N=733	TAU N=971	Sleepio N=603
**Unadjusted Mean (Standard Deviation)**	5.06 (6.89)	4.06 (5.84)	4.89 (7.24)	3.12 (5.12)	4.71 (7.43)	2.87 (5.45)
**Adjusted Difference (C.I.)***	-0.79 (-1.15; -0.42)		-1.57 (-1.97; -1.17)		-1.54 (-1.96; -1.11)	
**p-value**	<0.0001		<0.0001		<0.0001	
* Linear mixed effects model adjusted for gender, age, student status, week and interaction of week with randomisation, and including a random effect for student within university. Covariance matrix of within subject measurements unstructured.						

TABLE 33 LINEAR MIXED EFFECTS MODEL RESULTS FOR THE DIFFERENCE IN THE CHANGE IN SPEQ SCORE BETWEEN THE SLEEPIO GROUP AND TAU GROUP WHEN THE MISSING DATA ARE IMPUTE USING LAST OBSERVATION CARRIED FOREWARD

	**SPEQ 3 Weeks**		**SPEQ 10 Weeks**		**SPEQ 22 Weeks**	
	TAU N=1864	Sleepio N=1891	TAU N=1864	Sleepio N=1891	TAU N=1864	Sleepio N=1891
**Unadjusted Mean (Standard Deviation)**	5.12 (6.84)	4.69 (6.19)	5.10 (7.21)	4.35 (6.14)	5.00 (7.20)	4.26 (6.18)
**Adjusted Difference (C.I.)***	-0.40 (-0.65; -0.15)		-0.72 (-0.97; -0.47)		-0.70 (-0.95; -0.45)	
**p-value**	0.0017		<0.0001		<0.0001	
*Linear mixed effects model adjusting for infant age and gender, infant temperament, PND severity, and socioeconomic status, with fixed effects for month and therapy arm, and random effects for participants.						

3.8 Safety Analyses

No adverse events were reported

4 References

Baron R, Kenny DA. The moderator-mediator variable distinction in social psychological research. *J Pers Soc Psychol* 1986; 5: 1173–82.

Espie CA, Kyle SD, Hames P, Gardani M, Fleming L, Cape J. The Sleep Condition Indicator: a clinical screening tool to evaluate insomnia disorder. BMJ Open. 2014;4(3):e004183.

Freeman D, Dunn G, Startup H, Pugh K, Cordwell J, Mander H, Cernis E, Wingham G, Shirvell K, Kingdon D. Effects of cognitive behavioural therapy for worry on persecutory delusions in patients with psychosis (WIT): a parallel, single-blind, randomised controlled trial with mediation analysis. *Lancet Psychiatry* 2015; 2: 305–13

Green CE, Freeman D, Kuipers E, Bebbington P, Fowler D, Dunn G, et al. Measuring ideas of persecution and social reference: the Green et al. Paranoid Thought Scales (GPTS). Psychol Med. 2008;38(1):101-11.

Hewitt CE, Torgerson DJ. Is restricted randomisation necessary? Br Med J. 2006;332(June):1506–8.

Ronald A, Sieradzka D, Cardno AG, Haworth CM, McGuire P, Freeman D. Characterization of psychotic experiences in adolescence using the specific psychotic experiences questionnaire: findings from a study of 5000 16-year-old twins. Schizophr Bull. 2014;40(4):868-77.

Schults KF and Grimes DA. Multiplicity in clinical trials I: endpoints and treatments. Lancet. 2005; 365:1591-1595.

5 APPENDICES

Appendix I. Flow diagram of trial participants

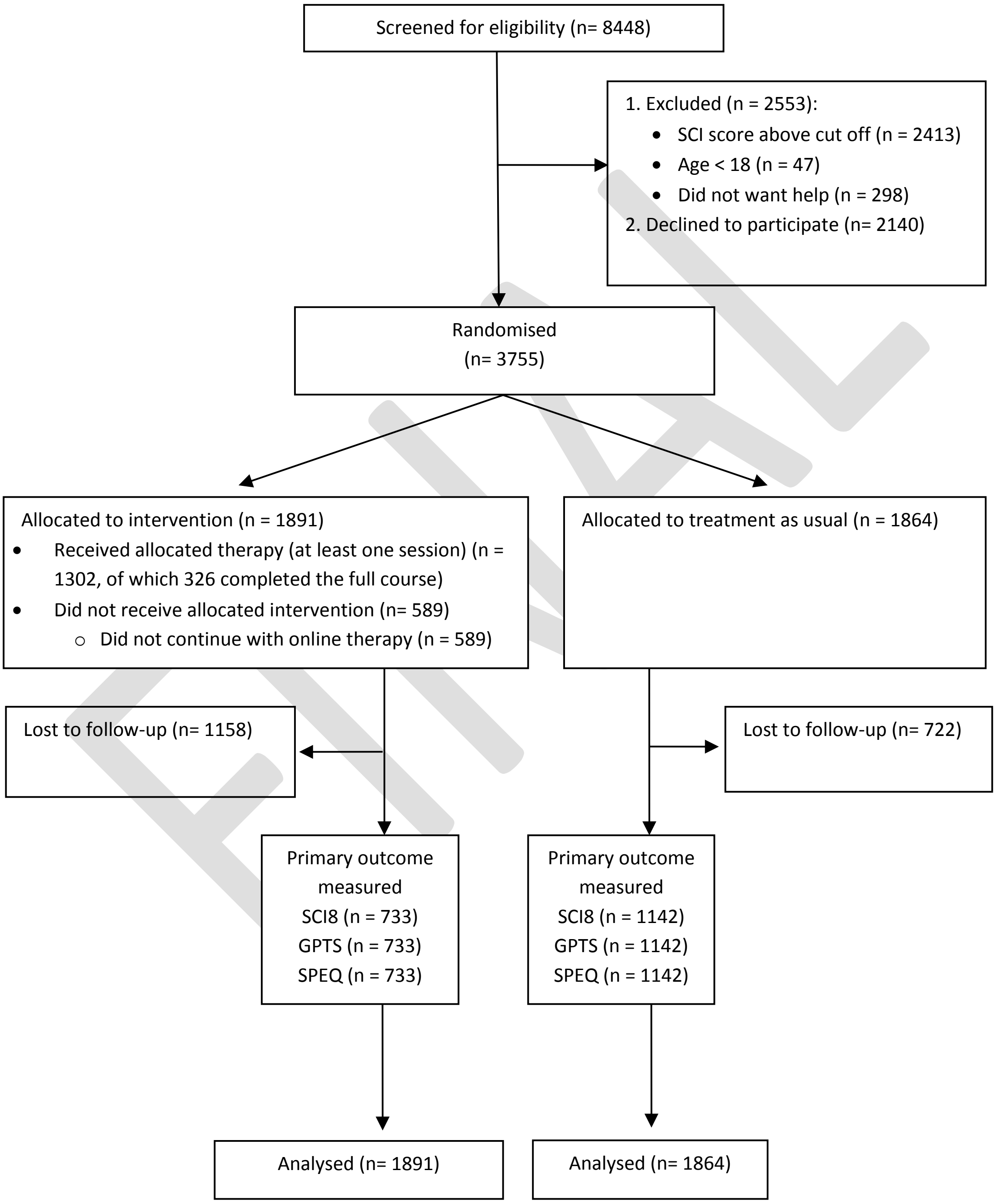

FINAL

Appendix II: Loss to follow-up during the course of the study

Outcome	N Analysed (% Loss to follow-up)							
	0 weeks		3 weeks		10 weeks		22 weeks	
	TAU	Sleepio	TAU	Sleepio	TAU	Sleepio	TAU	Sleepio
**SCI8**	1864 (0.0%)	1891 (0.0%)	1398 (25.0%)	1044 (44.8%)	1142 (38.7%)	733 (61.2%)	971 (47.9%)	603 (68.1%)
**GPTS**	1864 (0.0%)	1891 (0.0%)	1398 (25.0%)	1044 (44.8%)	1142 (38.7%)	733 (61.2%)	971 (47.9%)	603 (68.1%)
**SPEQ**	1864 (0.0%)	1891 (0.0%)	1398 (25.0%)	1044 (44.8%)	1142 (38.7%)	733 (61.2%)	971 (47.9%)	603 (68.1%)
**SCI9**	1864 (0.0%)	1891 (0.0%)	1398 (25.0%)	1044 (44.8%)	1142 (38.7%)	733 (61.2%)	971 (47.9%)	603 (68.1%)
**ISI**	1864 (0.0%)	1891 (0.0%)			1142 (38.7%)	733 (61.2%)	970 (48.0%)	603 (68.1%)
**DDNSI**	1864 (0.0%)	1891 (0.0%)			1142 (38.7%)	733 (61.2%)	963 (48.3%)	599 (68.3%)
**PQ16**	1864 (0.0%)	1891 (0.0%)			1142 (38.7%)	733 (61.2%)	971 (47.9%)	603 (68.1%)
**PHQ9**	1864 (0.0%)	1891 (0.0%)			1142 (38.7%)	733 (61.2%)	971 (47.9%)	602 (68.2%)
**PHQ4**			1398 (25.0%)	1044 (44.8%)				
**GAD7**	1864 (0.0%)	1891 (0.0%)			1142 (38.7%)	733 (61.2%)	971 (47.9%)	603 (68.1%)
**Altman Mania**	1864 (0.0%)	1891 (0.0%)	1398 (25.0%)	1044 (44.8%)	1142 (38.7%)	733 (61.2%)	971 (47.9%)	603 (68.1%)
**WSAS**	1864 (0.0%)	1891 (0.0%)			1142 (38.7%)	733 (61.2%)	971 (47.9%)	603 (68.1%)
**WEMWBS**	1864 (0.0%)	1891 (0.0%)			1142 (38.7%)	733 (61.2%)	971 (47.9%)	603 (68.1%)
**Contacted Mental Health Services**	1864 (0.0%)	1891 (0.0%)			1142 (38.7%)	733 (61.2%)	971 (47.9%)	603 (68.1%)
**Diagnosis**	1864 (0.0%)	1891 (0.0%)			1142 (38.7%)	733 (61.2%)	971 (47.9%)	603 (68.1%)
**Medication**	1864 (0.0%)	1891 (0.0%)			1142 (38.7%)	733 (61.2%)	971 (47.9%)	603 (68.1%)
**Psychological Therapy**	1864 (0.0%)	1891 (0.0%)			1142 (38.7%)	733 (61.2%)	971 (47.9%)	603 (68.1%)

Appendix III: Outcome assessment schedule

	**Screening**	**3 Weeks Assessment**	**10 Weeks Assessment**	**22 Weeks Assessment**
	**Screening and Baseline**	**3 Weeks post-randomisation (7 weeks of treatment left)**	**10 Weeks post-randomisation (End of treatment)**	**22 Weeks post-randomisation (12 weeks after treatment completion)**
**Primary outcome**				
Sleep Condition Indicator (SCI-8)	X	X	X	X
Green Paranoid Thoughts Scale (GPTS)	X	X	X	X
Specific Psychotic Experiences Questionnaire (SPEQ)	X	X	X	X
Secondary outcome				
Sleep Condition Indicator (SCI-9)	X	X	X	X
Insomnia Severity Index (ISI)		X	X	X
Disturbing Dreams and Nightmare Severity Index (DDNSI)		X	X	X
Work and Social Adjustment Scale (WSAS)		X	X	X
Prodromal Questionnaire (PQ-16)		X	X	X
Patient Health Questionnaire (PHQ-9)		X	X	X
Patient Health Questionnaire (PHQ-4)		X	X	X
Generalised Anxiety Disorder (GAD-7)		X	X	X
Altman Mania Scale	X	X	X	X
Warwick-Edinburgh Mental Wellbeing Scale (WEMWBS)		X	X	X
Current contact with mental health services		X	X	X
Current diagnosis		X	X	X
Current prescribed medications		X	X	X
Current receipt of psychological therapy		X	X	X
**Adverse events**	X	X	X	X
**Key covariates**				
Demographics (age, gender etc)	X			
