# Supplementary material for: The effects of improving sleep on mental health (OASIS): a randomised controlled trial with mediation analysis
Source: Lancet Psychiatry. 2017 Oct;4(10):749–58. doi: 10.1016/S2215-0366(17)30328-0 (PMC5614772; doi:10.1016/S2215-0366(17)30328-0)

THE LANCET
Psychiatry

Supplementary appendix 2

This appendix formed part of the original submission and has been peer reviewed. We post it as supplied by the authors.

Supplement to: Freeman D, Sheaves B, Goodwin GM, et al. The effects of improving sleep on mental health (OASIS): a randomised controlled trial with mediation analysis. *Lancet Psychiatry* 2017; published online Sept 6. http://dx.doi.org/10.1016/S2215-0366(17)30328-0.

OASIS

STATISTICAL ANALYSIS PLAN

Version 1.0

18 August 2016 (support Protocol v. 3 dated 12/02/2015)

Title: A parallel group, randomised controlled trial of digital cognitive behavioural therapy for insomnia versus treatment as usual: the impact of improved sleep on mental health in a student sample.

Short title: OASIS

Ethics Ref: MSD-IDREC-C2-2014-034

	NAME	TITLE	SIGNATURE	DATE
Written by:	Alecia Nickless	Trial Statistician		30-8-16
Reviewed by:	Ly-Mee Yu	Lead Statistician		30/8/2016
Approved by:	Daniel Freeman	Chief Investigator		25-8-16

Version History

Version:	Version Date:	Changes:
0.1	29 January 2016	Original Version by Nicola Williams
0.2	13 April 2016	Modified by Alecia Nickless to comply with the published protocol
0.3	26 May 2016	Further modified to match correctly with protocol
0.4	23 June 2016	Incorporating comments from co-applicants. Placing work and social adjustment scale.
0.5	28 July 2016	Incorporated comments from Richard Emsley and corrected the number of items in the SCI score to be nine items as per protocol.
0.6	9 August 2016	Included statement on multiple testing
0.7	11 August 2016	Included self-report from mental health services in the outcome description
1.0	24 August 2016	Correction – removal of over the counter medication outcome

FINAL

FINAL

Table of Contents

Table of Contents 3
1 Introduction 5
1.1 Preface 5
1.2 Purpose and scope of the plan 5
1.3 Trial overview 5
1.4 Objectives 5
2 Trial design 6
2.1 Outcomes measures 6
2.1.1 Primary outcomes 6
2.1.2 Secondary outcomes 7
2.2 Target population 8
2.3 Sample size 9
2.4 Randomisation and blinding in the analysis stage 9
3 Analysis – General considerations 9
3.1 Descriptive statistics 9
3.2 Characteristics of participants 9
3.3 Definition of population for analysis 9
3.4 Data Monitoring Committee And Interim Analyses 10
3.5 Multiple Testing 10
4 PRIMARY ANALYSIS 10
4.1 Primary outcomes 10
4.2 Handling missing data 11
4.3 Handling outliers 11
4.4 Multiple comparisons and multiplicity 11
4.5 Model assumptions 11
5 SECONDARY ANALYSIS 11
5.1 Primary outcome 11
5.2 Secondary outcomes 12
6 SENSITIVITY ANALYSIS 12
7 SUBGROUP ANALYSES 12
8 EXPLORATORY ANANLYSIS 12
9 SAFETY ANALYSIS 13
10 CHANGES TO THE PROTOCOL OR PREVIOUS VERSIONS OF SAP 13

FINAL

11 References 14

12 Appendices 16

FINAL

1 INTRODUCTION

1.1 PREFACE

Trial statistician: Ly-Mee Yu

Chief Investigator: Professor Daniel Freeman

Trial Manager: Bryony Sheaves

This SAP supports version 3.0 12/02/2015 of the protocol.

1.2 PURPOSE AND SCOPE OF THE PLAN

This document details the proposed analysis of the main paper(s) reporting results from the Wellcome trust funded randomised controlled trial to evaluate the use of digital cognitive behavioural therapy for insomnia (CBTi) versus treatment as usual (TAU). The results reported in these papers should follow the strategy set out here. Subsequent analyses of a more exploratory nature will not be bound by this strategy, though they are expected to follow the broad principles set out here. The principles are not intended to curtail exploratory analysis (for example, to decide cut-points for categorisation of continuous variables), nor to prohibit accepted practices (for example, data transformation prior to analysis), but they are intended to establish the rules that will be followed, as closely as possible, when analysing and reporting the trial.

The analysis strategy will be available on request when the principal papers are submitted for publication in a journal. Suggestions for subsequent analyses by journal editors or referees, will be considered carefully, and carried out as far as possible in line with the principles of this analysis strategy; if reported, the source of the suggestion will be acknowledged.

Any deviations from the statistical analysis plan will be described and justified in the final report of the trial. The analysis should be carried out by an identified, appropriately qualified and experienced statistician, who should ensure the integrity of the data during their processing. Examples of such procedures include quality control and evaluation procedures.

1.3 TRIAL OVERVIEW

Insomnia is a common psychological disorder which can lead to other psychological disorders such as depression, anxiety and psychosis. A digital cognitive behavioural therapy may improve sleep and in turn lead to improved mental health.

1.4 OBJECTIVES

Primary objectives

1. To assess whether delivering a digital cognitive behavioural therapy for the treatment of insomnia (CBTi) improves insomnia symptoms in a sample of university students by the end of treatment (10 weeks post-randomisation).

2. To assess whether web delivered CBTi results in a reduction in psychotic-like experiences (paranoia and hallucinations) by the end of treatment (10 weeks post-randomisation).
3. To assess whether changes in insomnia symptoms will mediate the changes in psychotic-like experiences by the end of treatment (10 weeks post-randomisation).

Secondary objectives

1. To determine whether web delivered CBTi improves levels of depression, anxiety, nightmares, and mania by the end of treatment (10 weeks post-randomisation).
2. To determine whether web-based CBTi improves psychological wellbeing by the end of the treatment (10 weeks post-randomisation).
3. To determine if the effects of CBTi on the primary and secondary outcomes will be maintained at the scheduled follow-up assessment (22 weeks post-randomisation).
4. To determine if CBTi will lead to the occurrence of fewer mental health disorders during the period of the trial, as assessed by screening tools at 22 weeks post-randomisation for ultra-high risk of psychosis, bipolar affective disorder, depression, and anxiety, and by treatment by mental health services.

2 Trial design

OASIS is a single blinded individual patient randomised controlled trial. A sample of 3754 university students presenting with symptoms of insomnia will be recruited and randomised to receive either cognitive behavioural therapy for insomnia plus treatment as usual, or treatment as usual (1:1).

2.1 Outcomes measures

Outcome measures are assessed at baseline and follow up (3, 10 and 22 weeks post-randomisation) and all data is collected via the web based platform. Weeks 0-22 are the main trial, to which this statistical analysis plan refers. At week 23 post-randomisation all participants in the treatment as usual group will be offered digital CBTi for help with their sleep problems. Following completion of the CBTi programme, all participants will again be asked to complete an assessment. This will be at week 33 post-randomisation.

See Appendix I for a table of outcomes assessment schedule.

In addition to the formal assessments in Appendix I the CBTi (Sleepio.com) system provides online analytics. These can be used for example to monitor adherence by assessing how many sessions were completed and the number of weeks to complete the full CBTi course. These will be used in exploratory analyses.

2.1.1 Primary outcomes

The primary outcome to assess for improvements in insomnia is the Sleep Condition Indicator (SCI) at 10 weeks (weeks 3 and 22 are secondary). The SCI total score is calculated by adding together the scores for the eight items. Each item ranges between 0 and 4, and the total score can range between 0 and 36. Higher scores indicate better sleep (Espie et al. 2014).

FINAL

The primary outcomes to assess for a reduction in psychotic-like experiences are the Green Paranoid Thoughts Scale (GPTS) to assess paranoia and the Specific Psychotic Experiences Questionnaire (SPEQ) – Hallucinations to assess hallucinations at 10 weeks (weeks 3 and 22 are secondary). The GPTS assessment measures two dimensions of paranoid thinking: ideas about social reference and ideas about social persecution. Each dimension consists of 16 statements which are then rated according how true the subject believes the statement to be on a Linkert scale from 1 (don't believe at all) to 5 (totally believe). The total score for each dimension is obtained by summing all 16 responses, ranging from 16 to 80, with higher scores reflecting higher levels of paranoia (Green et al. 2008). Only Part B is completed by participants. We will use part B of this assessment on social persecution as the primary outcome measuring levels of paranoia.

The SPEQ assessment considers six different types of psychotic experiences: paranoia, hallucinations, cognitive disorganisation, grandiosity, anhedonia (all by means of self-report), and negative symptoms (via parent report). Only the SPEQ hallucination subscale is given to participants. The subscale for hallucination will be used as a primary outcome for hallucinatory experiences (which is one of two psychotic experiences to be tested in the primary analysis). This subscale consists of nine items. These items are measured on a 6-point scale (not at all (0), once per fortnight (1), once per week (2), several times per week (3), daily (4), more than once per day (5)) and the overall score, calculated by summing the nine responses, ranges from 0 to 45 (Ronald et al. 2014).

2.1.2 Secondary outcomes

Listed below are the secondary outcomes, along with the objective to which they relate. Assessment points are at week 0, 3, 10 and 22 post-randomisation but the 10 week outcome is of primary importance in all cases. Only the primary outcomes and the Altman mania scale are assessed at week 3. Further details of the questionnaires can be found in Appendix I.

- To determine whether web delivered CBTi improves sleep: reducing insomnia:
  - Insomnia Severity Index - 7 questions, scored from 0 to 4. Scores are summed to obtain overall score which can range from 0 to 28, with higher values indicating increasing levels of insomnia. (0, 10, and 22 weeks post-randomisation)
  - The nine-item SCI - includes one additional question regarding early morning waking. (0, 3, 10, and 22 weeks post-randomisation)
- To determine whether web delivered CBTi improves levels of depression:
  - PHQ-9 questionnaire - 9 questions, scored from 0 to 3. Scores are summed to obtain overall score which can range from 0 to 27, with higher values indicating increasing levels of depression. (0, 10, and 22 weeks post-randomisation)
- To determine whether web delivered CBTi improves levels of anxiety:
  - GAD-7 questionnaire - 7 questions, scored from 0 to 3. Scores are summed to obtain overall score which can range from 0 to 21, with higher values indicating increasing levels of anxiety. (0, 10, and 22 weeks post-randomisation)
- To determine whether web delivered CBTi reduces the severity of nightmares:
  - Disturbing dream and nightmare severity index – 5 questions. It measures the number of nights with nightmares per week (0-7 nights) and number of total nightmares per week. The

DDNSI also measures the severity and intensity of the nightmares on a Likert-type scale ranging from no problem (0) to extremely severe problem (6), as well as how often nightmares result in awakenings ranging from never/rarely (0) to always (4). The index score is calculated by adding the number of nightmares per week (up to 14), number of nights with nightmares per week, and ratings of the severity of the nightmares, the intensity of the nightmares, and the frequency of nightmare-related awakenings. The score can range from 0 to 37, with higher values indicating a higher risk of a clinically salient nightmare complaint.

- To determine whether web-based CBTi results in reduction of mania-like symptoms:
    - Altman mania scale - 5 questions, scored from 0 to 4. Scores are summed to obtain overall score which can range from 0 to 20, with higher values indicating increasing probability of a manic or hypomanic condition.
- To determine whether web-based CBTi improves psychological wellbeing by the end of the treatment:
    - Warwick-Edinburgh mental wellbeing scale – 14 items. Each item is rated from 1 (None of the time) to 5 (All the time). The 14 items are summed to give an overall score, which can range from 14 to 70, with higher scores indicating better wellbeing.
- The outcomes of the following assessments will be dichotomised and used to assess whether improved sleep will decrease the likelihood of developing later psychiatric problems (assessed at 22 weeks): psychosis, bipolar affective disorder, depression and anxiety:
    - Prodromal questionnaire - 16 questions, scored from 0 to 2 (no, mild, moderate). Scores are summed to obtain overall score which can range from 0 to 16, with higher values indicating increasing risk of psychosis. (Dichotomisation limit = ≥ 6)
    - GAD-7 (Dichotomisation limit = ≥ 10)
    - PHQ-9 (Dichotomisation limit = ≥ 10)
    - Altman mania scale (Dichotomisation limit = ≥ 6)
    - Self-report of treatment by mental health services (current contact with mental health services, current diagnosis, current prescribed medications, current receipt of psychological therapy

2.2 TARGET POPULATION

Inclusion criteria

- Symptoms of insomnia, indicated by the sleep condition indicator
- Age ≥16
- Student from a UK university

Exclusion Criteria

- None

FINAL

2.3 Sample size

There are two primary outcomes: sleep (as measured by the SCI questionnaire) and psychotic like experiences (paranoia and hallucinations) (as measured by the GPTS and SPEQ). The sleep primary outcome would expect to find a larger standardised mean difference than psychotic-like experiences; hence psychotic symptoms have been used to determine the sample size in order to provide a conservative power calculation.

According to the original protocol, a sample size of 2614 would be collected. This would provide 90% power to detect a standardised mean difference of 0.15 in psychotic-like experiences (primary outcome), whilst accounting for a high level of expected attrition (40%). In a study amendment the sample size was increased, as drop-out rates were proving higher. Therefore 3754 participants were recruited (1877 per treatment arm).

2.4 Randomisation and blinding in the analysis stage

Once participants have completed the baseline assessment (week 0), they will be randomised to the sleep improvement programme (delivered by sleepio.com) in addition to TAU, or to TAU alone. The size of the two groups will be even. Randomisation will be completed via an automated system. The study will use simple randomisation with an allocation ratio of 1:1, as recommended for large trials (Hewitt and Torgerson 2006). Participants will be informed of the outcome of randomisation by receipt of an email.

The study is single blinded, as the participants are aware of which arm of the trial they are allocated to, but the researcher assessors are blinded to the study arm of the participant.

FINAL

3 Analysis – General considerations

3.1 Descriptive statistics

Frequencies (with percentages) for binary and categorical variables and means (and standard deviations), or medians (with lower and upper quartiles) for continuous variables will be presented by intervention group as well as the total.

3.2 Characteristics of participants

Baseline characteristics of the patients (demographics and baseline results on the questionnaires) will be reported by randomised group as well as the total.

There will be no tests of statistical significance nor confidence intervals for differences between randomised groups on any baseline variables.

Patient throughput from screening through randomisation, follow up and analysis will be presented in a CONSORT flow chart (Appendix II).

3.3 Definition of population for analysis

All data will be included in the analysis as far as possible to allow full ITT analysis. Patients will be analysed in the groups they were allocated, irrespective of whether they received that intervention or not. For the

complier average causal effect (CACE) analysis in section 5.1., compliance to the protocol will be taken into account.

3.4 Data Monitoring Committee And Interim Analyses

The trial does not have a formal data monitoring committee and there are no planned interim analyses.

3.5 Multiple Testing

No adjustment will be made for multiple testing for the analyses performed on the primary outcomes as these hypotheses are pre-specified, highly correlated and related to each other (Schultz and Grimes, 2005).

4 PRIMARY ANALYSIS

4.1 Primary outcomes

The primary outcomes: Sleep Condition Indicator (SCI), Green Paranoid Thoughts Scale (GPTS), and Specific Psychotic Experiences Questionnaire - Hallucinations (SPEQ) will be analysed in the following manner:

Each outcome will be analysed using a mixed effects regression model to account for the repeated measures over time. All non-missing scores will be included with missing values implicitly accounted for within the model. Baseline score will be entered as a covariate in the model and not as a response. The mixed effect model will include the outcome as the response variable; time point (3 weeks, 10 weeks or 22 weeks), randomised group (CBTi or TAU) and baseline score as fixed effects and a patient specific random intercept. An interaction between time and randomised group will be fitted as a fixed effect to allow estimation of treatment effect at all three time points (3 weeks, 10 weeks or 22 weeks). The following baseline factors will also be included as covariates in the model: gender, level currently studying (undergraduate, post-graduate or 'other') and from which university the participant was recruited. An unstructured correlation matrix will be used to model the within-subject error correlation structure. The primary outcome is the score at 10 weeks. Results will be presented as mean difference in score between randomised groups at 10 weeks with 95% confidence intervals (CI) and associated two-sided p value. Adjusted and unadjusted mean difference in scores between randomised groups at 3 weeks and 22 weeks will also be presented with 95% confidence intervals and associated p values. The adjusted mean difference will be of primary interest.

It is expected that there will be moderate skewness of the hallucination experiences scale as measured by SPEQ (Ronald et al. 2014). If this is the case, then the p-values and confidence intervals for the model parameters will be obtained by means of bootstrapping.

Modern causal inference methods will be used to determine whether changes in insomnia will mediate the changes in psychotic-like experiences. Two separate analyses will be conducted, one for the GPTS score and one for the SPEQ hallucination experiences score. In each case, a parametric regression model will be used to test for mediation of the digital CBTi through insomnia on psychotic-like experience. Adjustment will be made for baseline measures of the mediator (SCI score), baseline measures of the outcome (GPTS score or SPEQ hallucinations score), time point, and baseline covariates (gender, level currently studying, and from which

university the participant was recruited). We will also include repeated measures of the mediator and outcome to account for classical measurement error and baseline confounding, as well as sensitivity analysis in order to investigate the sensitivity of the estimates to these problems and that of unmeasured confounding (Emsley et al. 2010). This might include instrumental variable methods where terms are included for the interaction between randomisation and baseline covariates as potential instruments, if any interactions are present in the data.

4.2 HANDLING MISSING DATA

The frequency (with percentage) of losses to follow-up (defaulters and withdrawals) over the course of the study will be reported by randomised group and compared between the groups. Any deaths and their causes will be reported separately.

The availability of the outcome data for the primary outcomes will be summarised by randomised group. The mixed effects model implicitly accounts for data missing at random, however the data missingness mechanism will be explored. A logistic regression model will explore any association between baseline characteristics and availability of the primary outcome.

Any changes to the assumptions made in the primary analysis i.e. data missing at random, will be considered in a sensitivity analysis.

4.3 HANDLING OUTLIERS

Any outliers will be checked and verified to ensure that they are true values. Once they have been confirmed, a sensitivity analysis will be carried out to assess the impact of these values on the results.

4.4 MULTIPLE COMPARISONS AND MULTIPLICITY

The primary outcomes are clearly stated in the protocol and no adjustments for multiple comparisons will be made.

4.5 MODEL ASSUMPTIONS

The distribution of the primary outcomes will be assessed and the assumptions of the models will be checked. If any of the assumptions are violated, then p-values and confidence intervals for the model coefficients will be obtained by means of bootstrapping.

5 SECONDARY ANALYSIS

5.1 PRIMARY OUTCOME

A complier-average causal effect (CACE) analysis will be used to determine what effect the level of compliance to the intervention has on the treatment effect.

FINAL

5.2 Secondary outcomes

The secondary outcomes will be analysed using the same methods detailed above for the individual primary outcomes. In all cases an interaction between time and randomised group will be fitted as a fixed effect to allow estimation of treatment effect at all three time points (3 weeks (where available), 10 weeks or 22 weeks), however, 10 weeks is considered of primary importance for all outcomes.

The analysis used to assess whether improved sleep will decrease the likelihood of developing later psychiatric problems (assessed at 22 weeks): psychosis, bipolar affective disorder, depression and anxiety, will be based on dichotomised outcomes. A binary variably on the presence of psychiatric problems will be obtained from the self-report of treatment by mental health services, and further binary variables will be derived from the overall scores of the following questionnaires: Prodomal, PHQ-9, GAD-7, and Altman Mania Scale. The limit used to dichotomise the Prodomal score is six, the PHQ-9 score is 10, the GAD-7 is 10, and the Altman Mania Scale is 6. The analysis will be by means of logistic regression model, where a main effect will be included for randomised group and adjustment will be made for the following baseline variables: gender, level currently studying (undergraduate, post-graduate or 'other') and which university the participant was recruited from and the baseline level of the score. The unadjusted and adjusted odds ratio between the control and intervention will be reported for each binary outcome. The binary outcomes used to assess contact with mental health services will be analysed in the same way.

6 SENSITIVITY ANALYSIS

Sensitivity analysis will examine the robustness of the results to different assumptions regarding departures from randomisation policies and missing data. A pattern mixture model will be applied to the data allowing informative missing parameters to express the magnitude of departure from MCAR.

To determine to what extent treatment dilution due to non-compliance was a factor in the primary analysis, a completer-only analysis will be conducted and the results compared to the primary analysis by examining the treatment difference and its confidence interval.

Sensitivity analyses will be performed on the mediation analysis in order to investigate the sensitivity of the estimates to problems of classical measurement error, baseline confounding and that of unmeasured confounding (Emsley et al. 2010). This might include instrumental variable methods where terms are included for the interaction between randomisation and baseline covariates as potential instruments, if any interactions are present in the data.

7 SUBGROUP ANALYSES

No subgroup analyses were specified in the protocol.

8 EXPLORATORY ANANLYSIS

The CBTi (Sleepio.com) system provides online analytics. These can be used, for example, to monitor adherence by assessing how many sessions were completed and the number of weeks to complete the full CBTi course. The means and 95% confidence intervals will be reported for the number of sessions attended and the number of weeks it takes to complete the programme. This will only be applicable to the CBTi group.

FINAL

9 SAFETY ANALYSIS

We will record the occurrence of any serious adverse events in trial participants, defined as: all deaths, suicide attempts, serious violent incidents, admissions to secure units, and formal complaints about the online intervention. Owing to the online nature of the assessments and intervention, it is unlikely that the research team will become aware of all such events. Adverse events are likely to come to our attention only if we are contacted by a trial participant. For participants concerned about their mental health, a list of UK support services is provided on the study website. If a participant makes contact via email or telephone then the clinical psychologist coordinating the trial can advise on appropriate clinical services. All SAEs will be listed with information regarding group patient was randomised to, date of randomisation, date of SAE, details of the SAE and whether the SAE was related to the intervention.

A comparison of serious adverse events between the CBTi and TAU groups will be assessed by reporting the total number of adverse events and the number of patients with at least one adverse event in each randomised group. The analysis will be conducted by intention to treat. The randomised groups will be compared using a Chi squared test or Fisher's Exact test looking at the number of patients with at least one adverse event. The difference in proportions and 95% CI will be reported.

10 CHANGES TO THE PROTOCOL OR PREVIOUS VERSIONS OF SAP

Sample size was increased as the drop-out rates were found to be much higher than initially anticipated. Originally the drop-out rate were assumed to be 40%, but was found to be much higher, and therefore the sample size was doubled compared to what was actually required to power the study.

FINAL

11 References

Altman EG, Hedeker D, Peterson JL, Davis JM. The Altman Self-Rating Mania Scale. Biol Psychiatry. 1997;42(10):948-55.

Bastien CH, Vallieres A, Morin CM. Validation of the Insomnia Severity Index as an outcome measure for insomnia research. Sleep Med. 2001;2(4):297-307.

Emsley R, Dunn G, White IR. Mediation and moderation of treatment effects in randomised controlled trials of complex interventions. Stat. Methods in Med. Res. 2010; 237-270.

Espie CA, Kyle SD, Hames P, Gardani M, Fleming L, Cape J. The Sleep Condition Indicator: a clinical screening tool to evaluate insomnia disorder. BMJ Open. 2014;4(3):e004183.

Green CE, Freeman D, Kuipers E, Bebbington P, Fowler D, Dunn G, et al. Measuring ideas of persecution and social reference: the Green et al. Paranoid Thought Scales (GPTS). Psychol Med. 2008;38(1):101-11.

Hewitt CE, Torgerson DJ. Is restricted randomisation necessary? Br Med J. 2006;332(June):1506–8.

Horne JA, Östberg O. A self-assessment questionnaire to determine morningness-eveningness in human circadian rhythms. Int J Chronobiol. 1976;4(2):97-110.

Ising HK, Veling W, Loewy RL, Rietveld MW, Rietdijk J, Dragt S, et al. The validity of the 16-item version of the Prodromal Questionnaire (PQ-16) to screen for ultra high risk of developing psychosis in the general help-seeking population. Schizophr Bull. 2012;38(6):1288-96.

Krakow BJ, Melendrez DC, Johnston LG, Clark JO, Santana EM, Warner TD, et al. Sleep Dynamic Therapy for Cerro Grande Fire evacuees with posttraumatic stress symptoms: A preliminary report. J Clin Psychiat. 2002;63(8):673-84.

Kroenke K, Spitzer RL, Williams JB. The PHQ-9: validity of a brief depression severity measure. J Gen Intern Med. 2001;16(9):606-13.

Kroenke K, Spitzer RL, Williams JB, Lowe B. An Ultra-Brief Screening Scale for Anxiety and Depression: The PHQ–4. Psychosomatics. 2009; 50(6); 613-621.

Kroenke K, Spitzer RL, Williams JB, Lowe B. The Patient Health Questionnaire Somatic, Anxiety, and Depressive Symptom Scales: a systematic review. Gen Hosp Psychiatry. 2010;32(4):345-59.

Morin CM, Belleville G, Belanger L, Ivers H. The Insomnia Severity Index: Psychometric Indicators to Detect Insomnia Cases and Evaluate Treatment Response. Sleep. 2011;34(5):601-8.

Mundt JC, Marks IM, Shear MK, Greist JH. The Work and Social Adjustment Scale: a simple measure of impairment in functioning. Br J Psychiatry. 2002;180:461-4.

Ronald A, Sieradzka D, Cardno AG, Haworth CM, McGuire P, Freeman D. Characterization of psychotic experiences in adolescence using the specific psychotic experiences questionnaire: findings from a study of 5000 16-year-old twins. Schizophr Bull. 2014;40(4):868-77.

FINAL

Schults KF and Grimes DA. Multiplicity in clinical trials I: endpoints and treatments. Lancet. 2005; 365:1591-1595.

Spitzer RL, Kroenke K, Williams JB, Lowe B. A brief measure for assessing generalized anxiety disorder: the GAD-7. Arch Intern Med. 2006;166(10):1092-7.

Tennant R, Hiller L, Fishwick R, Platt S, Joseph S, Weich S, et al. The Warwick-Edinburgh mental well-being scale (WEMWBS): development and UK validation. Health Qual Life Out. 2007;5.

FINAL

12 Appendices

Appendix I. Outcome assessment schedule

The full battery of questionnaires (topics 1-7 below) amounts to 129 questions in total. Demographic questions (topic 1) will be asked at baseline only. Other mental health variables will be asked at baseline and week 22 (topic 7). Remaining questionnaire topics (2-6) will be repeated at all time points (baseline, 3, 10, 22 and 33 weeks).

**Demographics**	Date of Birth<br>Gender<br>Ethnicity<br>A-Level Results<br>Undergraduate versus post-graduate<br>Name of University<br>Subject of study at university<br>Parents post code	
**Sleep**	Sleep Condition Indicator (Espie et al. 2014).	Eight item screening measure for Insomnia Disorder. Scores range from 0-32 with higher scores indicating better sleep. A clinical cut off of ≤16 has been shown to correctly identify 89% of those with probable insomnia disorder. The measure has good internal consistency (Cronbach's alpha = .86) (Espie et al. 2014). An additional item (regarding early morning waking) will also be asked (secondary).
	Insomnia Severity Index (Bastien et al., 2001, Morin et al. 2011)	A seven item screening measure for Insomnia Disorder. Scores range from 0-28 with higher scores indicating increased insomnia symptomatology. The measure has a clinical cut off of >15. The measure has adequate internal consistency, good convergent validity and is sensitive to detect change in perceived sleep difficulties (Morin et al. 2011).
	Disturbing Dreams and Nightmare Severity Index (Krakow et al. 2002)	The scale comprises 5 questions asking about nights per week with nightmares, nightmare count per week, awakenings due to bad dreams, severity of nightmare problem and intensity of actual nightmares. It is scores from 0-37; a score of ≥10 predicts the presence of a clinically salient nightmare complaint (Krakow et al. 2002). Sensitivity to change has not been assessed.

FINAL

	The Morningness-Eveningness Questionnaire (Horne and Östberg 1976)<br><br>Whether the participant has a bed partner / room mate.	One question will be used form this measure to screen for chronotype. The question will ask participants whether they consider themselves a morning versus evening type person on a 4 point scale, higher scores indicate increased morningness.<br><br>One question to determine the presence of a bed partner / room mate.
**Impairment in Functioning**	Work and Social Adjustment Scale (Mundt et al 2002,)	A scale to assess participants' perceived impairment in functioning. The scale will be adapted so that it asks specifically about impairment attributable to sleep. The scale has good internal consistency (alpha = .70-.94), test re-test reliability (correlation .73) and correlates well with clinician assessments (.81-.86) (Mundt et al. 2002).
**Psychotic-like experiences**	Specific Psychotic Experiences Questionnaire (SPEQ) – hallucinations subscale (Ronald et al. 2014)<br><br>Green Paranoid Thoughts Scale (GPTS) (Green et al. 2008)<br><br>Prodromal Questionnaire – 16 item version (Ising et al. 2012)	The scale includes nine items measuring hallucinatory experiences across a range of sensory modalities. The scale ordinarily ranges from not at all to daily, with no time reference period. Within this study participants will be asked to consider the period over the past two weeks only and the scale is adjusted from 0 (not at all) to 5 (more than once per day).<br><br>The GPTS part B will be used to assess persecutory ideas over the past two weeks (this is adapted from the original version which assesses experiences over the past month). This is a 16 item scale with each item rated from 1 (not at all) to 5 (totally). The persecutory ideas sub-scale has excellent internal consistency (chronbach's alpha = .90), good test re-test reliability (correlation coefficient = .81) and good convergent and criterion validity (Green et al. 2008).<br><br>This questionnaire has 16 items to assess psychotic symptoms. A score of 6 or more positively answered items has 87% specificity and 87% sensitivity to correctly classify at-risk mental state in a help-seeking sample (Ising et al. 2012).
**Mood**	Patient Health	The Patient Health Questionnaire – nine item

	Questionnaire – 9 item questionnaire (Kroenke et al. 2001)<br><br>Generalised Anxiety Disorder-7 item questionnaire (Spitzer et al. 2006)<br><br>Altman mania scale (Altman et al. 2007)	questionnaire (PHQ-9) yields scores ranging from 0-27. We will ask participants to report on symptoms over the past week. A score of ≥10 has good sensitivity (88%) and specificity (88%) for detecting depressive disorders and is sensitive to change (Kroenke et al. 2001, Kroenke et al. 2010).<br><br>The GAD-7 is a seven item questionnaire assessing symptoms of anxiety. Scores range from 0-21. We will ask participants to report on symptoms over the past week. A cut off of ≥10 provides maximal specificity and sensitivity for a diagnosis of Generalised Anxiety Disorder (Spitzer et al. 2006, Kroenke et al. 2010).<br><br>The Altman self-report mania scale is a five item questionnaire assessing manic symptoms. We will ask participants to report on symptoms over the past week. The scale ranges from 0-20 and a score of 6 or higher yields good sensitivity (87.3%) and specificity (85.5%) for a manic or hypomanic disorder, is sensitive to change and has good test-retest reliability (Altman et al. 2007).
**Wellbeing**	Wellbeing Warwick-Edinburgh Mental Wellbeing Scale (Tennant et al. 2007)	This scale measures mental wellbeing using 14 items. Scores range from 14-70. The measure has good internal consistency (chronbach's alpha = .91), high correlation with other mental health and wellbeing scales and good test re-test reliability (.83) (Tennant et al. 2007).
**Other mental health variables**	Current contact with mental health services<br>Current diagnosis<br>Current prescribed medications<br>Current receipt of psychological therapy	

Appendix II. Flow diagram of trial participants

The sequence of events will be as follows:

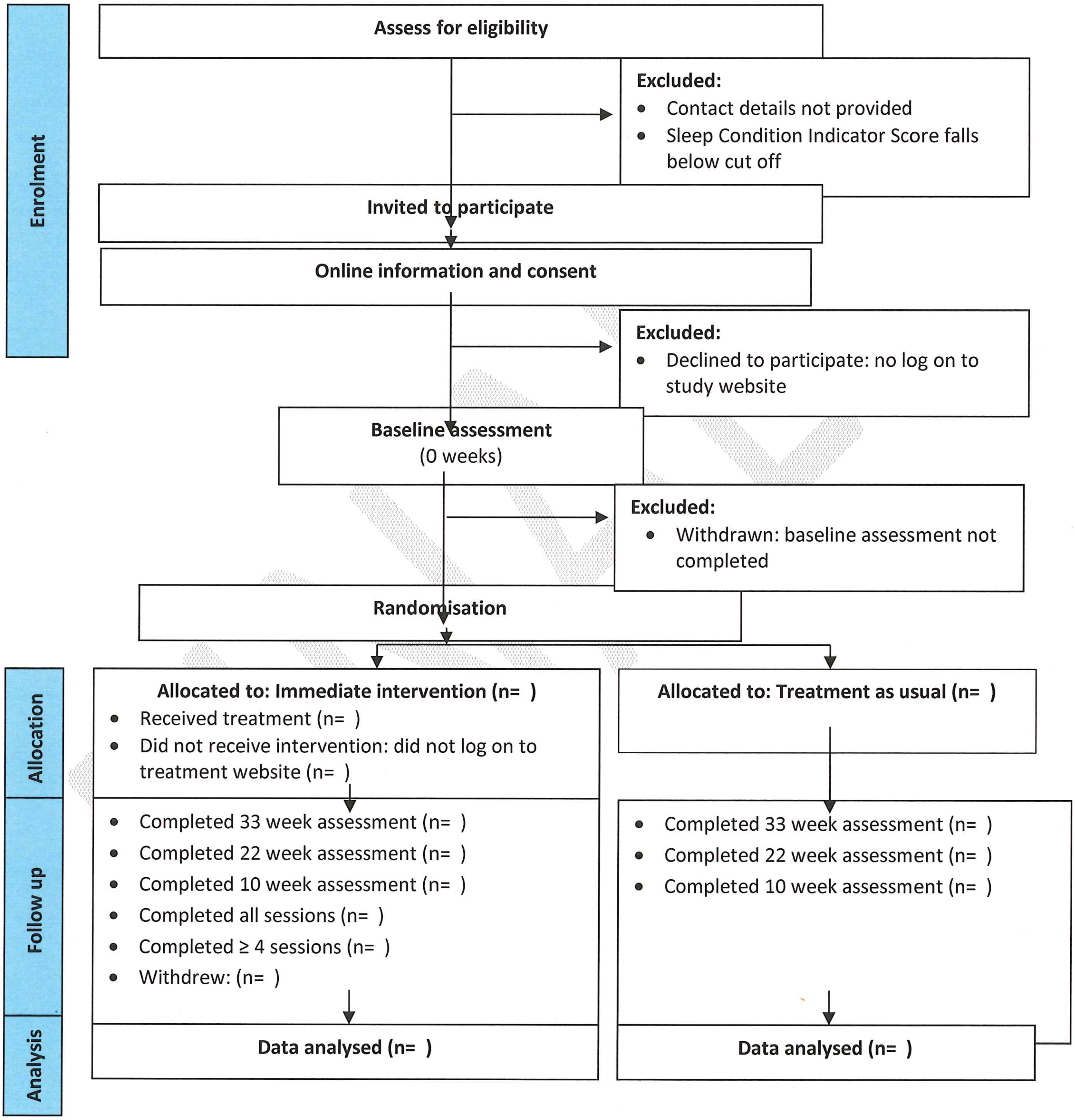

Supplement: Supplementary appendix 2 [file mmc2.pdf]
